# HES6 drives a critical AR transcriptional programme to induce castration-resistant prostate cancer through activation of an E2F1-mediated cell cycle network

**DOI:** 10.1002/emmm.201303581

**Published:** 2014-04-14

**Authors:** Antonio Ramos-Montoya, Alastair D Lamb, Roslin Russell, Thomas Carroll, Sarah Jurmeister, Nuria Galeano-Dalmau, Charlie E Massie, Joan Boren, Helene Bon, Vasiliki Theodorou, Maria Vias, Greg L Shaw, Naomi L Sharma, Helen Ross-Adams, Helen E Scott, Sarah L Vowler, William J Howat, Anne Y Warren, Richard F Wooster, Ian G Mills, David E Neal

**Affiliations:** 1 Uro-Oncology Research Group, Cancer Research UK Cambridge Institute, University of Cambridge, Li Ka Shing CentreCambridge, UK; 2 Department of Urology, Addenbrooke's HospitalCambridge, UK; 3 Cancer Research UK Cambridge Institute, University of Cambridge, Li Ka Shing CentreCambridge, UK; 4 Bioinformatics Core Facility, Cancer Research UK Cambridge Institute, University of Cambridge, Li Ka Shing CentreCambridge, UK; 5 Histopathology/ISH Core Facility, Cancer Research UK Cambridge Institute, University of Cambridge, Li Ka Shing CentreCambridge, UK; 6 Department of Pathology, Addenbrooke's HospitalCambridge, UK; 7 Cancer Metabolism Drug Discovery, Oncology R&DCollegeville, PA, USA; 8 Prostate Cancer Research Group, Nordic EMBL Partnership, Centre for Molecular Medicine Norway (NCMM), University of OsloOslo, Norway; 9 Departments of Cancer Prevention and Urology, Institute of Cancer Research and Oslo University HospitalsOslo, Norway; 10 Department of Oncology, University of CambridgeCambridge, UK

**Keywords:** androgen receptor, castrate-resistant prostate cancer, gene expression signature, HES6, PLK1

## Abstract

Castrate-resistant prostate cancer (CRPC) is poorly characterized and heterogeneous and while the androgen receptor (AR) is of singular importance, other factors such as c-Myc and the E2F family also play a role in later stage disease. HES6 is a transcription co-factor associated with stem cell characteristics in neural tissue. Here we show that HES6 is up-regulated in aggressive human prostate cancer and drives castration-resistant tumour growth in the absence of ligand binding by enhancing the transcriptional activity of the AR, which is preferentially directed to a regulatory network enriched for transcription factors such as E2F1. In the clinical setting, we have uncovered a HES6-associated signature that predicts poor outcome in prostate cancer, which can be pharmacologically targeted by inhibition of PLK1 with restoration of sensitivity to castration. We have therefore shown for the first time the critical role of HES6 in the development of CRPC and identified its potential in patient-specific therapeutic strategies.

## Introduction

Prostate cancer is the most common non-cutaneous malignancy in men in western countries (CRUK, 2010). Death results from the development of castrate-resistant prostate cancer (CRPC). Several mechanisms have been proposed to explain how this aggressive phase of the disease develops. Most depend on continued androgen receptor (AR) signalling in the context of enhanced secondary pathways, while others are independent of conventional AR activity (Pienta & Smith, 2005; Lamb *et al*, 2014). This heterogeneity explains why, although virtually all primary locally advanced prostate cancer can be treated effectively with androgen deprivation therapy (ADT), CRPC remains difficult to treat, and those therapies that have been developed are only effective for a limited duration in a limited set of patients (Yap *et al*, 2011). It is therefore critical that we understand more about the driving processes in CRPC, so that patient-specific mechanisms can be targeted.

Various oncogenic factors have been implicated in the natural history of prostate cancer. The AR has generally been accepted as the pre-eminent driving protein in prostate cancer and, even in hormone resistant prostate cancer, it is still implicated in several escape mechanisms based on a non-dihydrotestosterone-bound-AR (Pienta & Smith, 2005; Lamb *et al*, 2014). c-Myc has been found to be amplified in more than half of refractory prostate cancers (Bernard *et al*, 2003; Clegg *et al*, 2011) and has been identified as one of the key players in cell potency (Takahashi *et al*, 2007), but the factors which mediate its downstream effects in late-stage prostate cancer are still not well understood. Deregulation of E2F activity and early cell cycle progression has also been implicated in aggressive prostate cancer (Sharma *et al*, 2010), but the precise role of E2Fs in cancer is dependent on a balanced interplay with other regulatory factors and these interactions in prostate cancer need to be elucidated.

The study of transcription factors in cell pluripotency has shown that a small number of master regulator transcription factors can radically change cell phenotype (Takahashi *et al*, 2007). HES6 has been identified as a regulator of stem cell fate, specifically in neurogenesis (Kageyama *et al*, 2005; Murai *et al*, 2011), myogenesis (Malone *et al*, 2011) and gastrulation (Murai *et al*, 2007, 2011). In addition, it has been described as a marker of the neuroendocrine phenotype in prostate cancer (Vias *et al*, 2008) and in metastatic breast cancer (Hartman *et al*, 2009). Most recently, work demonstrating the cooperation of HIF-1α and FoxA2 in metastatic neuroendocrine prostate cancer has identified HES6 as an important factor in the development of these tumours (Qi *et al*, 2010).

Here, we evaluate the role of HES6 in CRPC and propose a mechanism of action in which HES6 induces persistent AR signalling and enhanced E2F1-mediated cell cycle activity. In the clinical setting, we describe a HES6-associated gene signature that correlates with poor outcome and outline a possible therapeutic strategy to target this programme of resistance.

## Results

### HES6 is regulated by AR and c-Myc

**Figure 1:**
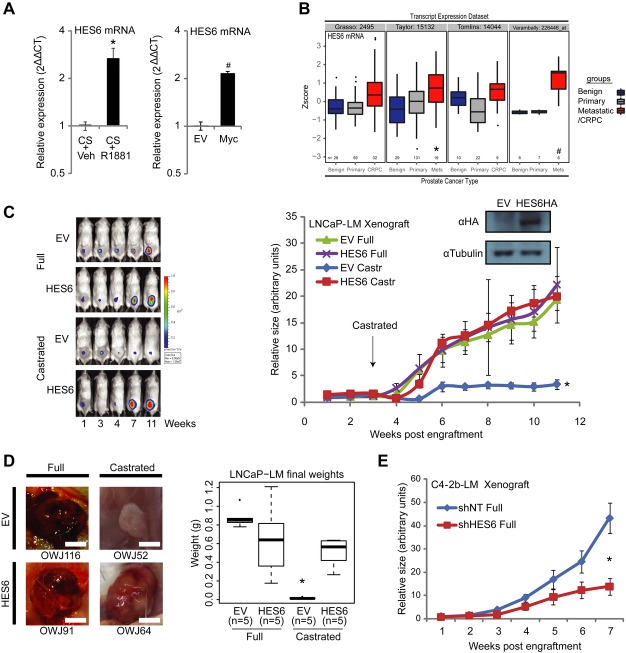
HES6 induces castration-resistant growth. A Changes in HES6 mRNA levels on alteration of AR and c-Myc activity in LNCaP cells, shown by quantitative PCR. Cells were starved in Charcoal Stripped Foetal Bovine Serum (CS) for 72 h and treated with 10 nM R1881 (AR agonist) for 16 h to check the AR regulation of HES6. Cells transfected with a pcDNA4-Myc vector were grown in Full Serum (FS) conditions; *n* = 3, error bars represent mean ± s.e.m.; *P = 0.016, ^#^*P* = 6.9E-6 by *t*-test. B Box and whisker dot plot showing HES6 expression is increased in metastatic disease (Mets) and castrate-resistant prostate cancer (CRPC) in several public datasets (Tomlins *et al*, 2005; Varambally *et al*, 2005; Taylor *et al*, 2010; Grasso *et al*, 2012). *P = 8.8E-6, ^#^*P* = 7.9E-5 by limma with Benjamini–Hochberg adjustment for multiple testing for comparison of Mets to Benign. C Xenografts of HES6-overexpressing LNCaP cells are resistant to castration. LNCaP cells expressing luciferase/YFP (LNCaP-LM) and transduced to overexpress HES6 were grafted subcutaneously (2 × 10^6^ cells) in NSG mice, and growth was monitored by bioluminescent imaging for 11 weeks. Host mice were castrated at 3 weeks (grafts approximately 100 mm^3^); *n* = 5; error bars represent mean ± s.e.m; *P* = 0.0002 by *t*-test at week 11 for comparison of EV Castr to HES6 Castr. EV = Empty vector. Full = testes present. Castr = castration. D Visual appearance of harvested grafts and mean weights. One xenograft of each group is shown as an illustration. Evidence of significant difference in mean graft weight in castrated conditions (0.017 ± 0.009 g versus 0.503 ± 0.158 g). Scale bars = 5 mm; *n* = 5; error bars represent mean ± s.e.m.; *P = 0.0001 by *t*-test. E Androgen-insensitive C4-2b-LM xenografts showed growth attenuation with constitutive lentiviral knock-down of HES6 (shHES6) compared to non-targeting controls (shNT). *n* = 5; error bars represent mean ± s.e.m.; *P = 0.004 by *t*-test at week 7. See also Supplementary Figs S1–S3. Source data are available online for this figure.

Using the androgen-dependent LNCaP cell line and chromatin immunoprecipitation sequencing (ChIPseq), we showed that two potent oncogenes in prostate cancer, the AR (Massie *et al*, 2011) and c-Myc, bind upstream of the HES6 coding sequence (Supplementary Fig S1A) to positively regulate levels of HES6 mRNA (Fig 1A, Supplementary Fig S1B and C) and confirmed that overexpression of c-Myc overcomes growth inhibition by bicalutamide (Bernard *et al*, 2003) (Supplementary Fig S1D), an effect that can be reversed by knock-down of HES6 (Supplementary Fig S1E).

### HES6 initiates androgen-independent growth

HES6 levels are found to be raised in several independent clinical datasets comparing aggressive disease (metastases or CRPC) with primary tumour and benign disease (Fig 1B). Using the bicalutamide/castration-resistant derivatives LNCaP-Bic (Hobisch *et al*, 2006), C4-2 and C4-2b cells (Thalmann *et al*, 1994) as models of CRPC, we found evidence of significant increases in HES6 transcript levels in the C4-2 derivatives compared to LNCaP controls (Supplementary Fig S2A). To test the functional relevance of HES6 in the transition to CRPC, we stably overexpressed HA-tagged HES6 in androgen-sensitive prostate cancer LNCaP and DuCaP cells, which induced reduced sensitivity to bicalutamide (Supplementary Fig S2B–D). We then proceeded to surgical castration using luciferase-labelled LNCaP cells (LNCaP-LM) subcutaneously injected into the flanks of NOD-SCID gamma (NSG) mice. Graft size was monitored by bioluminescent imaging over 3 months. Overexpression of HES6 was sufficient to maintain normal tumour growth (Fig 1C) after castration. Control grafts in castrated conditions were small and pale, while the comparable HES6 grafts were large and vascular (Fig 1D). Knock-down of HES6 by lentiviral shRNA in C4-2b cells (Supplementary Fig S2E) led to significant attenuation of tumour growth in both full and castrated conditions (Fig 1E and Supplementary Fig S2F). These data show that HES6 is sufficient to produce a castration-resistant phenotype.

### Nuclear AR and cell proliferation are maintained in HES6 grafts in castrate conditions

We next assessed intensity of nuclear AR and cell proliferation by immunohistochemical analysis of the LNCaP grafts. Normally, the AR shuttles in and out of the nucleus according to the extent of ligand activation, and following removal of testosterone, the AR localizes to the cytoplasm (Azzi *et al*, 2006; Cutress *et al*, 2008). We demonstrated cytoplasmic relocation of the AR in the native mouse prostate (Supplementary Fig S3A) and in control grafts following castration. By contrast, the AR was predominantly nuclear in castrated HES6 xenografts (Supplementary Fig S3A, quantified in Supplementary Fig S3B), suggesting a role for nuclear AR in HES6-driven castration-resistant grafts. Staining using the proliferation marker Ki67 showed uniform increases in the castrated HES6-overexpressing tissue (Supplementary Fig S3A, quantified in Supplementary Fig S3C).

### HES6 rescues AR activity at a subset of Androgen Receptor Binding Sites (ARBS) which is also enhanced in CRPC

**Figure 2:**
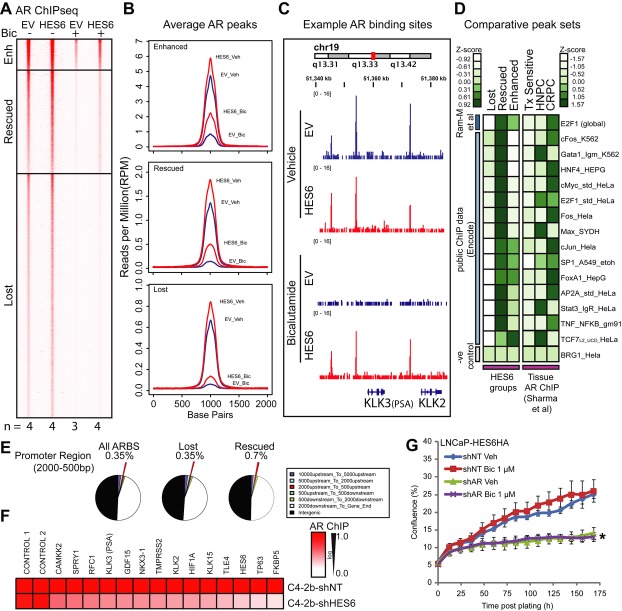
HES6 induces castration-resistant growth. A Heatmap of normalized signal at peak summits in a window ± 1 kb. Binding site classes were established by grouping highly reproducible peak calls into those that were still present with empty vector (EV) in 1 μM bicalutamide (Bic) but “enhanced” by HES6, “rescued” by HES6 or “lost” in both conditions. Average binding site location was established from mean location of peak summits across biological replicates and intensity of signal calculated as mean of the RPM normalized read counts. All conditions were in biological quadruplicate as shown with the exception of EV in bicalutamide which was in triplicate. Veh = vehicle (ETOH). B The mean normalized AR signal as profiles across binding sites seen in (A). This illustrates the global differences between conditions within the different AR binding site classes. C Example of binding events at KLK2 and KLK3 (PSA) where binding is markedly reduced with bicalutamide but rescued with overexpression of HES6. D Heatmap showing binding site co-occurrence for “lost,” “rescued” and “enhanced” groups compared to global E2F1 binding sites as well as Encode Consortium ChIPseq sets for well-known regulatory transcription factors. Comparisons were also made with our human tissue AR ChIPseq (Sharma *et al*, 2013). Co-occurrence was calculated as the total base-pair overlap of occupancy between the test and query binding site sets normalized to the total base-pair occupancy of the query set. To account for the differing lengths of the test sets, co-occurrence scores were normalized to Z-scores within each test set. Tx = treatment, HNPC = hormone naïve prostate cancer. E Genomic distribution analysis of “rescued” versus “lost” ARBS compared to whole genome shows increased AR binding in proximal promoters. F Selected AR binding sites were validated by ChIP in the castrate-resistant C4-2b derivative of LNCaP cells. Knock-down of HES6 reduced AR binding enrichment at all selected sites compared to control sites with no ChIPseq evidence of AR binding. *n* = 3; CAMKK2 *P* = 0.021, SPRY1 *P* = 0.026, RFC1 *P* = 0.056, KLK3 *P* = 0.041, GDF15 *P* = 0.008, NKX3-1 *P* = 0.006, TMPRSS2 *P* = 0.017, KLK2 *P* = 0.023, HIF1A *P* = 0.031, KLK15 *P* = 0.009, TLE4 *P* = 0.026, HES6 *P* = 0.018, TP63 *P* = 0.01, FKBP5 *P* = 0.006 by *t*-test. G HES6-overexpressing LNCaP cells were transduced with a pSicoR lentivirus to stably knock-down AR (40% knock-down). Constitutive lentiviral knock-down of the AR limits HES6-overexpressing LNCaP growth and abrogates the HES6-driven bicalutamide-resistant phenotype. Vehicle (Veh) is ETOH and bicalutamide (Bic) 1 μM; *n* = 3; error bars represent mean ± s.e.m.; *P = 0.023 by *t*-test for comparison of shAR Bic 1 μM to shNT Bic 1 μM. See also Supplementary Fig S4 and Tables S1 and S2.

In order to explain our discovery of nuclear AR with increased levels of HES6 in castration, we measured AR binding to chromatin by ChIPseq. We found during bicalutamide treatment that HES6 was able to maintain many AR binding sites that were lost in the EV control cells. In such inhibitory conditions, we were able to divide AR binding sites (ARBS) into three distinct classes: first, those peaks that were lost during AR inhibition in both EV and HES6 cells were defined as “lost”; second, those that disappeared in EV during bicalutamide treatment but were clearly present with HES6 overexpression were defined as “rescued”; third, those that were never totally lost in the EV control condition with bicalutamide but were clearly enhanced by the presence of high HES6 were defined as “enhanced” (Fig 2A, Supplementary Table S1). Of these 3 groups, we observed the strongest difference in average peak height between HES6 and EV in those defined as “rescued” (Fig 2B). This included well-known AR responsive genes such as KLK2 and KLK3 (Fig 2C, Supplementary Fig S4A). We made comparison with ChIPseq data for other transcription factors including E2F1, c-Myc, FOXA1 and STAT3 (Fig 2D, accessed through Encode Consortium), and found a strong enrichment in the “rescued” group, raising the possibility of concerted activity between the AR and these factors. Furthermore, analysis of previous data assessing ARBS in human tissue (Sharma *et al*, 2013) revealed a similar pattern of transcription factor overlap in CRPC tissue, as well as untreated and potentially aggressive primary prostate cancer (Fig 2D). Interrogation of the ARBS in an unbiased manner with MEME (Machanick & Bailey, 2011) identified similar motif enrichment (Supplementary Fig S4B and Table S2). Analysis of ARBS genomic distribution uncovered a twofold increase in ARBS located 500–2,000 bp upstream of transcriptional start sites (TSS) in the “rescued” set, consistent with a small shift of AR binding towards proximal promoters (Fig 2E).

We confirmed that this persistent AR signalling was HES6-dependent by demonstrating evidence of reduced AR binding at several selected ARBS with stable knock-down of HES6 in C4-2b cells (Fig 2F). Finally, we verified that HES6-driven androgen insensitivity is still AR-dependent by knocking down the AR in HES6-overexpressing cells, with reversal of the phenotype (Fig 2G, Supplementary Fig S4C). Collectively this suggests modulation of the AR regulome by HES6.

### HES6, AR and E2F1 co-operate in driving a cell cycle-related tumour-enhancing network

**Figure 3:**
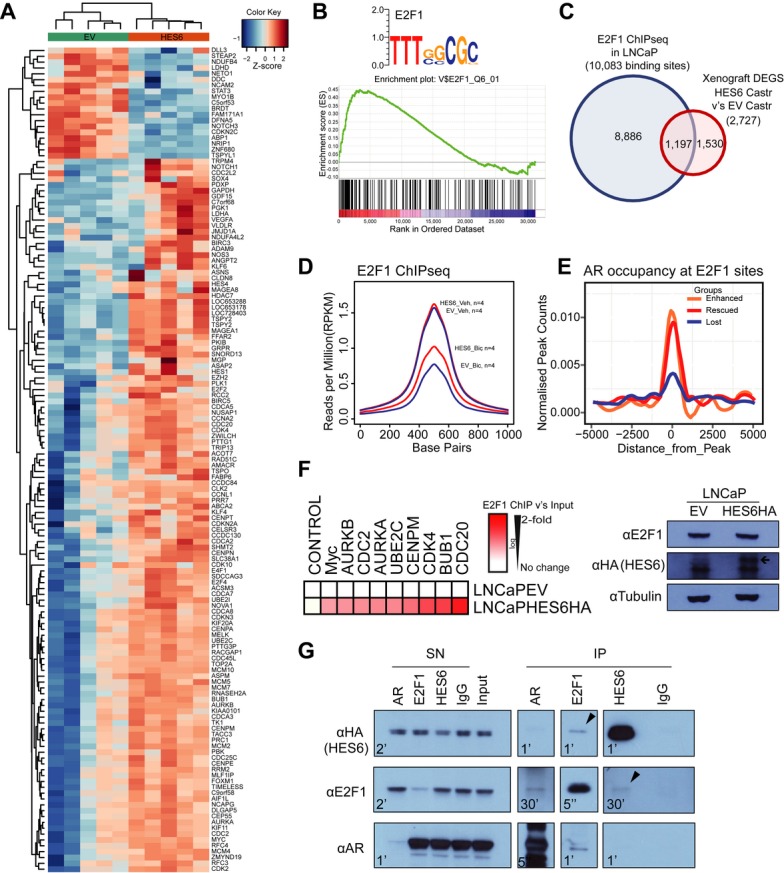
HES6 enhances a cell cycle network through concerted activity with E2F1 and AR. A Selection of differentially expressed genes (DEGs) in castrated xenografts of HES6-overexpressing LNCaP cells (orange) versus castrated EV controls (green); *n* = 5. B GSEA motif analysis revealed enrichment of E2F1 in DEGs associated with HES6-overexpressing cells. Xenograft GSEA-NES-value = 2.25 and cells *in vitro*GSEA-NES-value = 2.09. C Overlap of the DEGs from castrated HES6-overexpressing LNCaP xenografts with E2F1 targets. Nearest gene (± 25 kb) overlap with DEGs from castrated (Castr) HES6-overexpressing LNCaP xenografts is shown; hypergeometric *P*-value = 2.53E-46. D Average enrichment of E2F1 at target sites is increased with HES6 compared to EV in bicalutamide (Bic) but not vehicle (Veh, ETOH). All ChIPseqs *n* = 4. E AR ChIPseq occupancy at E2F1 binding sites is increased in HES6 “enhanced” and “rescued” peaks compared to “lost” (see Fig 2). F Heatmap showing E2F1 ChIP. HES6 increased binding of E2F1 to differentially expressed genes in the castrated xenografts of HES6-overexpressing LNCaP cells. Each ChIP *n* = 3; Myc *P* = 0.052, AURKB *P* = 0.057, CDC2 *P* = 0.12, AURKA *P* = 0.087, UBE2C *P* = 0.165, CENPM *P* = 0.049, CDK4 *P* = 0.025, BUB1 *P* = 0.068, CDC20 *P* = 0.087 by *t*-test. G HES6 and AR co-immunoprecipitate with E2F1. IPs for AR, E2F1, HES6 and control IgG were performed using HES6HA-overexpressing LNCaP cell extracts and blotted for HA, E2F1 and AR. *n* = 5. One representative experiment is shown for illustration. SN = supernatant. IP = immunoprecipitate. Different time exposures of the same membrane are shown for each blot section to better visualize the whole experiment and are indicated in the lower-left corner (‘= minutes and  “= seconds). Arrows indicate co-IP of HES6 and E2F1. See also Supplementary Figs S5–S8. and Table S3. Source data are available online for this figure.

We used expression microarrays to identify differentially expressed genes (DEGs) associated with HES6 overexpression in the castration-resistant xenografts (Fig 3A, Supplementary Fig S5) and Ingenuity Pathway Analysis (IPA, Ingenuity® Systems, http://www.ingenuity.com) to infer the functional consequences (Supplementary Fig S6A). Surprisingly, we did not find any evidence for a neuroendocrine phenotype (Supplementary Fig S5) but rather a predominance for cell cycle pathways. A combined cell cycle network from the input gene list revealed several dominant clusters including E2Fs, CDKs, Cyclins and Aurora kinases (AURK) (Supplementary Fig S6B).

In order to identify potential transcriptional intermediaries, we assessed enriched binding motifs by *in silico* analysis (Supplementary Fig S6C and Table S3) of the DEGs from castrated xenografts as well as HES6-overexpressing LNCaP cells. This identified strong enrichment for E2F family binding sites and specifically for E2F1 (Fig 3B). Furthermore, we found a strong overlap (44%) between DEGs in castrated xenografts and E2F1 transcriptional targets identified by ChIP-seq in LNCaP cells (Fig 3C). To identify whether the presence of HES6 has a specific effect on E2F function, we performed ChIPseq for E2F1 (*n* = 4) in LNCaP cells and found that HES6 increased average E2F1 binding activity in the presence of bicalutamide (Fig 3D). Given our previous findings suggesting a shift in AR binding towards promoter regions when “rescued” by HES6 in bicalutamide, we investigated whether AR and E2F1 could be interacting and whether HES6 was able to modulate such interaction. We found that the ARBS belonging to the “enhanced” and “rescued” groups showed a clear increase in AR occupancy at E2F1 binding sites (Fig 3E). Enrichment of E2F1 binding in cells overexpressing HES6 was validated at promoters of selected E2F1 transcriptional targets (Fig 3F and Supplementary Fig S7) and was found not to be a consequence of increased E2F1 protein levels (Fig 3F and Supplementary Fig S5). We reasoned that HES6 may be enhancing E2F activity and investigated potential interactions between HES6 and E2F1. Given the persistence of nuclear AR with HES6, we also evaluated interactions with the AR. We demonstrated physical interaction between HES6 and E2F1, as well as between the AR and E2F1 (Fig 3G). These findings indicate that HES6 may enhance concerted E2F1 and AR activity in androgen-deprived conditions.

E2F1 is a final common regulator for G1/S cell cycle transition (Cam & Dynlacht, 2003). We therefore assessed by FACS analysis the effect of overexpressing HES6 on cell cycle distribution in LNCaP cells and observed an increase in the fraction of cells in both S and G2/M phases (Supplementary Fig S8A). To test whether this was due to more rapid initiation of cell cycle activity, we observed cell cycle fractions after synchronization (Supplementary Fig S8B-D) and found more rapid cell cycle re-entry in HES6-overexpressing cells in both normal conditions and bicalutamide.

### HES6 has a corresponding signature associated with aggressive clinical disease

**Figure 4:**
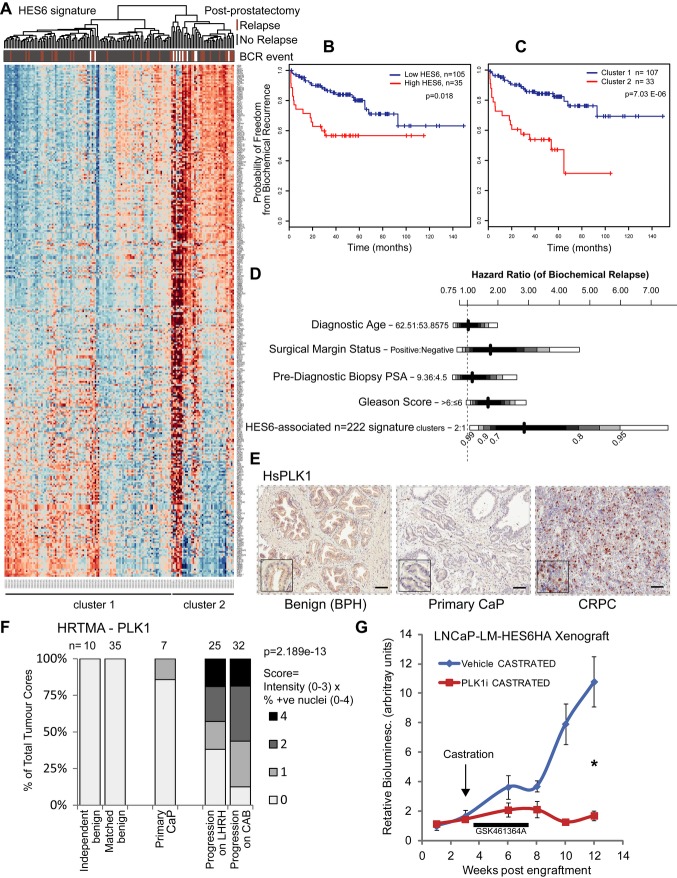
HES6 is associated with aggressive clinical disease targetable by PLK1i. A HES6-associated signature (*n* = 222, see Supplementary Fig S10 and Table S4 for generation of *n* = 222 HES6-associated signature) used to cluster 140 prostatectomy specimens (Taylor *et al*, 2010). See also Supplementary Fig S9. B Kaplan–Meier biochemical relapse-free survival analysis by recursive partitioning to high and low expression of HES6. Logrank *P*-value. C Kaplan–Meier biochemical relapse-free survival analysis by two cluster partitioning based on expression of HES6-associated signature (*n* = 222). Logrank *P*-value. D Graphical representation of proportional hazard ratios for biochemical relapse (BCR) calculated by Cox regression models used to estimate the association with relapse of several key variables: age, surgical margin status, PSA, Gleason score and the clusters generated by the HES6-associated signature. Both the signature and the Gleason score were shown to have an independent prognostic effect (*P* = 0.0071 and *P* = 0.0208), but not PSA (*P* = 0.7384), SMS (*P* = 0.1467) and age of diagnosis (*P* = 0.9180). See also Supplementary Fig S11. E Protein expression of HES6-responsive gene PLK1 shows strong staining in CRPC alone, compared to primary prostate cancer (CaP) and benign control (benign prostatic hyperplasia, BPH). Scale bars represent 250 μm. Magnified windows = 100 μm^2^. F Quantification of PLK1 expression in a hormone-relapsed tissue microarray (HRTMA). Cores were taken from 45 men with varying degrees of androgen insensitivity undergoing channel transurethral resection of the prostate (chTURP), including both tumour areas and neighbouring benign regions where available. A number of independent benign and primary CaP men were also included. Tumour cores were scored by two trained individuals assessing staining intensity and percentage of positive nuclei. There was increased PLK1 staining in men resistant to androgen therapy; *P*-value = 2.189E-13 by Fisher's exact test. LHRH = luteinizing hormone releasing hormone. CAB = complete androgen blockade. G LNCaP-LM-HES6HAHA xenografts were sensitized to castration on treatment with selective polo-like kinase 1 (PLK1) inhibitor GSK461364A 50 mg/kg administered i.p. at a dose rate of q2dx12 versus vehicle. Host mice were castrated at 3 weeks (grafts approximately 100 mm^3^); *n* = 8; error bars represent mean ± s.e.m.; *P = 0.007 by Mann–Whitney *U*-test at week 12. See also Supplementary Figs S9–S12 and Table S4.

To assess the clinical relevance of these findings, we used publicly available data (Varambally *et al*, 2005) to make comparisons between benign, primary and metastatic prostate cancer. We found that the cell cycle genes associated with HES6 expression (from Fig 3A) were strongly represented in the metastatic group (Supplementary Fig S9). We interrogated these data to identify transcripts most strongly correlated with HES6 and selected the top 1.5% according to a cut-off correlation coefficient of 0.9 (Supplementary Fig S10A). From these 788 genes, we selected those that were also present in the castrated HES6-overexpressing LNCaP xenograft DEG list. The combination of these two sets of data (one from human samples, one from xenografts overexpressing HES6) generated a “HES6-associated signature” of 222 transcripts (Supplementary Table S4). In order to validate this signature, we studied three additional publicly available datasets (Tomlins *et al*, 2005; Taylor *et al*, 2010; Grasso *et al*, 2012) and a published cell cycle progression signature (CCP) in CRPC (Cuzick *et al*, 2011). Allowing for differences in array coverage, we found that genes on this HES6-associated signature list were consistently enriched in DEG lists comparing metastatic or castrate-resistant tumours with benign or normal tissue (Supplementary Fig S10B and C). These HES6-correlated genes effectively clustered men into two groups following radical prostatectomy (Taylor *et al*, 2010) (Fig 4A). Kaplan–Meier analysis at a mean follow-up of 4.5 years (range 2–149 months) showed clear partitioning for men with low versus high HES6 (Fig 4B). Use of the *n* = 222 HES6-associated signature further enhanced clustering and was even more effective in differentiating those patients destined for early relapse (Fig 4C). This signature is more powerful in this capacity than PSA and Gleason score (Fig 4D and Supplementary Fig S11). In the absence of good HES6 antibodies that are reliable for immunohistochemistry (Hartman *et al*, 2009), we checked protein expression of the HES6-associated cell cycle regulator polo-like kinase 1 (PLK1) in representative samples of benign, primary prostate cancer and CRPC tissue (Fig 4E), identifying strong expression in CRPC tissue only. We quantitatively assessed PLK1 expression in a large novel human tissue microarray incorporating 61 men, some of whom had androgen naïve prostate cancer while others had hormone-relapsed disease. We found clear evidence for an increase in PLK1 expression in the prostates of men with CRPC (Fig 4F).

### Selective pharmacological inhibition of this mechanism of androgen independence

Finally, we investigated whether inhibition of HES6-driven cell cycle regulating genes could alter growth in these castration-resistant HES6-overexpressing cells. We found that these cells, though resistant to bicalutamide, were responsive to inhibition with the PLK1 inhibitor GSK461364A (Gilmartin *et al*, 2009) with a greater than 50% reduction in cell proliferation (Supplementary Fig S12A). Indeed, concomitant application of this inhibitor with bicalutamide further reduced the rate of cell growth suggesting a synergistic effect to rescue the androgen-dependent phenotype. These findings were recapitulated *in vivo* with intraperitoneal injection of drug dramatically slowing castrate-resistant growth of LNCaP-LM-HES6 xenografts (Fig 4G). PLK1 inhibition also reduced growth of castrate-resistant AR-positive cells (C4-2b) and AR-negative cells (PC3) (Supplementary Fig S12B and C) with greater effects on PC3 cells on isogenic introduction of the AR (Nelius *et al*, 2007). This suggests that PLK1 inhibition reverses HES6-driven castration resistance and raises the possibility of synergy with AR activity.

## Discussion

Clinical progression to castrate-resistant prostate cancer (CRPC) remains a major clinical problem and is the cause of death for most men dying of prostate cancer. Half of men on LHRH monotherapy will progress within 2 years of treatment (Hellerstedt & Pienta, 2002), and survival after the onset of metastatic CRPC does not usually extend beyond 6–12 months (Petrylak *et al*, 2004). Recent developments in second-/third-line prostate cancer therapeutics with wide-ranging modes of action have reinforced the concept that castrate-resistant prostate cancer is a heterogeneous disease with multiple mechanisms of resistance (de Bono *et al*, 2010, 2011; Kantoff *et al*, 2010; Nilsson *et al*, 2011; Scher *et al*, 2012; Ryan *et al*, 2013). It is therefore important that cancer researchers delineate fully these different mechanisms. Our study outlines a mechanism of resistance centred on a single transcription co-factor and shows how hypothesis-driven investigation of cell function, combined with large-scale genomic correlation, can deliver a candidate oncogenic factor in a meaningful biological context with the potential to uncover new therapeutic avenues that could improve the treatment of men who are no longer responsive to current drugs.

We have identified HES6 as a transcription co-factor that is able to alter prostate cancer cell phenotype so fundamentally that these cells are able to grow in the absence of testosterone. HES6 is better known for its functions in the nervous system, where it is thought to promote neuronal differentiation (Kageyama *et al*, 2005; Murai *et al*, 2011), but its role in the prostate cell is not yet well understood. Here we have seen that HES6, a driver of androgen independence, is itself regulated by the AR. This suggests that the AR regulome includes factors that are not necessarily required for growth in a normal environment, but which can be recruited in an altered environment where the cell has to rely on other pathways for survival (Mills, 2014). Recently, it has been shown that ETS factors such as ERG markedly increase AR binding in mouse prostate tissue and mediate robust transcriptional changes in PTEN null prostate cancer cells (Chen *et al*, 2013). In our study, we describe how, during castration or AR inhibition, HES6 overexpression can modulate the AR regulome, maintaining chromatin binding at a proportion of ARBS in the absence of hormone stimulation. We also show c-Myc to be another regulator of HES6 transcription, and so have identified two potent oncogenes involved upstream of HES6 in this mechanism of androgen independence.

Our study identifies E2F1 as an important intermediary in this process, with E2F-regulated cell cycle factors accounting for a large proportion of differentially expressed genes in these castrate-resistant tumours. This cell cycle enhancing framework fits with other recent studies providing evidence for the centrality of cell cycle genes in explaining mechanisms of resistance (Sharma *et al*, 2010) and predicting poor survival in the clinical setting (Cuzick *et al*, 2011). We identify a novel interaction between HES6 and E2F1 and E2F1 and the AR and show enhancement of E2F1 activity that seems to result from protein complex formation rather than increased E2F1 expression as previously found in breast cancer (Hartman *et al*, 2009). We propose that this mechanism may account for maintained prostate cancer cell growth through concerted activity with the AR in HES6-driven androgen insensitivity, where the AR persistently binds to chromatin at a subset of binding sites and seems to be essential for the survival of the resistant cells, which cannot withstand AR knock-down. This finding is consistent with previous work assessing the impact of AR knock-down on castration-resistant derivatives of this model (Snoek *et al*, 2009).

Although we did not find evidence for a classical neuroendocrine phenotype, recent studies have suggested a broader relevance for neuroendocrine differentiation with powerful transcription factors such as Myc and cell cycle regulators such as AURKA being implicated in development of therapy resistant tumours bearing neuroendocrine characteristics (Beltran *et al*, 2011).

We used a robust independent model to derive a HES6-associated gene signature which contained several factors, including UBE2C (Wang *et al*, 2009), EZH2 (Xu *et al*, 2012), and cell cycle progression factors such as CDC20, TK1, PLK1 and PTTG1 already shown to be important in CRPC (Cuzick *et al*, 2011). We have found that HES6-driven genes are strongly associated with aggressive human prostate cancers at their presentation, demonstrating that this phenomenon is not confined to the laboratory setting or to cell lines alone. Furthermore, in patients undergoing radical treatment, this signature is associated with early biochemical relapse after surgery.

We have identified PLK1 as a possible target within this gene signature with an inhibitor giving promising results in rescuing an androgen-sensitive phenotype and halting CRPC cell growth. When combined with our data suggesting poor survival in patients who have raised levels of HES6 and its associated genes, this raises the possibility that such molecular information could be used to stratify patients for trials of a PLK1 inhibitor. In future, a more personalized treatment strategy could be pursued with men who display a high-risk molecular signature being monitored closely and offered early adjuvant treatment, while those at lower risk could be managed more expectantly in order to avoid unnecessary intervention.

Taken together, our results identify the critical role of the HES6 transcription co-factor in maintaining AR activity in CRPC with concerted enhancement of E2F1 activity to maintain cell growth in the absence of testosterone and thereby offer a fresh treatment paradigm in combating this disease.

## Materials and Methods

Detailed methodology and details of antibodies, primers, probes and oligonucleotides are described in Supplementary Methods.

### Plasmids

c-Myc was amplified from an IMAGE clone (Lawrence Livermore National Laboratory, Livermore, CA) and then inserted at the BamH1 restriction site of the lentiviral inducible pLVX-Tight-Puro vector (Clontech, Mountain View, CA) by IN-Fusion Advantage (Clontech) homologous fusion. Lentiviral pLVX-Teton-Genet vector (Clontech) was used to stably express the tetracycline-controlled transactivator. The human HES6 cDNA sequence was amplified and inserted into the retroviral vector pBabe-puro (Cell Biolabs, San Diego, CA). For knock-down of HES6, annealed oligonucleotides were inserted into the lentiviral pSicoR vector (Ventura *et al*, 2004) with a puromycin resistance cassette. Knock-down of the AR was achieved using the lentiviral pSicoR vector with a blasticidin resistance cassette.

### Mice

Immunocompromised NSG male mice (Charles River, Wilmington, MA) were used for tumour implantation. GSK461364A was administered intraperitoneally 50 mg/kg at a dose rate of q2dx12. Vehicle was water with 2% Cremophor-EL and 2% N,N-dimethylacetamide (DMA) (Sigma-Aldrich, St. Louis, MO). Mice were maintained in the Cancer Research UK Cambridge Institute Animal Facility. All experiments were performed in accordance with national guidelines and regulations, and with the approval of the animal care and use committee at the institution.

### Gene expression data

IlluminaBeadChip HumanWG-12 (version 3 and 4; Illumina, San Diego, CA) were used for the gene expression. Publicly available prostate cancer data sets were downloaded from GEO (Gene Expression Omnibus). Biological function and network generation was performed using Ingenuity Pathway Analysis (IPA, Ingenuity Systems, Redwood City, CA). For enrichment and motif analysis, we used GSEA (Mootha *et al*, 2003; Subramanian *et al*, 2005).

### Human prostate tissue samples

Ethical approval for the use of samples and data collection was granted by the local Research Ethics Committee under ProMPT (Prostate Mechanisms for Progression and Treatment) “Diagnosis, investigation and treatment of prostate disease” (MREC 01/4/061).

### Statistics

Data from independent experiments were reported as the mean ± s.e.m. Student's *t*-test analysis or Mann–Whitney *U*-test were performed to determine statistical significance in replicate comparisons and Fisher's exact test for proportionate analyses.

### Study approval

All animal procedures were carried out in accordance with University of Cambridge and Cancer Research UK guidelines under UK Home Office project licenses 80/2301 and 80/2435. For human material, informed written consent was received from participants prior to inclusion in the study under ethics committee number MREC 01/4/061.

### Accession numbers

Study data are deposited in NCBI GEO under accession number GSE36526.

## The paper explained

### Problem

Prostate cancer is the most common non-cutaneous cancer in men. Radical prostatectomy or hormone therapy is commonly used to treat early stage or androgen-sensitive disease. However, relapse remains a serious clinical problem with approximately one-third of men developing recurrent disease within 5 years after prostatectomy and half of men developing hormone resistance within 2 years of commencement of hormone treatment. We noticed that HES6 levels tend to be high in aggressive disease and wished to discover whether this transcription co-factor plays a role in the development of resistance and could be used for stratification of targeted treatment.

### Results

In this study, we report that HES6 maintains AR chromatin binding at a critical subset of sites, which are enriched for cell cycle regulatory genes under the control of E2F1, induces resistance to anti-androgens and castration and predicts poor outcome in the clinical setting. We also identify a protein interaction between the AR and E2F1 and between HES6 and E2F1 and show that by drugging a kinase within the HES6 regulome, namely PLK1, we are able to restore response to anti-androgens.

### Impact

This study demonstrates the relevance of HES6 in the development of androgen resistance and brings forward our understanding of how other factors co-operate with the master regulator of prostate cancer, the AR, to maintain essential pathways for cell survival. A novel set of end-stage human prostate cancers have been assessed in this study, raising the possibility that judicious combination of hormone therapy with inhibition of the HES6 regulome offers a new treatment paradigm for high-risk prostate cancer patients who can be selectively identified and targeted for early treatment.
