# Supplementary material for: HES6 drives a critical AR transcriptional programme to induce castration-resistant prostate cancer through activation of an E2F1-mediated cell cycle network
Source: EMBO Mol Med. 2014 Apr 14;6(5):651–61. doi: 10.1002/emmm.201303581 (PMC4023887; doi:10.1002/emmm.201303581)
Supplement: Supplementary file 1 [file emmm0006-0651-sd1.pdf]

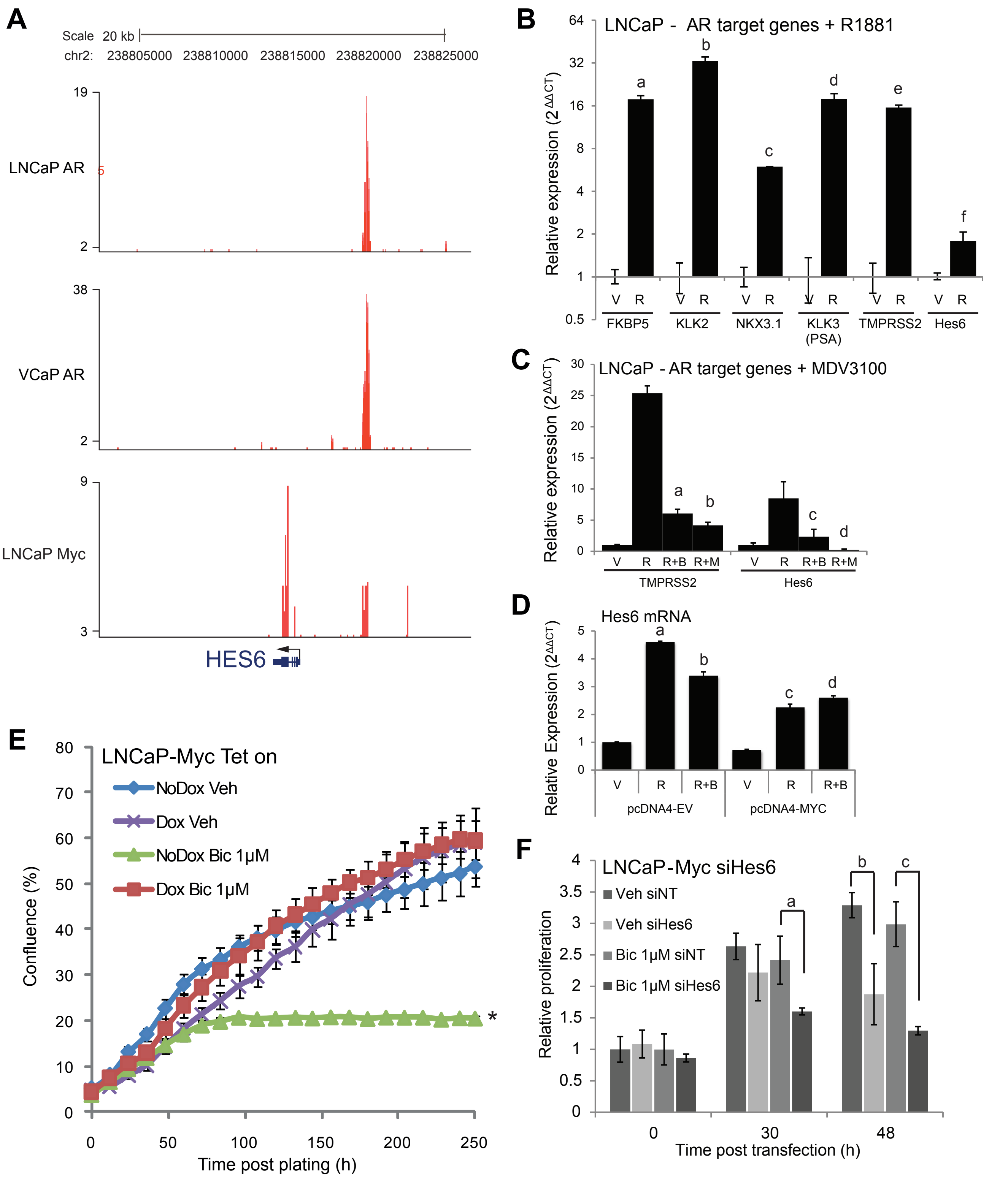

Figure S1, related to Figure 1. c-Myc and AR transcriptionally regulate Hes6 and c-Myc can drive bicalutamide resistance.

(A) ChIP-seq track in LNCaP cells showing AR and c-Myc binding upstream of the Hes6 5' UTR. AR tracks are also shown for VCaP cells, another androgen-responsive cell line with an amplification of the AR gene (Makkonen et al., 2011; Massie et al., 2011).

(B) Androgen target gene mRNA levels in response to R1881(R) compared to vehicle(V) on a charcoal stripped background; n = 3, error bars represent mean ± SEM. ^{a}p = 0.002, ^{b}p = 0.002, ^{c}p = 0.0004, ^{d}p = 0.003, ^{e}p=0.0008, ^{f}p = 0.064 compared to vehicle by t-test.

(C) Hes6 and TMPRSS2 (representative AR target) mRNA levels with enzalutamide 10µM (MDV3100, M) or bicalutamide 10µM (B); n = 3, error bars represent mean ± SEM. ^{a}p = 0.0001, ^{b}p = 8.1E-5, ^{c}p = 0.102, ^{d}p = 0.036 compared to R1881(R) by t-test.

(D) Hes6 mRNA levels with overexpression of c-Myc in the presence of R1881(R) and bicalutamide 1 µM (B) on a charcoal stripped background; n = 4, error bars represent mean ± SEM. ^{a}p = 8.4E-11, ^{b}p = 2.3E-6, ^{c}p = 1.2E-5, ^{d}p = 2.3E-7 compared to veh (V) by t-test.

(E) Lentiviral inducible overexpression of c-Myc in LNCaP cells drives bicalutamide-resistant growth. Vehicle (Veh) is ethanol (ETOH), bicalutamide (Bic) 1 µM and doxycycline (Dox) 2 µg/ml; n = 3, error bars represent mean ± SEM; *p=0.0001 for comparison of NoDox Bic. 1 µM to Dox Bic. 1 µM

(F) Transient knockdown of Hes6 in LNCaP-Myc overexpressing cells reduced cell growth and bicalutamide-resistance. All with doxycycline; n = 4, error bars represent mean ± SEM. ^{a}p = 0.079, ^{b}p = 0.036, ^{c}p = 0.003 by t-test.
