# Supplementary material for: HES6 drives a critical AR transcriptional programme to induce castration-resistant prostate cancer through activation of an E2F1-mediated cell cycle network
Source: EMBO Mol Med. 2014 Apr 14;6(5):651–61. doi: 10.1002/emmm.201303581 (PMC4023887; doi:10.1002/emmm.201303581)
Supplement: Supplementary file 2 [file emmm0006-0651-sd2.pdf]

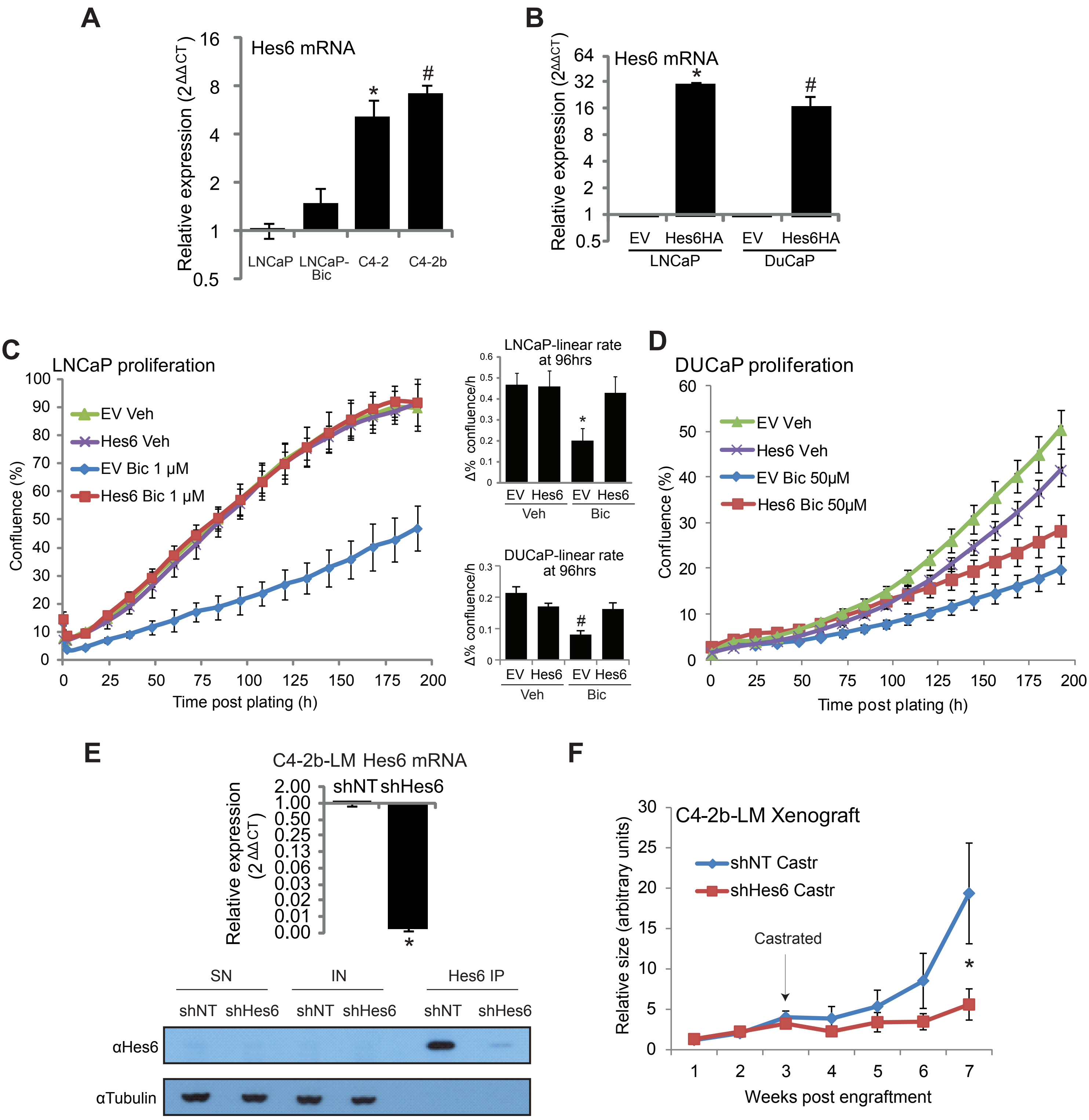

Figure S2, related to Figure 1. Overexpression of Hes6 induces bicalutamide resistance.

(A) Endogenous Hes6 levels are relatively increased in castration resistant derivatives of LNCaP cells. Shown by q-PCR. LNCaP-Bic = LNCaP cells with acquired bicalutamide resistance; C4-2 = xenografted LNCaP cells with acquired castration-resistance; C4-2b = osseous metastatic C4-2; n = 3, error bars represent mean ± SEM; *p = 0.006, $^{\#}$p = 0.0003 compared to LNCaP by t-test.

(B) LNCaP and DUCaP cells were transduced with a pBabe retrovirus for stable overexpression of HA-tagged Hes6. Hes6 mRNA levels measured by q-PCR are shown; n = 3, error bars represent mean ± SEM; *p = 2.9E-5, $^{\#}$p = 0.02 compared to EV by t-test.

(C&D) LNCaP and DUCaP cells with stable overexpression of Hes6 were grown in the presence bicalutamide. Vehicle (Veh) is ETOH and bicalutamide (Bic) 1 μM (LNCaP) or 50 μM (DUCAP). Proliferation rate was assessed at the mid-point of the growth curves by calculating the rate of change in confluence ; n = 4; error bars represent mean ± SEM. *p = 0.017, $^{\#}$p = 0.001 for comparison of EV Bic to Hes6 Bic by t-test.

(E) Luciferase-expressing C4-2b cells (C4-2b-LM) were transduced with a pSicoR lentivirus to stably knockdown Hes6. Hes6 mRNA levels measured by q-PCR and one representative blot are shown. Hes6 siRNA knockdown in LNCaP cells achieved similar levels. Enrichment of Hes6 by immunoprecipitation (IP) was used to validate knockdown of Hes6 in C4-2b-LM cells. IP was required due to the inability of current Hes6 antibodies to detect low levels of endogenous Hes6 in prostate cells. 240 mg of C4-2b cell extract per IP. SN = supernatant, IN = input; n = 3, error bars represent mean ± SEM; *p = 0.0002 by t-test.

(F) Androgen insensitive C4-2b-LM xenografts showed growth attenuation in castration with constitutive lentiviral knockdown of Hes6 (shHes6) compared to Non-Targeting controls (shNT). n = 5; error bars represent mean ± SEM; *p = 0.03 by t-test.
