# Supplementary material for: HES6 drives a critical AR transcriptional programme to induce castration-resistant prostate cancer through activation of an E2F1-mediated cell cycle network
Source: EMBO Mol Med. 2014 Apr 14;6(5):651–61. doi: 10.1002/emmm.201303581 (PMC4023887; doi:10.1002/emmm.201303581)
Supplement: Supplementary file 3 [file emmm0006-0651-sd3.pdf]

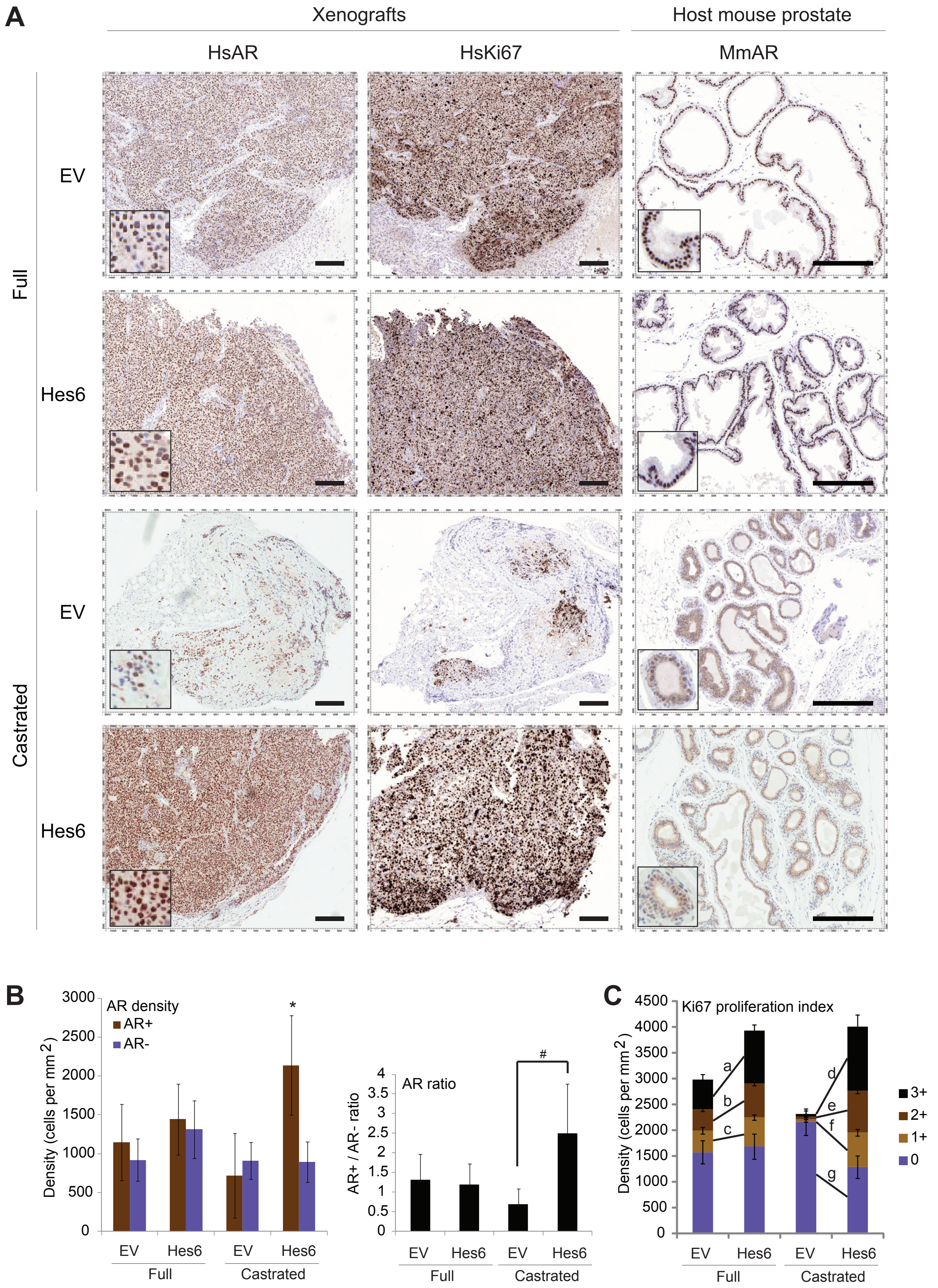

Figure S3, related to Figure 1. Nuclear AR and cell proliferation are maintained during castration in xenografts of Hes6-overexpressing LNCaP cells.

(A) Harvested EV control and Hes6-overexpressing LNCaP xenografts with representative immunohistochemistry of Human AR and Human Ki67. Analysis of synchronous host prostates with IHC for Mouse AR confirmed effective androgen blockade with cytoplasmic AR in both sets of host mice, compared to nuclear prostatic AR in non-castrated mice. Scale bars = 250 μm. Magnified windows = 100 μm^2.

(B) Cell density quantification of AR positive (AR+) and AR negative (AR-) nuclei by Ariol Imagestream in full and castrated mice. Ratios of AR+ to AR- nuclei are also shown; n = 5; error bars represent mean ± SEM; *p = 0.0001, $^{\#}$p = 0.008 compared to EV by t-test.

(C) Cell density quantification of Ki67 positive cells by Aperio point density scoring with cells binned into three groups (1+,2+,3+) according to intensity; n=5; error bars represent mean ± SEM; ^{a}p = 0.006, ^{b}p = 0.003, ^{c}p = 0.036, ^{d}p = 0.007, ^{e}p=0.0002, ^{f}p = 0.0001, ^{g}p = 0.012 by t-test.
