# Supplementary material for: HES6 drives a critical AR transcriptional programme to induce castration-resistant prostate cancer through activation of an E2F1-mediated cell cycle network
Source: EMBO Mol Med. 2014 Apr 14;6(5):651–61. doi: 10.1002/emmm.201303581 (PMC4023887; doi:10.1002/emmm.201303581)
Supplement: Supplementary file 4 [file emmm0006-0651-sd4.pdf]

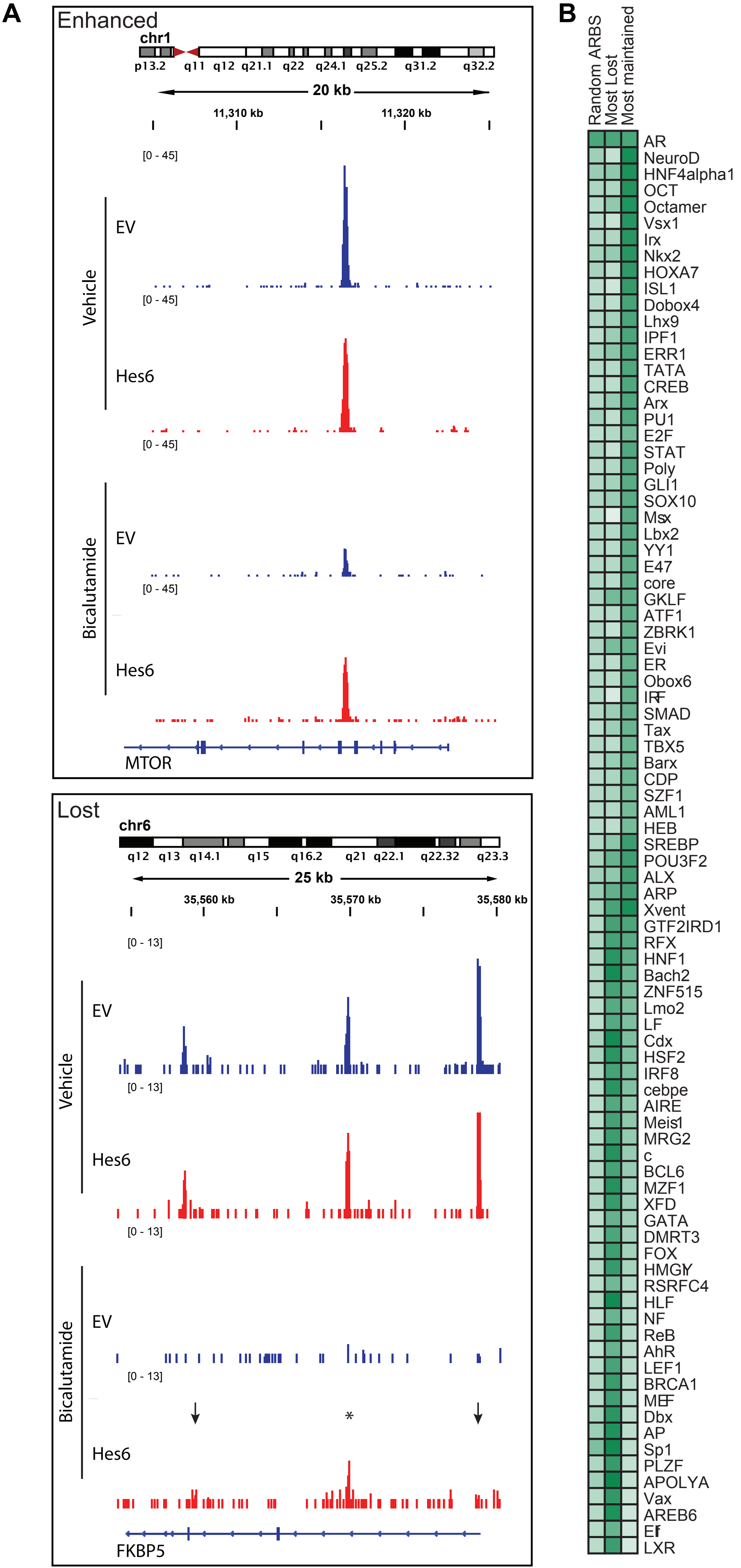

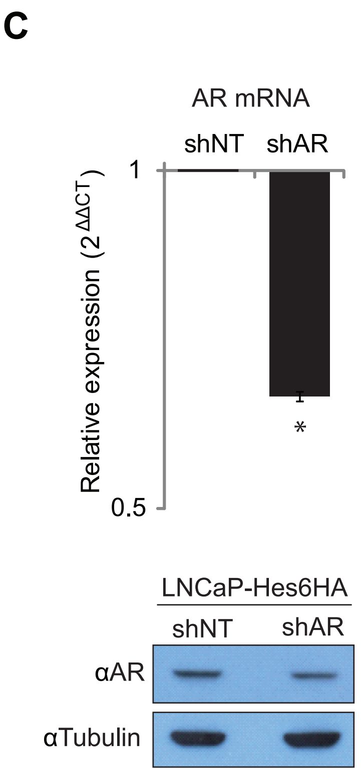

Figure S4, related to Figure 2. Hes6 overexpression induces variable changes in AR chromatin binding.

(A) Representative examples are given of ChIPseq tracks showing an ARBS at MTOR that is enhanced by Hes6 in bicalutamide and two ARBS at FKBP5 that are lost despite Hes6 in bicalutamide (indicated with an arrow) and one that is rescued (indicated with a star). The rescued ARBS was used to design the primers used for **Fig 2F**.

(B) Heatmap showing relative enrichment of transcription factors by MEME (http://meme.nbcr.net/meme/) when comparing the 1000 most-maintained ARBS, the 1000 most-lost and a random matched-size set across all ARBS.

(C) Hes6-overexpressing LNCaP cells were transduced with a pSicoR lentivirus to stably knockdown AR (40% knockdown). AR mRNA levels measured by q-PCR and one representative western blot are shown; n = 3, error bars represent mean ± SEM; *p = 0.0002 by t-test.
