# Supplementary material for: HES6 drives a critical AR transcriptional programme to induce castration-resistant prostate cancer through activation of an E2F1-mediated cell cycle network
Source: EMBO Mol Med. 2014 Apr 14;6(5):651–61. doi: 10.1002/emmm.201303581 (PMC4023887; doi:10.1002/emmm.201303581)
Supplement: Supplementary file 5 [file emmm0006-0651-sd5.pdf]

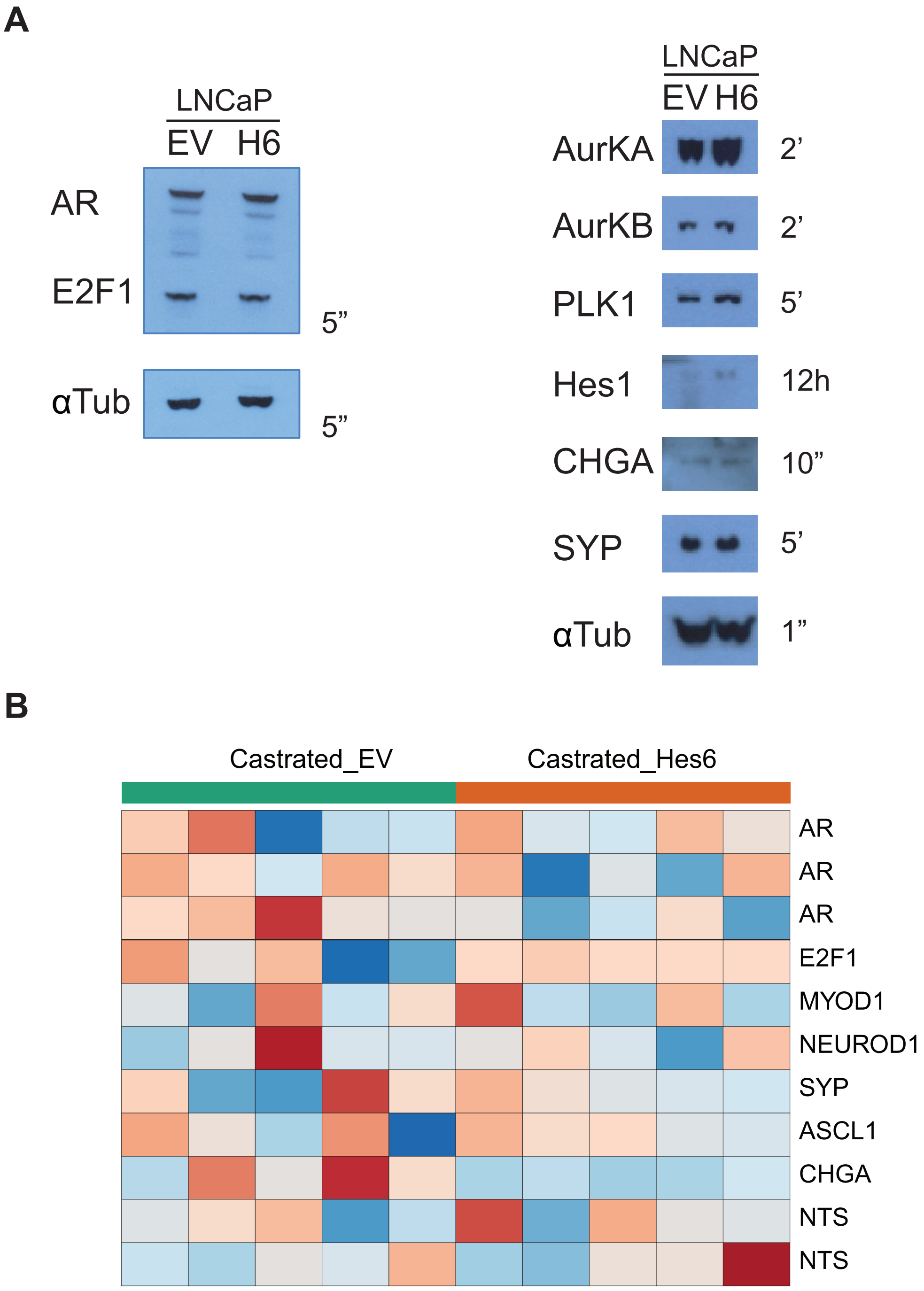


**Figure S5, related to Figure 3. No change in AR, E2F1 or neuroendocrine markers in response to Hes6 in castrate conditions.**
(A) Western blot protein analysis at varying levels of exposure as shown. There was no change in AR, E2F1, CHGA or SYP in LNCaP cells over-expressing Hes6. AURKA, AURKB, PLK1 and Hes1 levels were increased as found on transcript analysis. Cells cultured in bicalutamide 1μM. “ seconds; ‘ minutes. EV = Empty Vector. H6 = Hes6.
(B) Heatmap showing no change in transcript levels of neuroendocrine markers or of AR and E2F1 expression with Hes6-overexpressing LNCaP-LM xenografts.
