# Supplementary material for: HES6 drives a critical AR transcriptional programme to induce castration-resistant prostate cancer through activation of an E2F1-mediated cell cycle network
Source: EMBO Mol Med. 2014 Apr 14;6(5):651–61. doi: 10.1002/emmm.201303581 (PMC4023887; doi:10.1002/emmm.201303581)
Supplement: Supplementary file 6 [file emmm0006-0651-sd6.pdf]

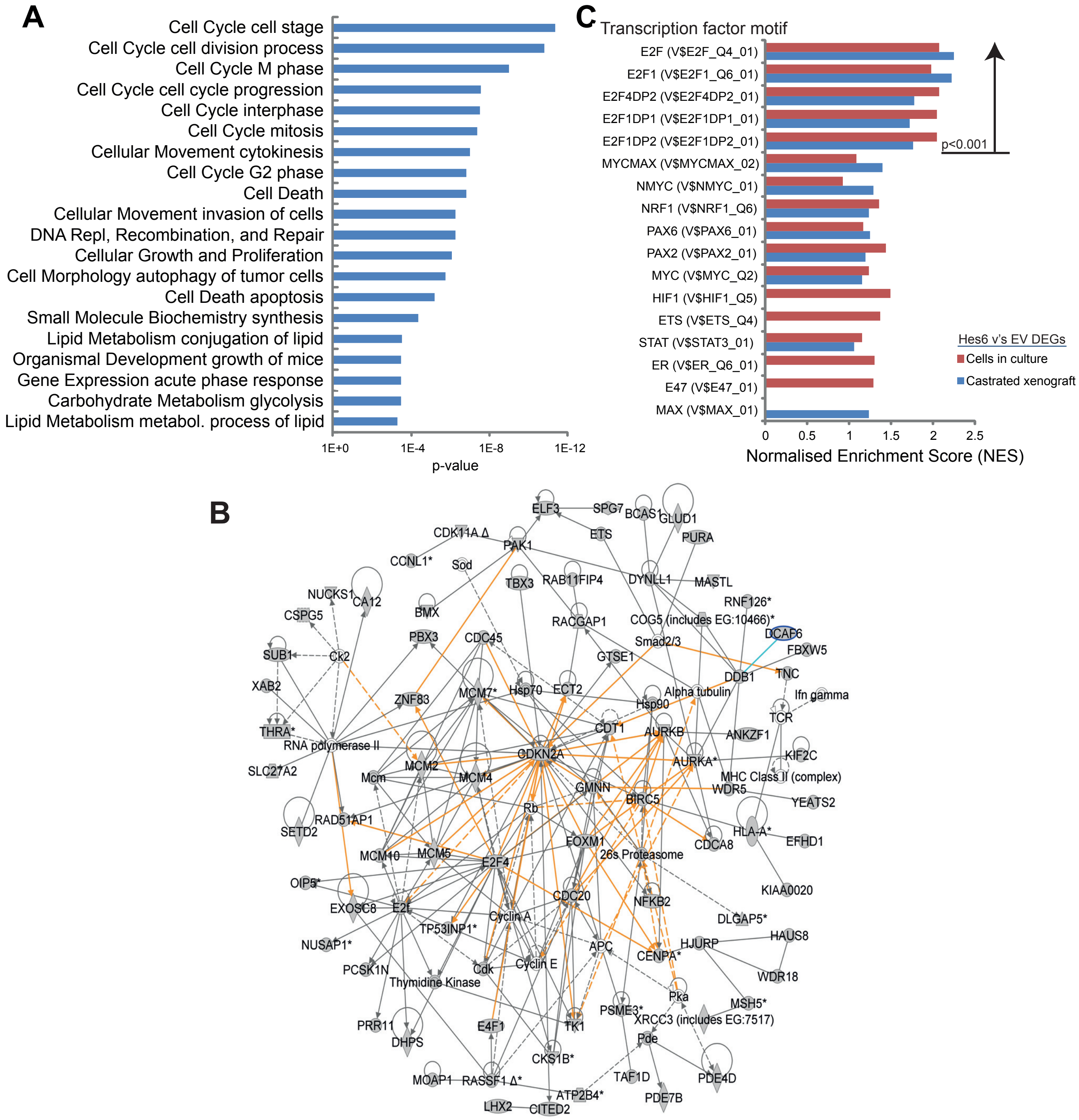

Figure S6, related to Figure 3. Pathway analysis reveals cell cycle ontology and E2F transcription factor enrichment in Hes6-overexpressing castrated xenografts.

(A) Ingenuity Pathway Analysis (IPA) top cellular and molecular functions for the differentially expressed genes (DEGs, cut-off $p<0.05$) in castrated xenografts of Hes6-overexpressing LNCaP cells versus castrated controls include cell cycle, cancer growth, and metabolic pathways; n = 5.

(B) Cell cycle network generated by unsupervised IPA interrogation of top up and down DEGs and merging of all networks with "cell cycle" as the predominant descriptive ontology (p = 4.45E-12).

(C) Transcription Factor (TF) motif enrichment analysis (GSEA, Broad Institute, Cambridge, MA) of differentially expressed genes (DEGs) in the Hes6-overexpressing LNCaP cells *in vitro* and in castrated Hes6 xenografts versus controls. Selected motifs are displayed with position weight matrices (PWMs) from those with the highest enrichment scores. E2F family members predominate. Normalised enrichment scores (NES) are shown.

See **Table S3** for full listings and p-values.
