# Supplementary material for: HES6 drives a critical AR transcriptional programme to induce castration-resistant prostate cancer through activation of an E2F1-mediated cell cycle network
Source: EMBO Mol Med. 2014 Apr 14;6(5):651–61. doi: 10.1002/emmm.201303581 (PMC4023887; doi:10.1002/emmm.201303581)
Supplement: Supplementary file 7 [file emmm0006-0651-sd7.pdf]

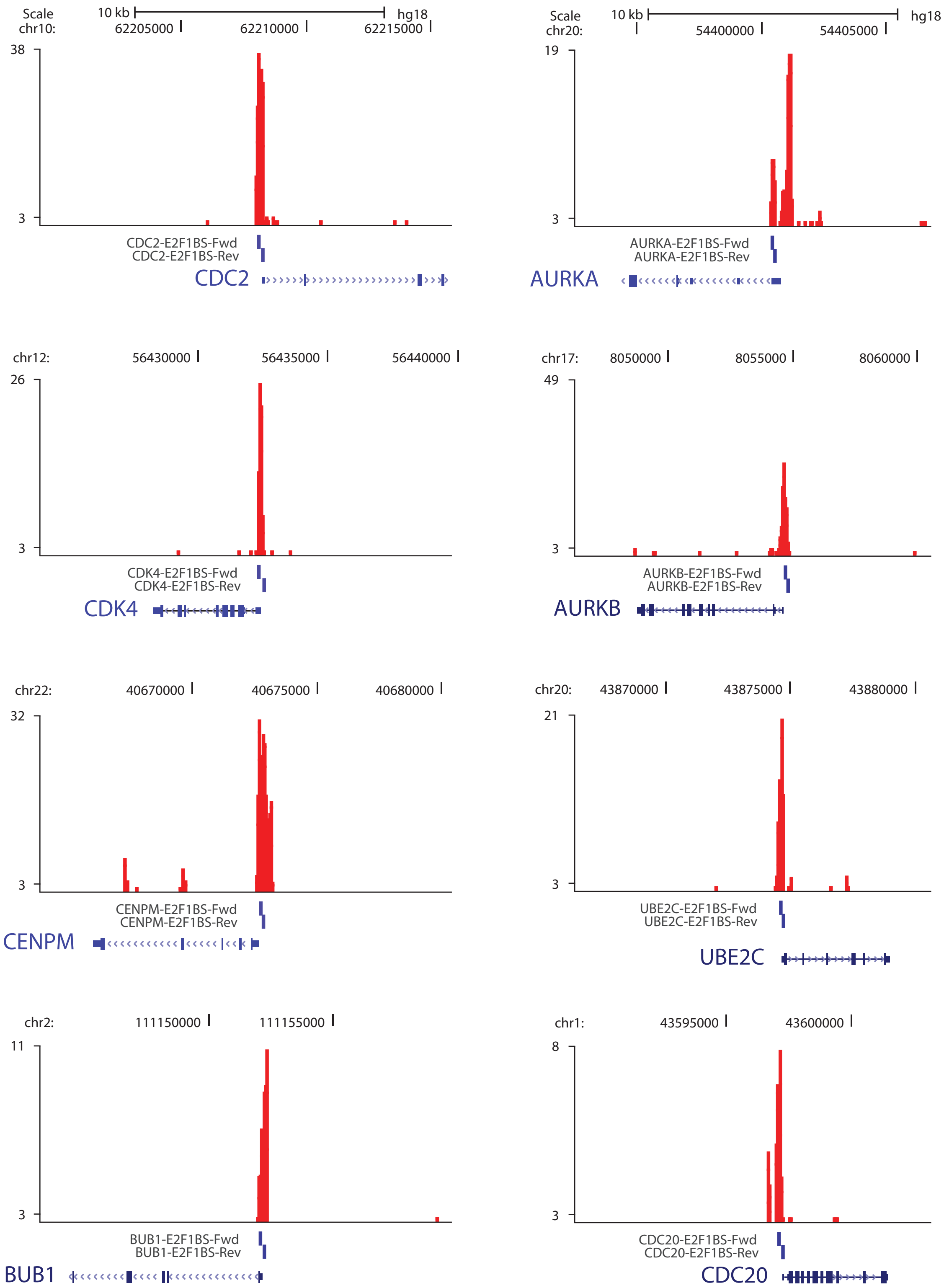

Figure S7, related to Figure 3. E2F1 binding sites and selection of PCR primers.
Specific E2F1 targets were identified and E2F1 binding sites determined from ChIP-seq in LNCaP cells. Regions of 100% specificity were selected as shown (”GENE”-E2F1BS-Fwd or Rev). BS = binding site. Fwd = sense primer. Rev = antisense primer.
