# Supplementary material for: HES6 drives a critical AR transcriptional programme to induce castration-resistant prostate cancer through activation of an E2F1-mediated cell cycle network
Source: EMBO Mol Med. 2014 Apr 14;6(5):651–61. doi: 10.1002/emmm.201303581 (PMC4023887; doi:10.1002/emmm.201303581)
Supplement: Supplementary file 8 [file emmm0006-0651-sd8.pdf]

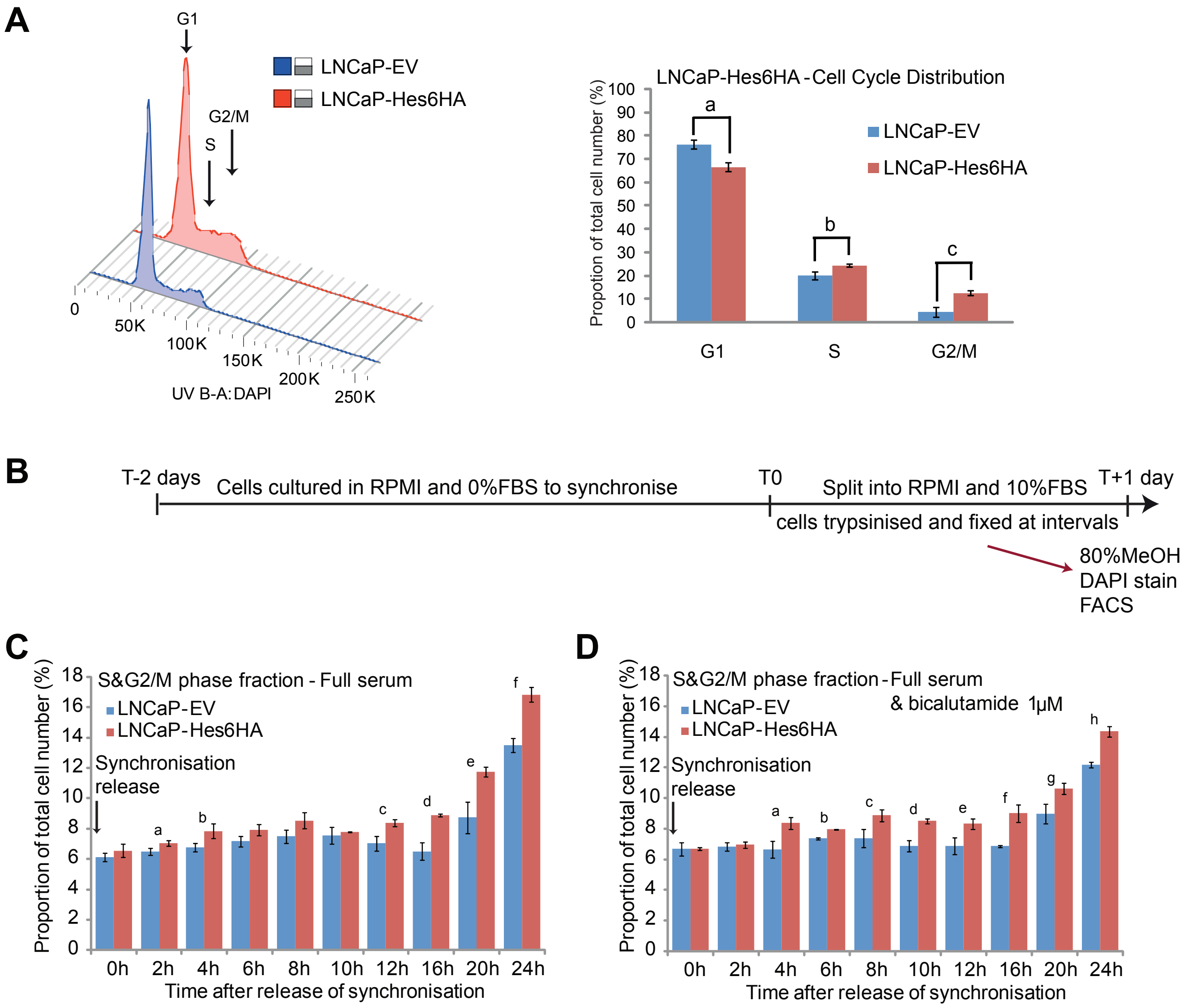

Figure S8, related to Figure 3. Hes6-overexpression induces more rapid cell cycle entry.

(A) Constitutive overexpression of Hes6 in unsynchronized LNCaP cells increases the fraction of cells exiting G1 phase and progressing to both S and G2M phase. Numerically confirmed with Flow-jo (v8.8.4) quantification. n = 3, error bars represent mean ± SEM; $^{a}p = 0.003$, $^{b}p = 0.008$, $^{c}p = 0.007$ by t-test.

(B) Cells were serum starved for 48 h and then released to cycle in different conditions.

(C) Hes6 increased the fraction of synchronized LNCaP cells exiting G1 phase, and expedited their transition to S and G2/M phase. Cultured in Vehicle (Veh) = ETOH; n = 3; error bars represent mean ± SEM; $^{a}p = 0.029$, $^{b}p = 0.027$, $^{c}p = 0.011$, $^{d}p = 0.002$, $^{e}p=0.008$, $^{f}p = 0.001$ comparing LNCaP-Hes6HA to EV by t-test

(D) Hes6 increased the fraction of synchronized LNCaP cells exiting G1 phase, and expedited their transition to S and G2/M phase. Cultured in bicalutamide (Bic) 1 μM; n = 3; error bars represent mean ± SEM; $^{a}p = 0.012$, $^{b}p = 0.0009$, $^{c}p = 0.022$, $^{d}p = 0.002$, $^{e}p=0.031$, $^{f}p = 0.003$, $^{g}p = 0.05$, $^{h}p = 0.0006$ by t-test.
