# Supplementary material for: HES6 drives a critical AR transcriptional programme to induce castration-resistant prostate cancer through activation of an E2F1-mediated cell cycle network
Source: EMBO Mol Med. 2014 Apr 14;6(5):651–61. doi: 10.1002/emmm.201303581 (PMC4023887; doi:10.1002/emmm.201303581)
Supplement: Supplementary file 9 [file emmm0006-0651-sd9.pdf]

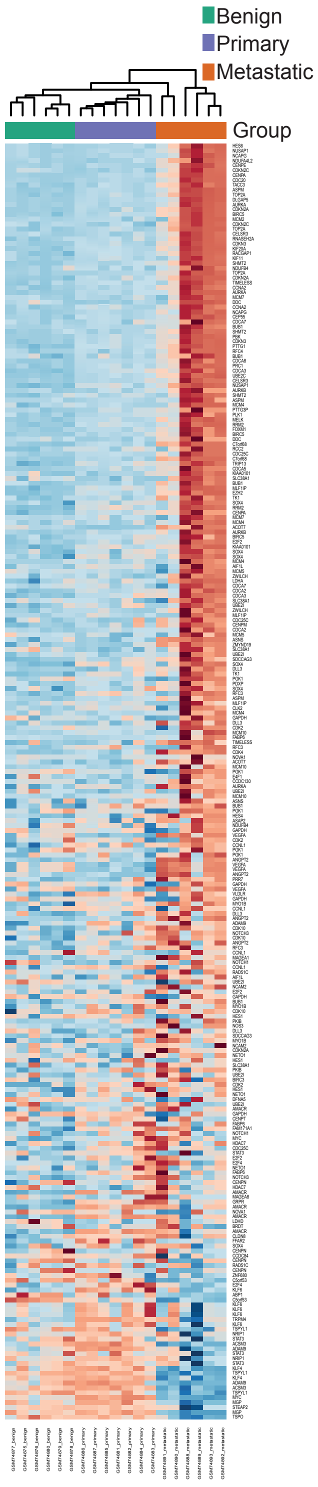

Figure S9, related to Figure 4. Hes6 xenograft differentially expressed genes (DEGs) are associated with aggressive clinical disease.

Selected Hes6 DEGS (from Figure 3A) profiled across 19 benign, primary malignant and metastatic human prostates (Varambally et al) showing consistent differential expression of Hes6-driven genes amongst metastatic tumors.
