# Supplementary figures and images for: HES6 drives a critical AR transcriptional programme to induce castration-resistant prostate cancer through activation of an E2F1-mediated cell cycle network
Source: EMBO Mol Med. 2014 Apr 14;6(5):651–61. doi: 10.1002/emmm.201303581 (PMC4023887; doi:10.1002/emmm.201303581)

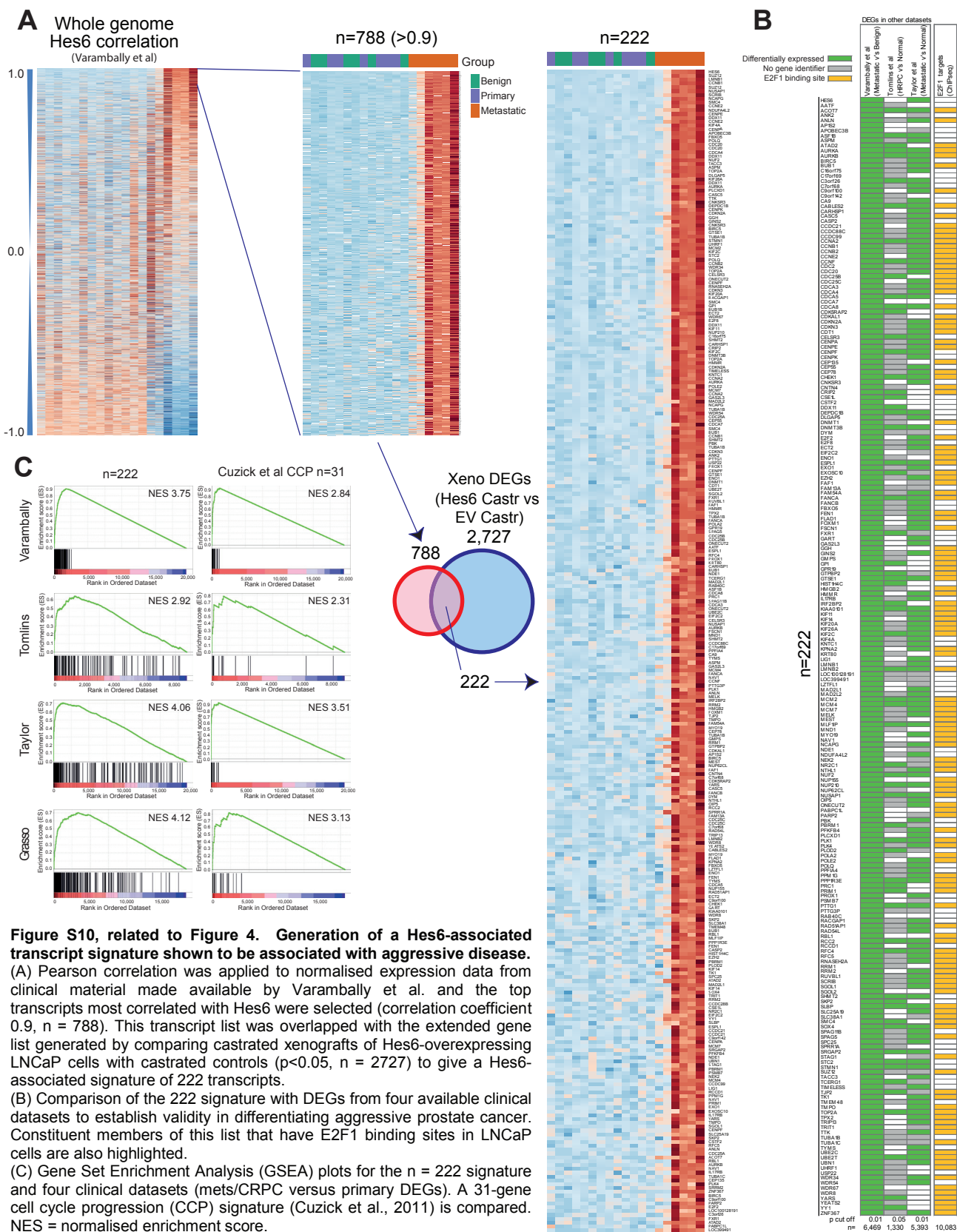

Supplement: Supplementary file 10 [file emmm0006-0651-sd10.pdf]

Figure S2E

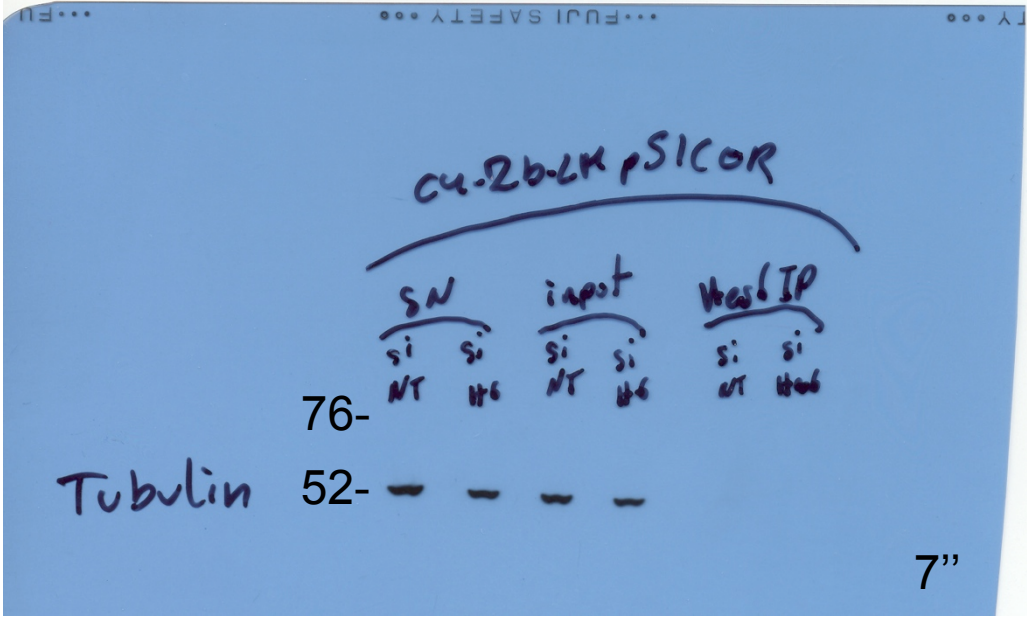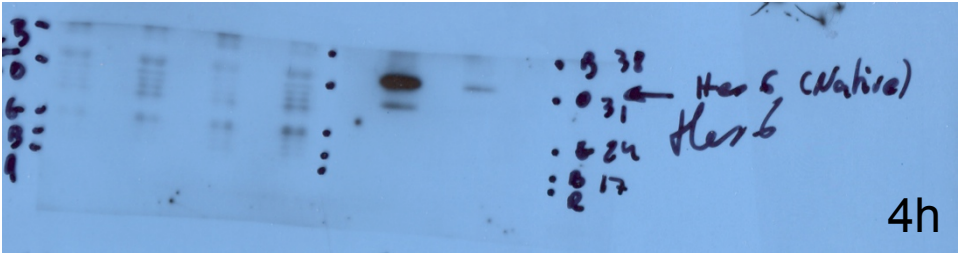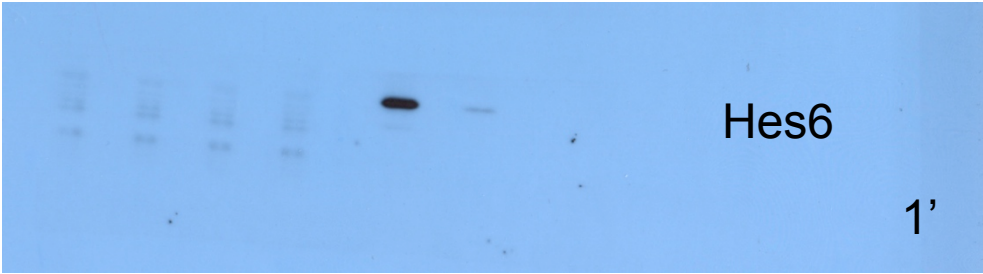

Supplement: Supplementary file 20 [file emmm0006-0651-sd20.pdf]

Figure S4C

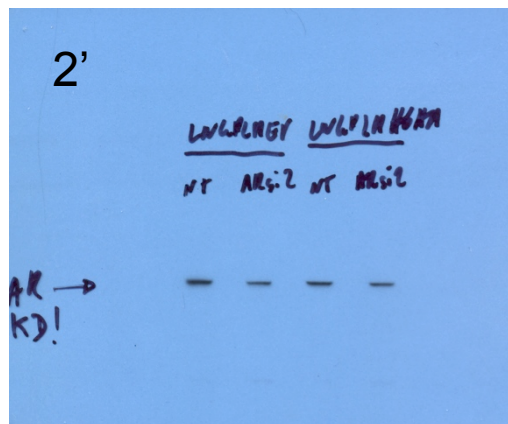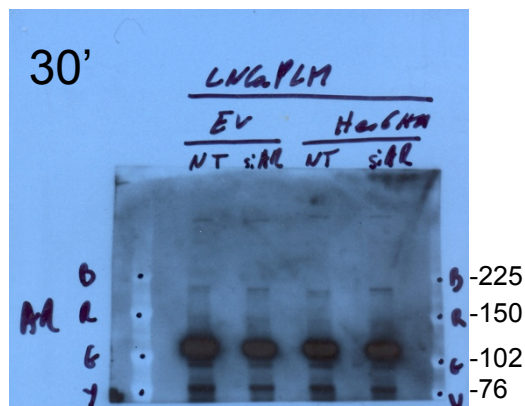

tubulin

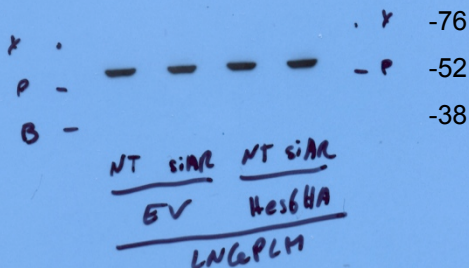

5''

tubulin 2

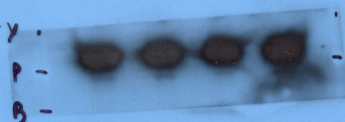

15'

Supplement: Supplementary file 21 [file emmm0006-0651-sd21.pdf]

# Figure 3F

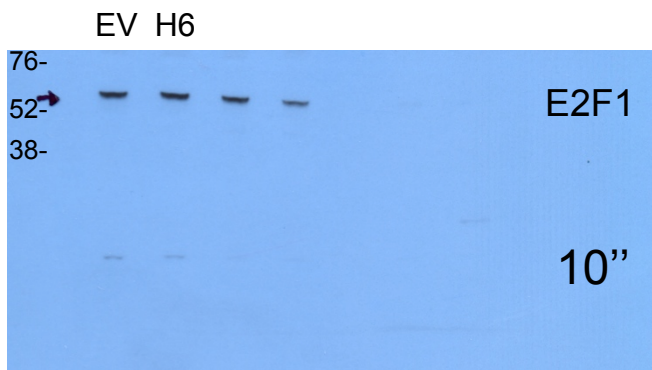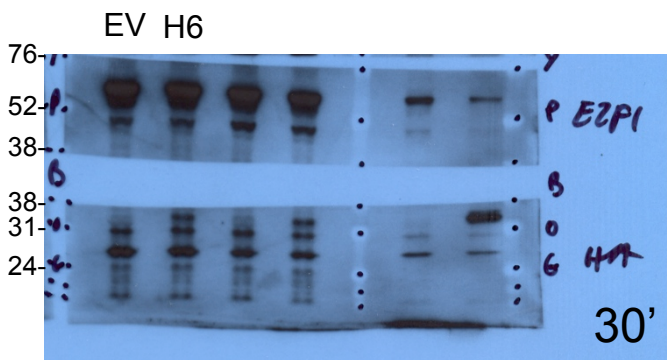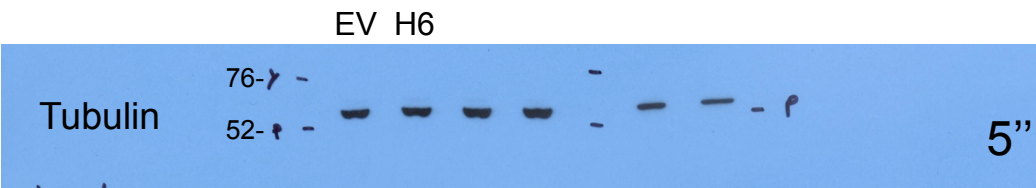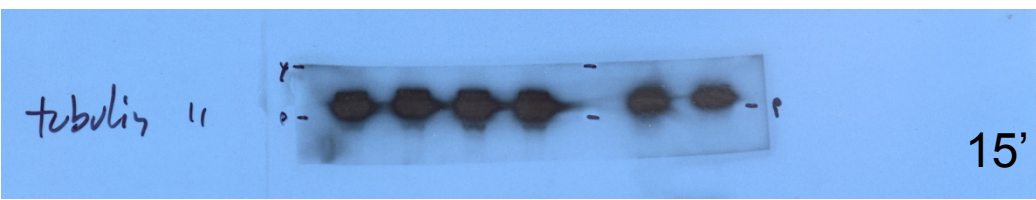

Figure 3G

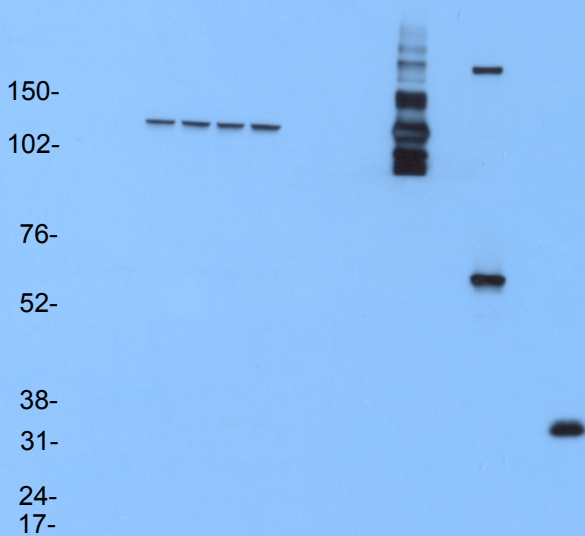

150-

102-

76-

52-

38-

31-

24-

17-

150-

102-

76-

52-

38-

31-

24-

17-

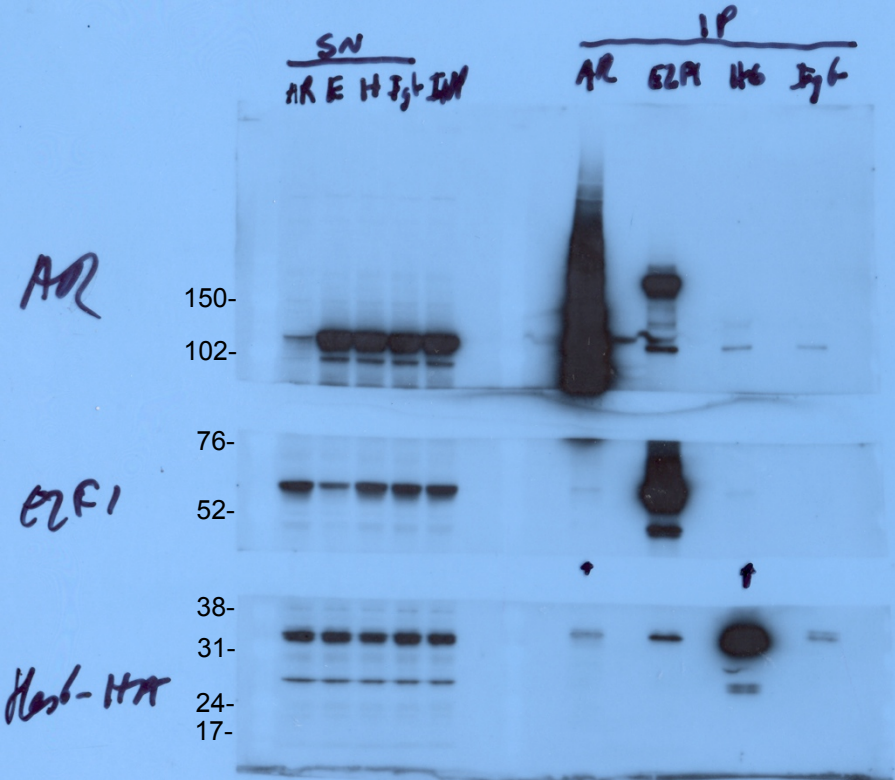

↑  
Hes6-HA  
in E2F1  
IP!!

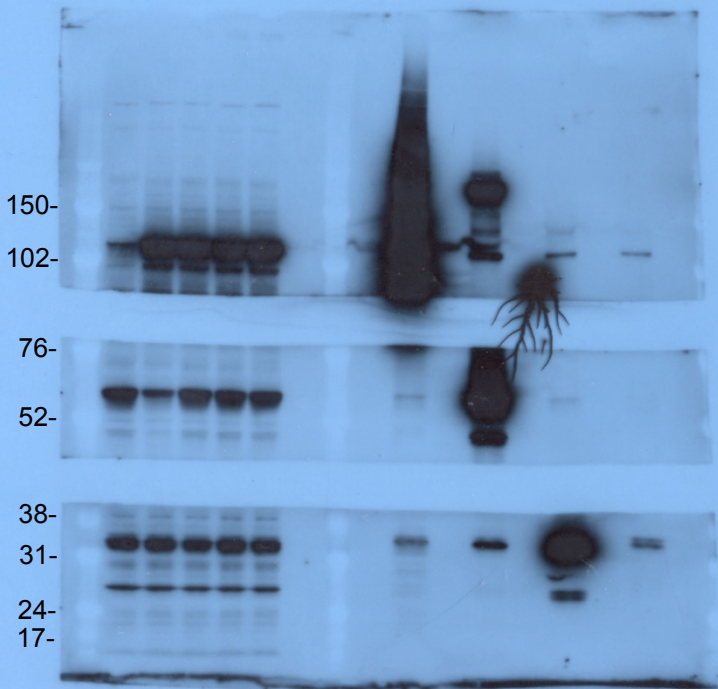

20'

30'

Supplement: Supplementary file 25 [file emmm0006-0651-sd25.pdf]
