# Supplementary material for: HES6 drives a critical AR transcriptional programme to induce castration-resistant prostate cancer through activation of an E2F1-mediated cell cycle network
Source: EMBO Mol Med. 2014 Apr 14;6(5):651–61. doi: 10.1002/emmm.201303581 (PMC4023887; doi:10.1002/emmm.201303581)
Supplement: Supplementary file 11 [file emmm0006-0651-sd11.pdf]

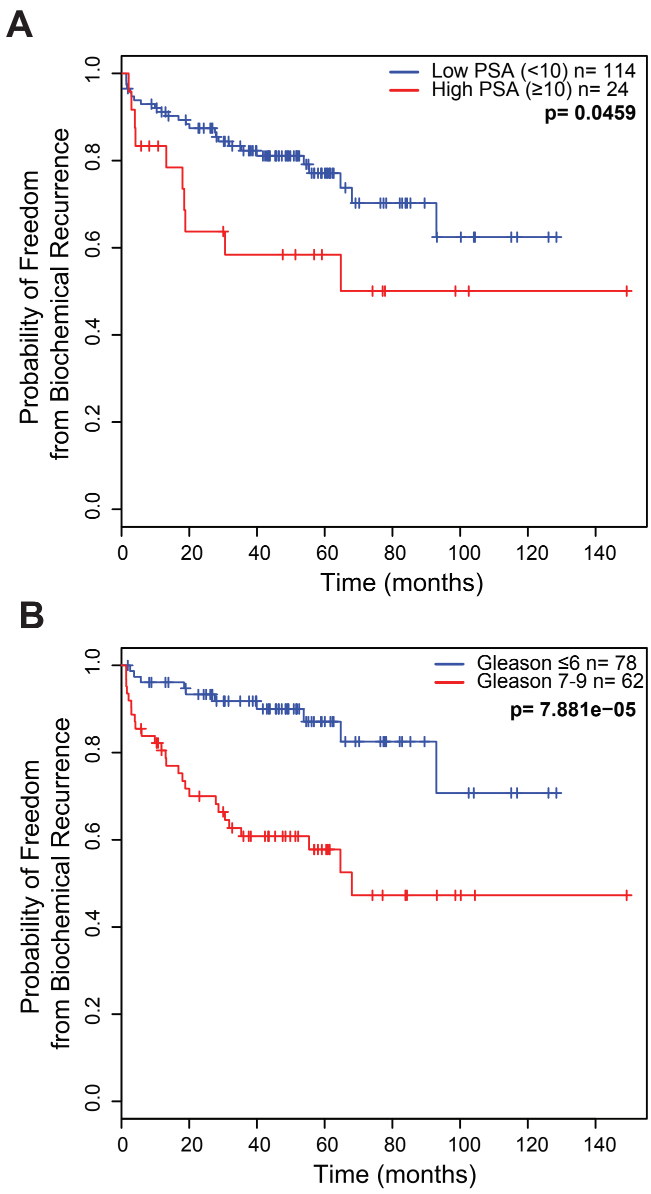

Figure S11, related to Figure 4. Comparative predictive power of Hes6-associated signature, Gleason score and PSA.

(A) Kaplan-Meier survival analysis of post-prostatectomy (Taylor et al) biochemical relapse partitioned by ‘good’ and ‘poor’-risk pre-operative PSA (PSA cut off 10ng/µl; logrank p value 0.0459).

(B) Kaplan-Meier survival analysis of ‘low’ versus ‘intermediate’ and ‘high-risk’ Gleason score (Gleason ≤ 6 versus Gleason >6; logrank p value 7.88e-05).
