# Supplementary material for: HES6 drives a critical AR transcriptional programme to induce castration-resistant prostate cancer through activation of an E2F1-mediated cell cycle network
Source: EMBO Mol Med. 2014 Apr 14;6(5):651–61. doi: 10.1002/emmm.201303581 (PMC4023887; doi:10.1002/emmm.201303581)
Supplement: Supplementary file 12 [file emmm0006-0651-sd12.pdf]

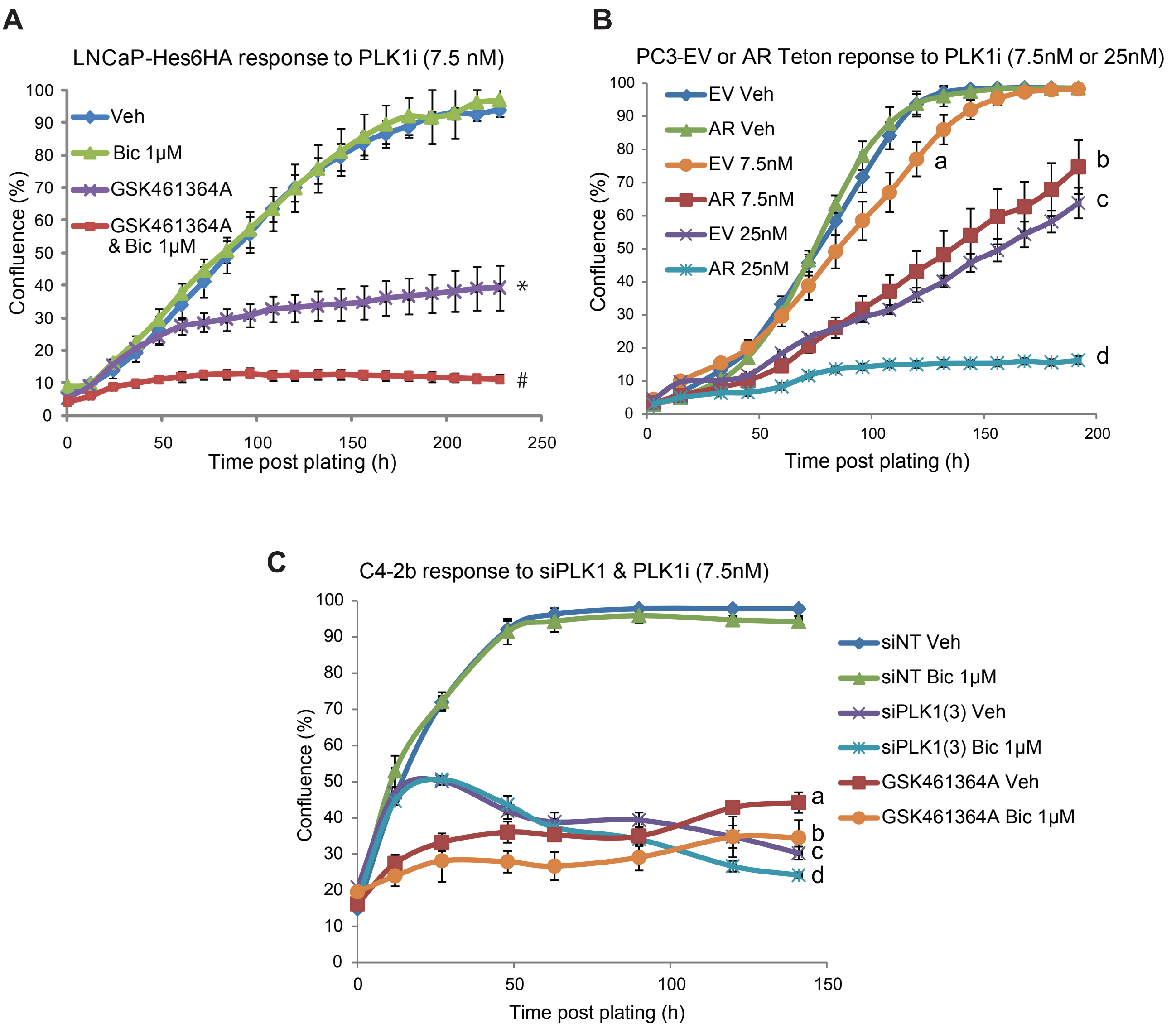

Figure S12, related to Figure 4. PLK1 inhibition reduces androgen-resistant growth.

(A) Selective polo-like kinase 1 (PLK1) inhibitor GSK461364A (7.5nM) attenuates growth of castrate-resistant LNCaP-Hes6HA cells and restores sensitivity to bicalutamide. Vehicle is ETOH, Bic is bicalutamide 1 μM, GSK461364A 7.5 nM; n = 3, error bars represent mean ± SEM; *p = 0.0003, $^{\#}p = 3.3E\text{-}8$ compared to Veh at 225h by t-test.

(B) PLK1 inhibition by GSK461364A (7.5nM) leads to a small reduction in growth of aggressive AR negative PC3 cells, with greater effect at higher dose (25nM). PC3 cells expressing AR have a greater reduction in growth at the same dose. No effect with Bic 1μM. Doxycycline 2μg/ml to all. n = 4, error bars represent mean ± SEM; p = 0.013PLK1i (7.5nM) versus vehicle p<0.05; all other conditions p<0.001 at 120hrs. $^{a}p = 0.013$, $^{b}p = 3.3E\text{-}7$, $^{c}p = 0.004$, $^{d}p = 1.5E\text{-}8$ compared to EV Veh at 120h by t-test.

(C) PLK1 inhibition by both GSK461364A and siRNA reduces growth of castrate resistant cell-line C4-2b with a small synergistic effect with bicalutamide. siPLK1(3) representative of 3 siRNAs. n = 4, error bars represent mean ± SEM; $^{a}p = 4.8E\text{-}7$ compared to siNT Veh, $^{b}p = 0.041$ compared to PLK1i alone, $^{c}p = 2.1E\text{-}10$ compared to siNT Veh, $^{d}p = 0.001$ compared to siPLK1(3) alone at 141h by t-test.
