# Supplementary material for: HES6 drives a critical AR transcriptional programme to induce castration-resistant prostate cancer through activation of an E2F1-mediated cell cycle network
Source: EMBO Mol Med. 2014 Apr 14;6(5):651–61. doi: 10.1002/emmm.201303581 (PMC4023887; doi:10.1002/emmm.201303581)
Supplement: Supplementary file 17 [file emmm0006-0651-sd17.pdf]

SUPPLEMENTARY MATERIALS AND METHODS

Cell Lines, Cell proliferation and Cell Cycle Assays.

LNCaP cells were obtained from ATCC and C4-2 and C4-2b (Thalmann et al, 1994) from MD Anderson Cancer Center, Houston, TX. Luciferase-expressing cell lines LNCaP-LM and C4-2b-LM were generated as previously described (Massie et al, 2011). Cell proliferation was assessed by a comparative field confluence approach (Incucyte, Essen BioScience, Ann Arbor, MI) with statistical significance determined by t-test on mean confluence at the end point of the growth curves shown, unless otherwise indicated. Fluorescence activated cell sorting (FACS) analysis was carried out using a BD LSRII instrument (Becton&Dickinson, San Jose, CA) and data acquisition was performed using BD FACSDiva software.

Culture of Cell Lines

LNCaP, DuCaP (Lee et al, 2001), C4-2 and C4-2b cells were cultured in RPMI with 10% FBS (Full Serum conditions, FS). LNCaP-Myc Tet on cells were cultured in RPMI with 10% FBS + puromycin 2 µg/ml and geneticin 50 µg/ml. LNCaP and DuCaP overexpressing Hes6-HA and C4-2b with a Hes6 knockdown were grown in RPMI with 10% FBS + puromycin 2 µg/ml. LNCaP overexpressing Hes6-HA and with an AR knockdown were grown in RPMI with 10% FBS + puromycin 2 µg/ml + blasticidin 10 µg/ml. Bicalutamide resistant LNCaP cells (LNCaP-Bic) (Hobisch et al, 2006) were

cultured in RPMI with 10% FBS + bicalutamide 1 μM. HEK293TLA (Open Biosystems, Lafayette, CO) cells were maintained in Dulbecco's modified Eagle's medium (DMEM) with 10% FBS, (Invitrogen). Phoenix E and AM12 cells (gift from Michaela Frye) were cultured in DMEM supplemented with L-Glutamine and Pyruvate (Invitrogen). Cells were grown at 37 °C in an atmosphere containing 5% CO_2.

Transduction of Cell Lines.

Lentiviral infection was used to generate the stable cell lines (and their controls) that overexpressed c-Myc or were knockdown for Hes6 or the AR. Retroviral infection was used to generate the Hes6-overexpressing LNCaP and DuCaP cell lines and their EV controls.

Lentiviral infection of cells

HEK293TLA cells were transfected by calcium phosphate. Media was changed after 24 h and fresh media applied. After a further 24 h viral supernatant was filtered (45 μm pore size, Millipore, Billerica, MA), mixed with fresh media and used to infect the cells.

Retroviral infection of cells

Phoenix E cells were transfected by calcium phosphate lysosomal endocytosis at 70-80% confluence in the presence of chloroquine 25 μM. Media was changed after 24 h to remove chloroquine and fresh media applied. After a further 24 h viral supernatant was filtered (45 μm pore size, Millipore, Billerica, MA), mixed with fresh media and 5 μg/ml polybrene and used to infect AM12 cells to increase viral titre. A further infection of the AM12 cells was performed after 24 h. AM12 cells were selected at 60-80% confluence with 2 μg/ml puromycin, media changed after 48 h and the subsequent viral supernatant used to infect cells of interest.

Cell Cycle Assay

Cells were cultured in serum free media for 48 h and then plated at $3x10^5$ cells per well in 6-well plates (9.5 cm^2) in Full Serum conditions. Cells were harvested at intervals, spun 1300 rpm 3’, washed, and fixed while vortexing. From 12 h trypsinisation was required to harvest cells. Prior to this, trypsinisation was not necessary as cells were not yet attached. For DNA content analysis to determine

cell cycle distribution, cells were trypsinized using 0.25% Trypsin-EDTA (Invitrogen), were washed in PBS, resuspended in 80% ice cold methanol and stored at -20 °C until staining. Methanol fixed cells were treated with 3 μM DAPI (Sigma-Aldrich, St. Louis, MO) overnight at 4 °C. Fluorescence activated cell sorting (FACS) analysis was carried out using a BD LSRII instrument (Becton&Dickinson, San Jose, CA) and data acquisition was performed using BD FACSDiva software. The fluorescence emitted by DAPI was collected using a 450/50 bandpass filter. Data were analysed after doublet discrimination using the FlowJo software (Tree Star, Ashland, OR).

Xenograft Implantation and Tumour Formation.

Two million cells were injected subcutaneously in the flank of male NSG mice in a 1:1 mix of PBS and HC-matrigel (BD, Franklin Lakes, NJ). These were allowed to grow for approximately four weeks before castration (target size before treatment was 100 mm^3 calculated by length x height x width x 0.5326 (Andersen et al, 2010). Grafts were monitored weekly by imaging after intraperitoneal injection of D-luciferin 150 mg/kg (Caliper Life Sciences, Hopkinton, MA). Luminescent measurements were analysed using Xenogen Imaging Analysis software "Living Image® 3.0" (Caliper Life Sciences) and plotted as photons/second for graphic analysis of growth kinetics. Mice were culled at completion of experiment or when tumours reached 10% of body weight.

Immunohistochemistry

Slides were stained on a BondMax Autostainer (Leica, Milton Keynes, UK). Antigen retrieval was performed at 100 °C in Bond ER2 diluent or sanger diluent, followed by a 15-min incubation (60 min for NCL-AK2) with primary antibody at room temperature, 8-min incubation using a polymer secondary system (Leica) followed by developing with Diaminobenzidine using copper enhancement. Haematoxylin counterstaining was performed automatically on the Bond system, and finally, the slides were dehydrated, cleared and mounted using a Leica ST5020 attached coverslipper CV5030 (Leica). Antibodies used were as tabled. Slides were scanned onto the Ariol system (Applied Ariol® Imaging, Molecular Devices Ltd) for analysis (AR) or Aperio/Spectrum™ v10.2.2.2317 (Ki67).

Antibodies used in Immunohistochemistry:

Target	Antibody	Manufacturer	Type	Dilution	Retrieval
HsAR	NCL-AR-318	Novocastra	Mm mono	1:50	EDTA 20’
MmAR	sc-816 (N20)	SantaCruz	Rb poly	1:750	EDTA 20’
HsKi67	M7240	Dako	Mm mono	1:200	EDTA 30’
HsPLK1	05-844	Upstate	Mm mono	1:7500	EDTA 20’

Quantitative Assessment of Xenograft IHC

All AR and Ki67 slides were fully digitised on the Leica Ariol SL-50 (Leica Microsystems, Milton Keynes, UK) or the Aperio XT (Aperio, Vista, CA, US), respectively, under x20 magnification. AR analysis was conducted using the Ariol Multistain algorithm following training of the algorithm to recognise positive DAB-stained nuclei and negative Haematoxylin-stained nuclei. Results were expressed as percentage of positive cells following analysis of the entire xenograft. Ki67 was analysed using the Aperio nuclear analysis v9 and splitting individual nuclei into negative, 1+, 2+ and 3+ bins based on the average grey scale of each nucleus, with grey scale values of 255-210 (negative), 210-188 (1+), 188-162 (2+), 162-0 (3+). Results were expressed as stacked bars giving proportionate and numerical splits for each xenograft analysed.

Human Prostate Tissue Samples and HRTMA Construction.

All cases studied attended Addenbrooke’s Hospital (Cambridge, UK) for investigation or treatment of prostate-related disease. Clinical data were identified from hospital records and maintained in a prospectively maintained web-mounted and secured database (https://sbb.nds.ox.ac.uk/camprompt). Prostate was extracted from patients by holmium laser enucleation of the prostate (HoLEP) for benign tissue, robotic assisted laparoscopic prostatectomy (RALP) for primary prostate cancer or channel transurethral resection of the prostate (chTURP) for castrate-resistant disease. Tissue was rapidly fixed in 10% neutral buffered formalin for histology. Sections (5 µm) were cut and stained with hematoxylin and eosin (H&E) to allow confirmation of tissue status by an uropathologist (AW). Immunohistochemistry was performed on neighbouring 5 µm sections. Patients with hormone relapsed prostate cancer were identified as those undergoing any form of androgen-deprivation treatment who

had a sustained rise in PSA from nadir. Patients with clinical progression while on androgen deprivation therapy were also included. All hormone-relapsed tissue was obtained from patients undergoing channel transurethral resection of the prostate (TURP). 45 men were identified from 2001 to 2010 with hormone-relapse disease. Controls included matched benign tissue as well as primary prostate cancer from radical prostatectomy and benign tissue from holmium laser enucleation of the prostate (HoLEP). Formalin fixed paraffin embedded tissue was retrieved from the TURP specimens in the pathology archive. The original haematoxylin and eosin (H&E) stained sections were reviewed by a uropathologist and areas of tumour tissue suitable for inclusion in the TMA were marked on the slides and corresponding paraffin blocks. Cores from these selected areas were manually removed from the donor blocks using a 2 mm skin biopsy punch and incorporated into recipient TMA blocks according to a pre-determined layout, and using pig kidney cores as markers for orientation purposes. For each sample, two tumour cores were incorporated into the TMA, plus a separate core with benign tissue, if present. Immunohistochemistry was performed on 3.5 µm sections from the TMA. An H&E section was reviewed by the uropathologist at the time of assessing the immunohistochemistry, to verify the pathology and suitability of the included tissue cores for scoring.

PLK1 Immunohistochemistry Scoring.

Scoring was performed by two observers, one of whom was a uro-pathologist (AYW), and a consensus obtained. Both observers were blind at initial scoring to the clinical status of the core tissue. Staining was graded with two scores quantifying percentage positive nuclei and intensity. Percentage positive nuclei were scored as: ‘0’ = no positive nuclei; ‘1’ = 1-25%; '2' = 25-50%; '3' = 50-75%; '4' = 75-100%. Intensity was scored as: 0 = no staining; '1' weak; '2' = normal; '3' = strong. The product of the two scores gave a total score for each TMA core which was then matched to the clinical identity.

<u>Western Blotting and Immunoprecipitation</u>

Approximately $20x10^6$ cells per IP were cross-linked with a 1% formaldehyde solution and lysed with 1ml 1% RIPA buffer with added protease inhibitor. 50 µl of washed Dynabeads Protein A (Invitrogen, Grand Island, NY) per reaction were incubated overnight with 10 µg of the appropriate rabbit polyclonal antibody and then washed five times on a magnet with 1% RIPA buffer. Beads were incubated overnight with cell lysate (1 mg) after removal of 100 µl as an input. After further washing

with RIPA buffer and denaturing with NuPAGE (Invitrogen), input, supernatant and IP were run together for resolution by SDS polyacrylamide gel electrophoresis, transferred to nitrocellulose and blotted with appropriate mouse monoclonal antibody.

Antibodies used in IP and ChIP experiments (IP), and in Western Blot (WB):

**IP/ WB**	**Target**	**Code**	**Manufacturer**	**Origin**	**Amount /Conc**
IP	Hes6 (H-180)	sc-25396X	SantaCruz	Rabbit poly	10 µg
IP	AR (N-20)	sc-816	SantaCruz	Rabbit poly	10 µg
IP	E2F1(C-20)	sc-193	SantaCruz	Rabbit poly	10 µg
IP	Myc (N-262)	sc-764	SantaCruz	Rabbit poly	10 µg
IP	Control, normal rabbit IgG	I-1000	Vector	Rabbit poly	10 µg
WB	Hes6 1-15	Gift from	Anders Strom	Rabbit poly	1:1000
WB	HA11	MMS-101P-200	Covance	Mouse mono	1:1000
WB	AR441	M3562	Dako	Mouse mono	1:1000
WB	E2F-1	sc-56661	SantaCruz	Mouse mono	1:1000
WB	Beta-Tubulin	ab11307	Abcam	Mouse mono	1:5000
WB	SYP	ab8049	Abcam	Mouse mono	1:1000
WB	CHGA	C20, sc-1488	SantaCruz	Goat poly	1:1000
WB	AurKA	3092	Cell Signalling	Rabbit poly	1:1000
WB	AurKB	3094	Cell Signalling	Rabbit poly	1:1000
WB	PLK1	ab17057	Abcam	Mouse mono	1:500
WB	Hes1	11988	Cell Signalling	Mouse mono	1:2000
WB	Alpha-Tubulin	ab7291	Abcam	Mouse mono	1:5000

Chromatin Immunoprecipitation (ChIP), Sequencing and Analysis

ChIP, reverse cross-linking and Solexa library prep were carried out as previously published (Schmidt et al, 2008) with some alterations in tissue preparation when working with xenograft material. ChIP-Seq libraries were sequenced on Illumina GAIIx instruments (Illumina, San Diego, CA) and the sequences generated were processed by the Illumina analysis pipeline version 1.6.1, and aligned to the GRCh37 human genome reference build using BWA version 0.5.9 (Li & Durbin, 2009). Alignments were filtered for mapping quality (MapQ >15) and for alignment within the Duke's Excluded Regions (UCSC). Peaks were identified using Macs version 1.41 (Zhang et al, 2008) and consensus peak sets for AR or E2F1 generated by merging all overlapping peaks within AR or E2F1 ChIP-seq experiments using GenomicRanges Bioconductor package (Bioconductor version 2.10). The differing AR peak-set classes were defined using the occurrence of peaks within conditions. 'Enhanced' class was defined by presence of peaks in all replicates of AR-EV-Vehicle, AR-Hes6-Bic, AR-EV-Bic. 'Rescued' was defined by presence of peaks in all replicates of AR-EV-Vehicle, AR-Hes6-Bic but absence in at least 2 out of 3 of AR-EV-Bic replicates. Finally the 'lost' set was defined by presence of peaks in all replicates of AR-EV-Vehicle and absence in 3 out 4 and 2 out of 3 of AR-Hes6-Bic and AR-EV-Bic replicates respectively. To ensure all binding sites within classes would be of significance, sites were further filtered to those showing at least 5-fold enrichment over input. Heatmaps and average profiles of signal across binding sites were calculated using normalised signal derived from read counts. The average summit for a binding site was calculated as the mean location of summits within replicates which contained a peak at that binding site location. Signal was normalised to total reads divided by 1 million (Reads Per Million/RPM). Heatmaps of the normalised signal in a 1000 bp window around summits were generated using Matrix2PNG (version 1.2.1) and average signal plots of these regions produced by custom scripts in R (version 2.15.0).

Where appropriate, overlap, subtraction, union and feature annotation of ChIP-seq enriched regions were performed using Galaxy (Blankenberg et al, 2007; Taylor et al, 2007). Binding sites were integrated with gene expression data using a genomic window of ±25kb.

ChIPseq Co-occurrence

To show co-occurrence of peak sets with other transcription factors, selected transcription factors of interest to this study were retrieved from UCSC (Encode Consortium) and tissue AR ChIPseq sets provided by Sharma et al (Sharma et al, 2013). To score co-occurrence, the base pair overlap between AR binding site classes' peaks and test sets' peaks was identified and this was normalised to the base pairs covered by AR binding site classes' peaks. To further account for the difference in test peak sets, co-occurrence scores were z-transformed within each test set. For visualisation of co-occurrence z-scores, heatmaps were produced using Cluster (Version 3) and TreeView (version 1.6.6).

ChIPseq Genomic Distribution

For the analysis of the relationship between binding sites and gene features, all binding sites were annotated to gene locations retrieved from Ensembl (Ensembl 66: Feb 2012). Transcript models for known protein-coding genes as defined by Ensembl were merged to form representative gene locations using the GenomicRanges package (Bioconductor version 2.10). Pie charts of genomic distribution of binding sites were drawn using R (version 2.15.0) from the distances of binding site centres to the gene start and their occupancy within or outside of a gene.

AR and E2F1 Co-occurrence

The distribution of AR binding site centres to E2F1 binding site centres for the differing AR binding site classes were measured using the Genomic Ranges package (Bioconductor version 2.10). The frequency of AR binding events around E2F1 sites was normalised to the total number of binding events within each AR binding site class and the resulting smoothed distributions were plotted using GGPlot2 R package (version 0.9.3.).

Enrichment and Motif Analysis

To identify enrichment of a gene signature or gene set, the hypergeometric test was used to test a list of DEGs for significant overlap with a gene set or signature, and GSEA (Mootha et al, 2003; Subramanian et al, 2005) was applied to all genes using their respective t-statistics. Motif gene sets from MSigDB C3 motif gene sets (version 3) were used to identify motif enrichment using GSEA. Hypergeometric tests of occupancy of E2F1 within differentially expressed genes was performed using

the phyper function in R (version 2.15.0). For the identification of any known motifs enriched within defined ARBS classes, Ame within the Meme-ChIP suite was used at default settings with Transfac profession (Version 2012.4).

Xenograft Gene Expression

Xenograft RNA Extraction and Microarray Hybridisation

An approximately 3 mm^2 portion of tissue (max. 30 mg) was kept on dry ice and placed in a chilled Mixed Tissue Precellys 24 tube (Bertin Technologies, France) and buzzed at 1x5,000 for 20 seconds in 600 μl of buffer RLT (Qiagen, Hilden, Germany). RNA, DNA and protein were then extracted using AllPrep (RNEasy) extraction kit (Qiagen) as per the manufacturers protocol. RNA was converted to cDNA using High Capacity RNA to cDNA mastermix (Applied Biosystems) for initial quantitative PCR assessment. Total RNA samples were then quality checked and quantified using a Bioanalyser (Agilent Technologies, Santa Clara, CA) and Nanodrop spectrophotometer (Thermo Scientific, Waltham, MA). RNA was diluted to 22.5 ng/μl and 11 μl (250 ng) used for cRNA amplification and labelling with the Illumina TotalPrep-96 Kit (Ambion, Grand Island, NY). Purified, quality controlled and normalised cRNA was hybridized to arrays according to the Illumina protocol (Illumina WGGX DirectHyb Assay Guide 11286331 RevA).

Gene Expression Data

The Illumina BeadChip HumanWG-12 (version 3 and 4; Illumina, San Diego, CA) raw bead-level data were pre-processed using BASH (Cairns et al, 2008), a function from the beadarray package (Dunning et al, 2007) in Bioconductor, and also log base 2 transformed and quantile normalised using the same package. The following raw and normalised publicly available prostate cancer data sets were downloaded from GEO (Gene Expression Omnibus): series GSE3325 (Varambally et al, 2005), series GSE6099 (Tomlins et al, 2005), series GSE21032 (Taylor et al, 2010), series GSE35988 (Grasso et al, 2012). Differential expression analysis was carried out on normalised data using the limma package (Smyth, 2005) from Bioconductor and p-values were adjusted for multiple testing using the Benjamini–Hochberg method (Benjamini & Hochberg, 1995). Lists of statistically significant differentially expressed genes (DEGs) were created using an adjusted p-value threshold of 0.05 or 0.01. The Pearson's correlation coefficient was used to determine genes with similar expression profiles and

hierarchical agglomerative clustering was performed on z-score transformed normalised data using the hclust function in R using Euclidean distance and the complete linkage method. To identify statistically significant biochemical recurrence courses, recursive partitioning (Hothorn et al, 2006) was performed on a single gene expression profile using the 'party' package from CRAN (The_R_Foundation, 2012) with accompanying biochemical recurrence data taken from the Taylor data set (Taylor et al, 2010). Kaplan Meier plots of the risk of biochemical recurrence were produced using the 'survival' package from CRAN and p-values from the log rank test were corrected using the Bonferroni correction method. The Cox proportional Hazards Model was used to calculate Hazard ratios and the association of various clinical factors with the Hes6-associated signature using the rms package from CRAN. The cph and lrtest functions from rms were used to fit the Cox model and to test for prognostic effect.

Pathway Analysis

Biological function and network generation was performed using Ingenuity Pathway Analysis (IPA, Ingenuity Systems, Redwood City, CA). A data set containing gene identifiers was uploaded into the application. Each identifier was mapped to its corresponding molecule in Ingenuity's Knowledge Base. These Network Eligible Molecules were overlaid onto a global molecular network developed from information contained in Ingenuity's Knowledge Base. Networks of Network Eligible Molecules were then algorithmically generated based on their connectivity. The Functional Analysis tool identified the biological functions that were most significant to the data set. Molecules from the dataset that were associated with biological functions in Ingenuity's Knowledge Base were considered for the analysis. Right-tailed Fisher's exact test was used to calculate a p-value determining the probability that each biological function assigned to that data set was due to chance alone.

Real-Time Q-PCR Analysis.

Hes6 and c-Myc transcripts were measured using real-time TaqMan assays (Applied Biosystems, Carlsbad, CA). AR transcripts were analysed using SybrGreen chemistry (Applied Biosystems). These assays were performed in an ABI7900 instrument (Applied Biosystems).

Primers, Probes and Oligonucleotides

Taqman probes used to measure gene expression:

Transcript	Taqman Probe	Manufacturer
HsHes6	HsHes6Hs00610927_g1	Applied Biosystems
HsMyc	HsMycHs00153408_m1	Applied Biosystems
HsTBP	4326322EVic	Applied Biosystems
HsRPLPO	4326314EVic	Applied Biosystems

Primers and oligonucleotides used for cloning, RT-QPCR and generation of knockdowns:

Target	Direction	Sequence	Application
c-Myc	Fwd	GCCTGGAGAAGGATCCCTGGATTTTTTTCG GGTAGTG	Cloning
c-Myc	Rev	GCGCGGCCGCGGATCCTCAAGCGTAATCTG GAACATCGTATGGGTACGCACAAGAGTTCC GTAGCTGTTC	Cloning
Hes6	Fwd	GGGATGGCGCCACCCGCGGCG	Cloning
Hes6	Rev	GGGTCACCAAGGCCTCCAGACACTCCG	Cloning
Hes6	Fwd	TCAGCCTGACCACAGCCCAATTCAAGAGAT TGGGCTGTGGTCAGGCTGTTTTTTC	shRNA oligo
Hes6	Rev	TCGAGAAAAAACAGCCTGACCACAGCCCA ATCTCTTGAATTGGGCTGTGGTCAGGCTGA	shRNA oligo
AR	Fwd	TGCTGAGTATTCCCCTTTCATTCAAGAGATG AAAGGGGAATACTCAGCTTTTTTC	shRNA oligo
AR	Rev	TGCTGAGTATTCCCCTTTCATTCAAGAGATG AAAGGGGAATACTCAGCTTTTTTC	shRNA oligo
AR	Fwd	CTCACCAAGCTCCTGGACTC	Gene Expr
AR	Rev	CAGGCAGAAGACATCTGAAAG	Gene Expr
SDH	Fwd	TGGGAACAAGAGGGCATCTG	Gene Expr
SDH	Rev	CCACCACTGCATCAAATTCATG	Gene Expr
TBP	Fwd	GAATATAATCCCAAGCGGTTTG	Gene Expr

TBP	Rev	ACTTCACATCACAGCTCCCC	Gene Expr
AURKA-E2F1BS	Fwd	GTAACAAGCAGTATCCTACAGGGC	ChIP
AURKA-E2F1BS	Rev	TTCCTCCGTCCCTGAGTGTCCTT	ChIP
AURKB-E2F1BS	Fwd	ATGCCTAGTTCCCATTGGCTCGAT	ChIP
AURKB-E2F1BS	Rev	TCCTAAACTGGAAGCCAAGCGTGA	ChIP
BUB1-E2F1BS	Fwd	GCCAAACCTGAACCGCAAACTAGA	ChIP
BUB1-E2F1BS	Rev	AAGTGGGAGGAGCTACTGGCTCAA	ChIP
CDK4-E2F1BS	Fwd	ATAGACACAGGCCGCAAGCTAGA	ChIP
CDK4-E2F1BS	Rev	TTACACTCTTCGCCCTCCTCCCA	ChIP
CDC2-E2F1BS	Fwd	TCTAGCCGCCCTTTCCTCTTTCTT	ChIP
CDC2-E2F1BS	Rev	TTGAAGCCAAGTGCGAGCAGTTTC	ChIP
CDC20-E2F1BS	Fwd	CTCTCGTGATAGCTGAGACTTTCC	ChIP
CDC20-E2F1BS	Rev	CCTGGCTTACGCCTCTTAAACTCT	ChIP
CENPM-E2F1BS	Fwd	ACTCGCCAATCAGAAGCTGGGAAA	ChIP
CENPM-E2F1BS	Rev	TGCTCTGAACCAATTGGAGTAGCC	ChIP
UBE2C-E2F1BS	Fwd	GATCAAACCCAAGCGAGCCATTGA	ChIP
UBE2C-E2F1BS	Rev	AACTCGGAGAACACGACTGCAACT	ChIP
CONTROL-1-NO-E2F1BS	Fwd	ACAGGTACCACAATGCCTAGCAGA	ChIP
CONTROL-1-NO-E2F1BS	Rev	GTCTCATGTCAAGTTGGTCAGGGT	ChIP
CONTROL-2-NO-E2F1BS	Fwd	ACTTGGAGTTCTGTGGGCCTGAAT	ChIP
CONTROL-2-NO-E2F1BS	Rev	ATATCTTGGGCAGATCCTTGCCGT	ChIP
CAMKK2-ARChIP-1F	Fwd	ACAGGTGGCATGACAGATTTA	ChIP
CAMKK2-ARChIP-1R	Rev	ATCCAGTAACAGGAAAGGACAC	ChIP
KLK3-ARChIP-F	Fwd	GACCTACTCTGGAGGAACATATTG	ChIP
KLK3-ARChIP-R	Rev	GGCTTGCTTACTGTCCTAGATAA	ChIP
KLK15-ARChIP-F	Fwd	GGATCAGCAGTCCGACATAAC	ChIP
KLK15-ARChIP-R	Rev	AGGGAGATGCAAGGAAATCTG	ChIP
KLK2-ARChIP-F	Fwd	GACCTACTCTGGAGGAACATATTG	ChIP
KLK2-ARChIP-R	Rev	CTAGGTTTGCTTACTGCCTTAGA	ChIP

NKX3-1-ARChIP-F	Fwd	GCAAGTTTGCTCAGGAAGTG	ChIP
NKX3-1-ARChIP-R	Rev	AGCCAGAGGGTTGAACTTATC	ChIP
GDF15-ARChIP-F	Fwd	GTGTGCCTGAAAGATTTCACAAT	ChIP
GDF15-ARChIP-R	Rev	GTGGAATTACTGGGTCACAGAG	ChIP
HIF1A-ARChIP-F	Fwd	AGTAGGTCTCTTCTCTCTTGATTTG	ChIP
HIF1A-ARChIP-R	Rev	TGGCTGGCATGTACTGAATAA	ChIP
RFC1-ARChIP-F	Fwd	GTCCAGTTTCCCAACAAACAAA	ChIP
RFC1-ARChIP-R	Rev	ACTTAAGTGTTTACTCTGTGCCT	ChIP
SPRY1-ARChIP-F	Fwd	GCTTTAGTGTCTTCCTGGATAGG	ChIP
SPRY1-ARChIP-R	Rev	GCTCACCCTCTAATCCAACAG	ChIP
TLE4-ARChIP-F	Fwd	AGGGAGCTTAGGCATGTTTG	ChIP
TLE4-ARChIP-R	Rev	TCCCAGCACAATTTGCAGTA	ChIP
TMPRSS2-ARChIP-F	Fwd	AGTACCTGCCGTACCCTTTA	ChIP
TMPRSS2-ARChIP-R	Rev	TGTAATTCTGGAAGCTGACCTTTA	ChIP
TP63-ARChIP-F	Fwd	CTCCAACCTAGTCAAATTACTCCA	ChIP
TP63-ARChIP-R	Rev	GTTCCAAACCAAGAAACCATACC	ChIP
HES6-ARChIP-F1	Fwd	CACCTGTGTAAGCACACACACTA	ChIP
HES6-ARChIP-R1	Rev	CGCGTGCCAAGCAGTTGACTTAAT	ChIP
FKBP5-ARChIP-F	Fwd	CCCCCCTATTTTAATCGGAGTAC	ChIP
FKBP5-ARChIP-R	Rev	TTTTGAAGAGCACAGAACACCCT	ChIP
CONTROL-NOARBS-1F	Fwd	AGGTTGTCCTCATGACCTTCAGCA	ChIP
CONTROL-NOARBS-1R	Rev	AGGTCAGCAGGTGTCAGTGAGAAA	ChIP
CONTROL-NOARBS-2F	Fwd	ACAGGTACCACAATGCCTAGCAGA	ChIP
CONTROL-NOARBS-2R	Rev	GTCTCATGTCAAGTTGGTCAGGGT	ChIP
CONTROL-NOARBS-3F	Fwd	AGCTGTGGAAGCTTGTGGTCTTCT	ChIP
CONTROL-NOARBS-3R	Rev	GAACCAGGCTGTTCTGTGCAGTTT	ChIP
