# Supplementary material for: HES6 drives a critical AR transcriptional programme to induce castration-resistant prostate cancer through activation of an E2F1-mediated cell cycle network
Source: EMBO Mol Med. 2014 Apr 14;6(5):651–61. doi: 10.1002/emmm.201303581 (PMC4023887; doi:10.1002/emmm.201303581)
Supplement: Supplementary file 18 [file emmm0006-0651-sd18.pdf]

Table S1, related to Fig 2. AR ChIPseq binding sites grouped by 'enhanced', 'rescued' or 'lost' with Hes6 overexpression on AR inhibition.

Table S2, related to Fig 2. MEME motif enrichment analysis comparing the top *m aintained* ('*enhanced'* + '*rescued'*) and '*lost'* ARBS with Hes6 overexpression on AR inhibition compared to a random ARBS set.

Table S3, related to Fig 3. GSEA motif enrichment amongst Hes6 versus EV castrated LNCaP-LM xenograft DEGs and Hes6 versus EV LNCaP cells. ES = Enrichment score. NES = normalised enrichment score. FDR = false discovery rate.

Table S4, related to Fig 4. Hes6-associated gene signature (n=222).
