# Supplementary material for: HES6 drives a critical AR transcriptional programme to induce castration-resistant prostate cancer through activation of an E2F1-mediated cell cycle network
Source: EMBO Mol Med. 2014 Apr 14;6(5):651–61. doi: 10.1002/emmm.201303581 (PMC4023887; doi:10.1002/emmm.201303581)
Supplement: Supplementary file 19 [file emmm0006-0651-sd19.pdf]

SUPPLEMENTARY REFERENCES

Andersen RJ, Mawji NR, Wang J, Wang G, Haile S, Myung JK, Watt K, Tam T, Yang YC, Banuelos CA et al (2010) Regression of castrate-recurrent prostate cancer by a small-molecule inhibitor of the amino-terminus domain of the androgen receptor. Cancer Cell 17**:** 535-546

Benjamini Y, Hochberg Y (1995) Controlling the false discovery rate: a practical and powerful approach to multiple testing. J Roy Statist Soc Ser B (Methodological) 57**:** 289-300

Blankenberg D, Taylor J, Schenck I, He J, Zhang Y, Ghent M, Veeraraghavan N, Albert I, Miller W, Makova KD et al (2007) A framework for collaborative analysis of ENCODE data: making large-scale analyses biologist-friendly. Genome Res 17**:** 960-964

Cairns JM, Dunning MJ, Ritchie ME, Russell R, Lynch AG (2008) BASH: a tool for managing BeadArray spatial artefacts. Bioinformatics 24**:** 2921-2922

Dunning MJ, Smith ML, Ritchie ME, Tavare S (2007) beadarray: R classes and methods for Illumina bead-based data. Bioinformatics 23**:** 2183-2184

Grasso CS, Wu YM, Robinson DR, Cao X, Dhanasekaran SM, Khan AP, Quist MJ, Jing X, Lonigro RJ, Brenner JC et al (2012) The mutational landscape of lethal castration-resistant prostate cancer. Nature 487**:** 239-243

Hobisch A, Fritzer A, Comuzzi B, Fiechtl M, Malinowska K, Steiner H, Bartsch G, Culig Z (2006) The androgen receptor pathway is by-passed in prostate cancer cells generated after prolonged treatment with bicalutamide. Prostate 66**:** 413-420

Hothorn T, Hornik K, Zeileis A (2006) Unbiased recursive partitioning: A conditional inference framework. J Comput Graph Stat 15**:** 651-674

Lee YG, Korenchuk S, Lehr J, Whitney S, Vessela R, Pienta KJ (2001) Establishment and characterization of a new human prostatic cancer cell line: DuCaP. In Vivo 15**:** 157-162

Li H, Durbin R (2009) Fast and accurate short read alignment with Burrows-Wheeler transform. Bioinformatics 25**:** 1754-1760

Massie CE, Lynch A, Ramos-Montoya A, Boren J, Stark R, Fazli L, Warren A, Scott H, Madhu B, Sharma N et al (2011) The androgen receptor fuels prostate cancer by regulating central metabolism and biosynthesis. EMBO J 30**:** 2719-2733

Mootha VK, Lindgren CM, Eriksson KF, Subramanian A, Sihag S, Lehar J, Puigserver P, Carlsson E, Ridderstrale M, Laurila E et al (2003) PGC-1alpha-responsive genes involved in oxidative phosphorylation are coordinately downregulated in human diabetes. Nat Genet 34**:** 267-273

Schmidt D, Stark R, Wilson MD, Brown GD, Odom DT (2008) Genome-scale validation of deep-sequencing libraries. PLoS One 3**:** e3713

Sharma NL, Massie CE, Ramos-Montoya A, Zecchini V, Scott HE, Lamb AD, MacArthur S, Stark R, Warren AY, Mills IG et al (2013) The androgen receptor induces a distinct transcriptional program in castration-resistant prostate cancer in man. Cancer Cell 23**:** 35-47

Smyth GK (2005) Limma: linear models for microarray data. In Bioinformatics and Computational Biology Solutions using R and Bioconductor, Gentleman R, Carey V, Huber W, Irizarry R, Dudoit S (eds) pp 397–420. New York: Springer

Subramanian A, Tamayo P, Mootha VK, Mukherjee S, Ebert BL, Gillette MA, Paulovich A, Pomeroy SL, Golub TR, Lander ES et al (2005) Gene set enrichment analysis: a knowledge-based approach for interpreting genome-wide expression profiles. Proc Natl Acad Sci U S A 102**:** 15545-15550

Taylor BS, Schultz N, Hieronymus H, Gopalan A, Xiao Y, Carver BS, Arora VK, Kaushik P, Cerami E, Reva B et al (2010) Integrative genomic profiling of human prostate cancer. Cancer Cell 18**:** 11-22

Taylor J, Schenck I, Blankenberg D, Nekrutenko A (2007) Using galaxy to perform large-scale interactive data analyses. Curr Protoc Bioinformatics Chapter 10**:** Unit 10 15

Thalmann GN, Anezinis PE, Chang SM, Zhau HE, Kim EE, Hopwood VL, Pathak S, von Eschenbach AC, Chung LW (1994) Androgen-independent cancer progression and bone metastasis in the LNCaP model of human prostate cancer. Cancer Res 54**:** 2577-2581

The_R_Foundation (2012) The Comprehensive R Archive Network. In The R Project for Statistical Computing.

Tomlins SA, Rhodes DR, Perner S, Dhanasekaran SM, Mehra R, Sun XW, Varambally S, Cao X, Tchinda J, Kuefer R et al (2005) Recurrent fusion of TMPRSS2 and ETS transcription factor genes in prostate cancer. Science 310**:** 644-648

Varambally S, Yu J, Laxman B, Rhodes DR, Mehra R, Tomlins SA, Shah RB, Chandran U, Monzon FA, Becich MJ et al (2005) Integrative genomic and proteomic analysis of prostate cancer reveals signatures of metastatic progression. Cancer Cell 8**:** 393-406

Zhang Y, Liu T, Meyer CA, Eeckhoute J, Johnson DS, Bernstein BE, Nusbaum C, Myers RM, Brown M, Li W et al (2008) Model-based analysis of ChIP-Seq (MACS). Genome Biol 9**:** R137
