# Supplementary material for: HES6 drives a critical AR transcriptional programme to induce castration-resistant prostate cancer through activation of an E2F1-mediated cell cycle network
Source: EMBO Mol Med. 2014 Apr 14;6(5):651–61. doi: 10.1002/emmm.201303581 (PMC4023887; doi:10.1002/emmm.201303581)
Supplement: Supplementary file 22 [file emmm0006-0651-sd22.pdf]

Figure S5A

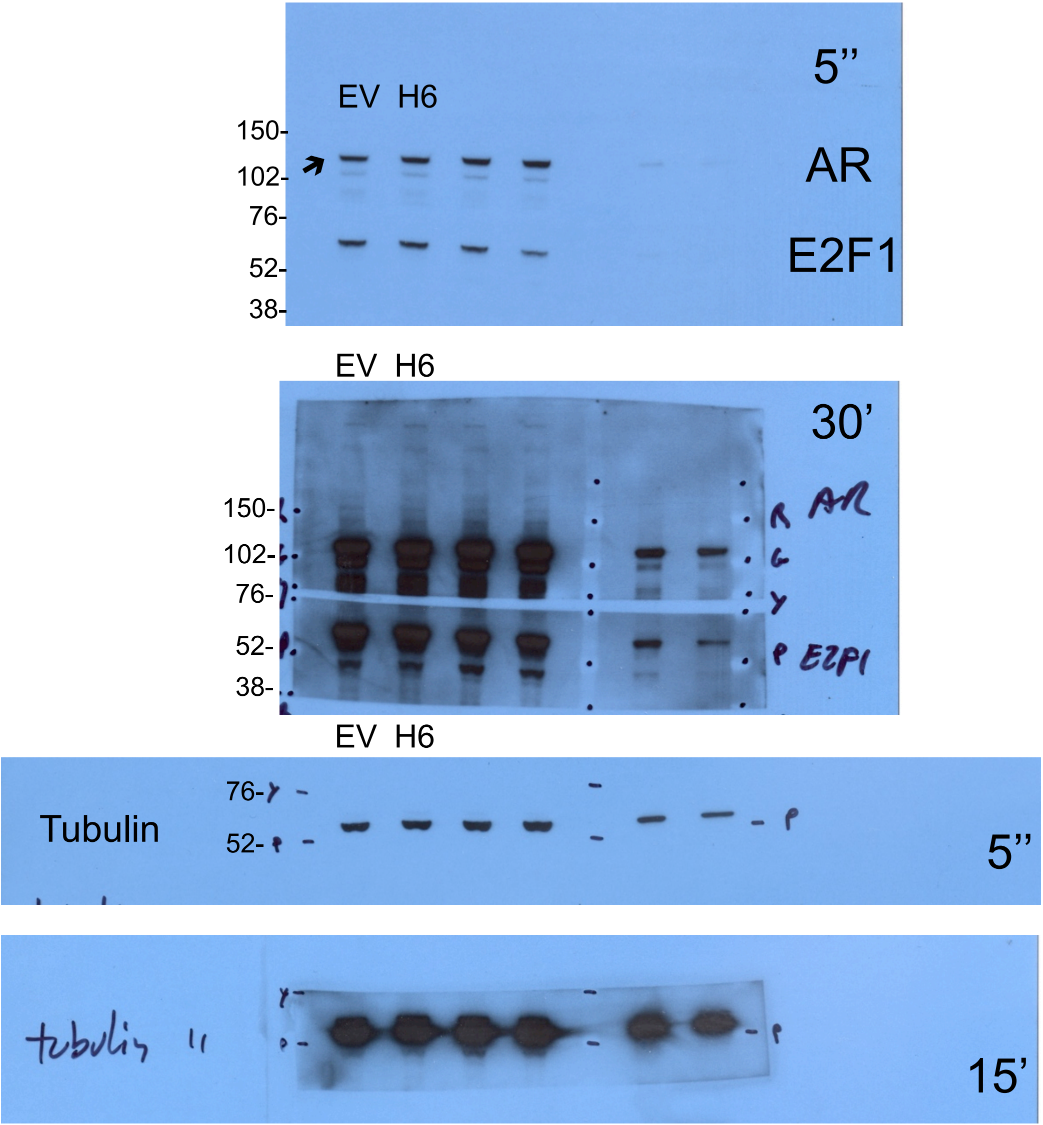

5''
EV H6
150-
102-
76-
52-
38-
AR
E2F1
EV H6
30'
150-
102-
76-
52-
38-
AR
E2F1
EV H6
76-
52-
Tubulin
5''
tubulin
15'

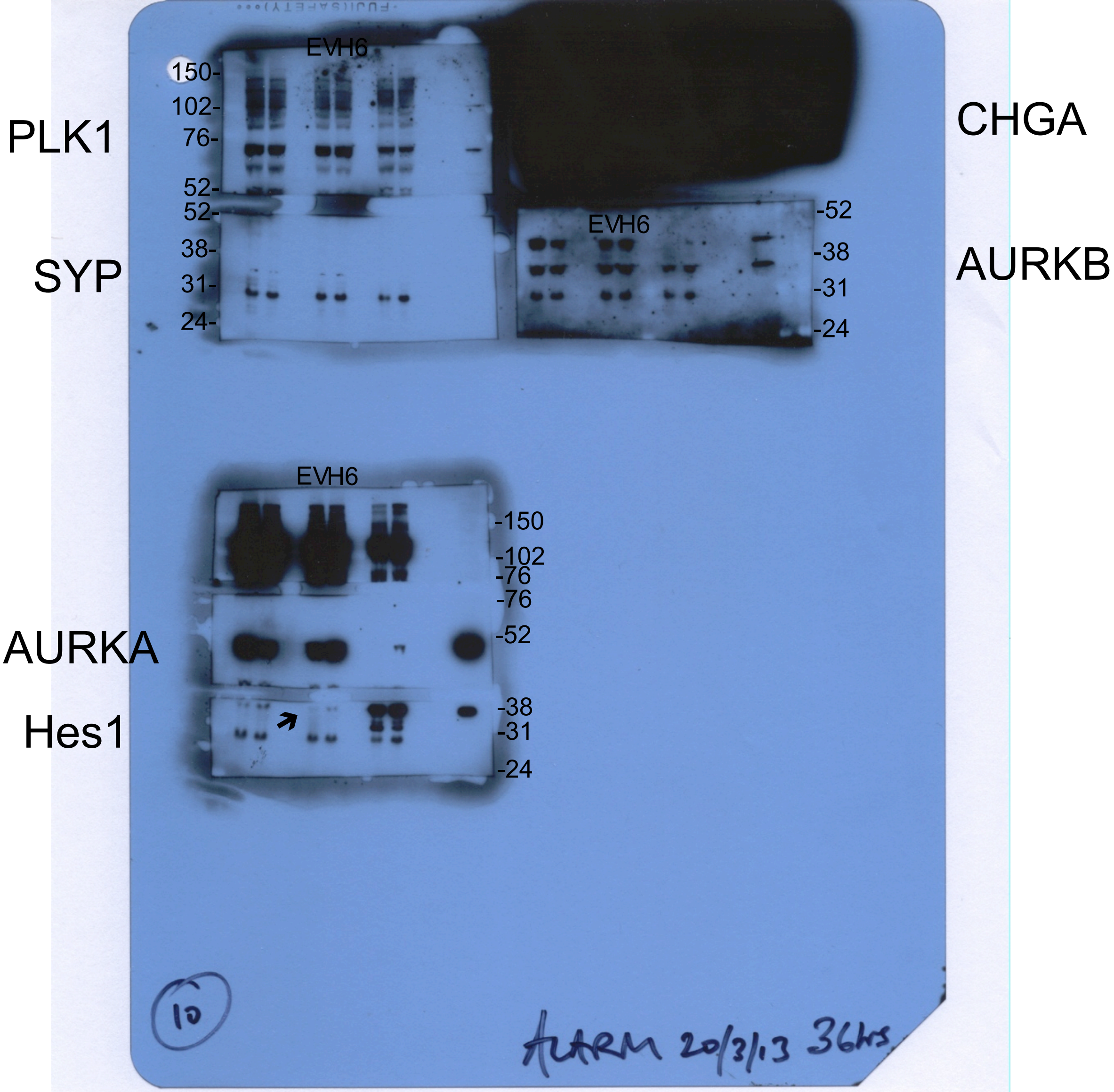
PLK1
SYP
CHGA
AURKB
AURKA
Hes1
EVH6
EVH6
EVH6
150-
102-
76-
52-
52-
38-
31-
24-
-52
-38
-31
-24
-150
-102
-76
-76
-52
-38
-31
-24
10
20/3/13 36hrs

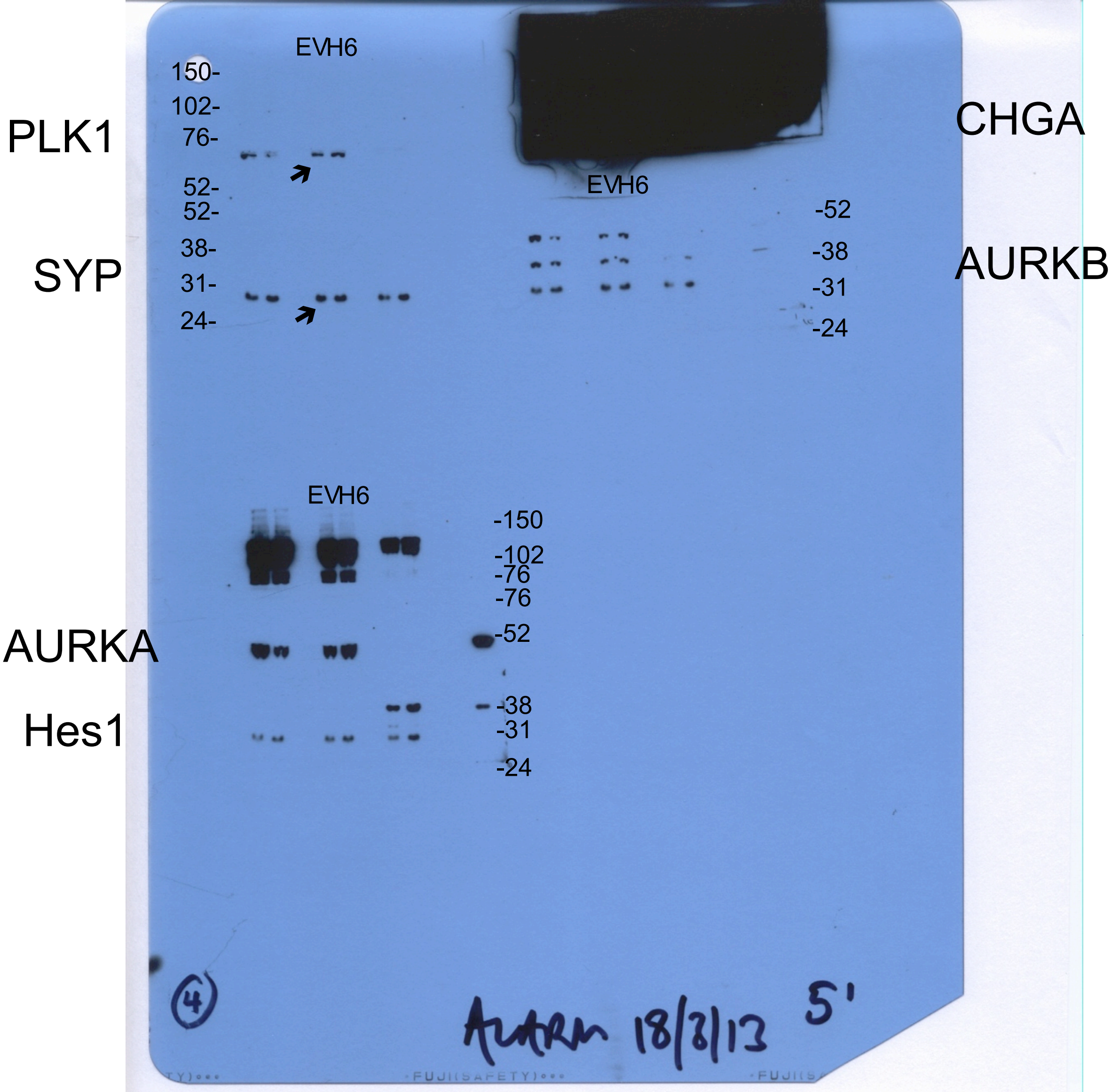

EVH6
150-
102-
76-
52-
52-
38-
31-
24-
PLK1
SYP
CHGA
EVH6
-52
-38
-31
-24
AURKB
EVH6
-150
-102
-76
-76
-52
-38
-31
-24
AURKA
Hes1
4
18/8/13
5'
FUJI(SAFETY)

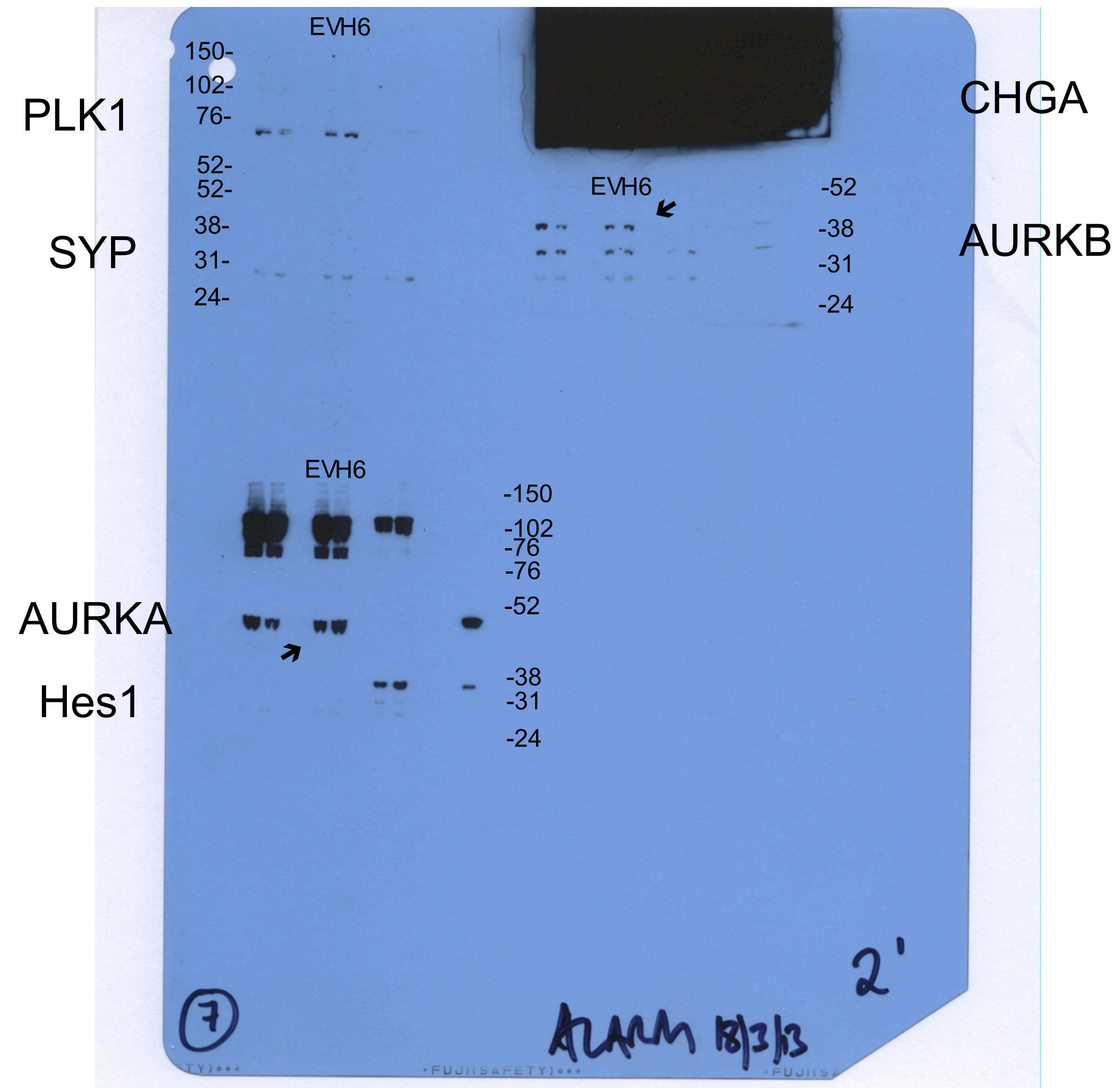
EVH6
150-
102-
76-
52-
52-
38-
31-
24-
PLK1
SYP
CHGA
EVH6
-52
-38
-31
-24
AURKB
EVH6
-150
-102
-76
-76
-52
-38
-31
-24
AURKA
Hes1
2'
18/3/13
FUJI(SAFETY)

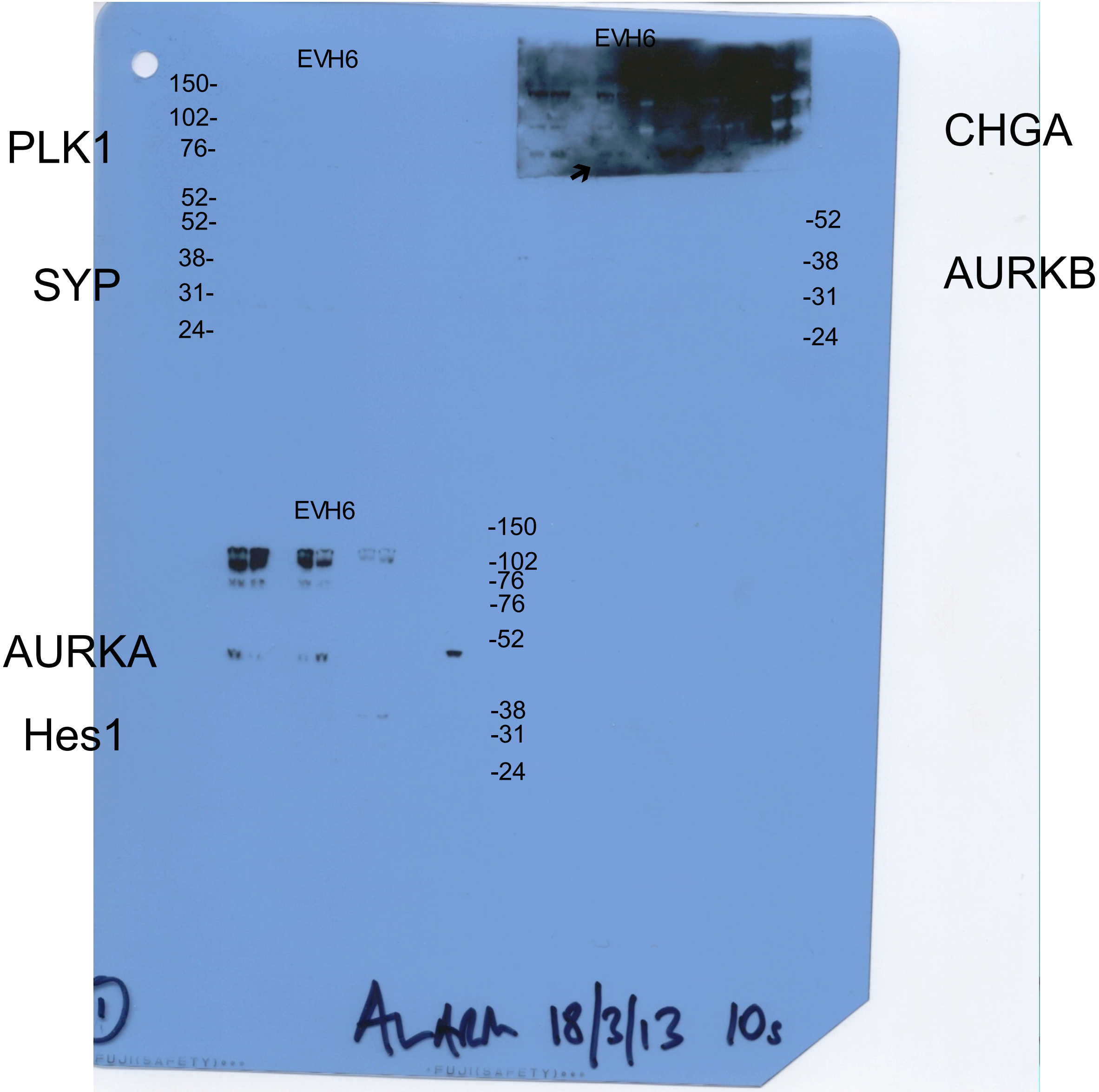

EVH6
150-
102-
PLK1
76-
52-
52-
SYP
38-
31-
24-
EVH6
CHGA
-52
-38
AURKB
-31
-24
EVH6
-150
-102
-76
-76
AURKA
-52
-38
Hes1
-31
-24
FUJI(SAFETY)
FUJI(SAFETY)
AHRM 18/3/13 10s

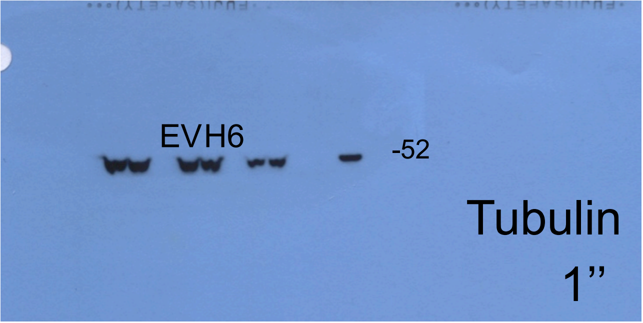
EVH6
-52
Tubulin
1’’
