# Supplementary material for: HES6 drives a critical AR transcriptional programme to induce castration-resistant prostate cancer through activation of an E2F1-mediated cell cycle network
Source: EMBO Mol Med. 2014 Apr 14;6(5):651–61. doi: 10.1002/emmm.201303581 (PMC4023887; doi:10.1002/emmm.201303581)
Supplement: Supplementary file 23 [file emmm0006-0651-sd23.pdf]

Hes6 drives a critical AR transcriptional program to induce castration resistant prostate cancer through activation of an E2F1-mediated cell cycle network

Antonio Ramos-Montoya, Alastair D. Lamb, Roslin Russell, Thomas Carroll, Sarah Jurmeister, Nuria Galeano-Dalmau, Charles E. Massie, Joan Boren, Helene Bon, Vasiliki Theodorou, Maria Vias, Gregory L. Shaw, Naomi L. Sharma, Helen Ross-Adams, Helen E. Scott, Sarah L. Vowler, William J. Howat, Anne Y. Warren, Richard F. Wooster, Ian G. Mills and David E. Neal

Corresponding author: Alastair D. Lamb, Cancer Research UK

Review timeline:

Submission date:	16 October 2013
Editorial Decision:	16 October 2013
Revision received:	04 February 2014
Editorial Decision:	21 February 2014
Revision received:	06 March 2014
Accepted:	10 March 2014

Transaction Report:

(Note: With the exception of the correction of typographical or spelling errors that could be a source of ambiguity, letters and reports are not edited. The original formatting of letters and referee reports may not be reflected in this compilation.)

Editor: Roberto Buccione

1st Editorial Decision 16 October 2013

Thank you for the submission of your manuscript to EMBO Molecular Medicine. We have now heard back from the three Reviewers whom we asked to evaluate your manuscript.
You will see that while the Reviewers are globally supportive of your work, they express a number of concerns that prevent us from considering publication at this time. I will not dwell into much detail, as the evaluations are detailed and self-explanatory and I will just mention a few main points.

Reviewer 1 suggests that the experiments showing xenograft growth attenuation with constitutive knockdown of Hes6 should be done in a castrated setting. S/he also notes that the effects of Hes6 on the cell cycle are modest and would not necessarily explain the differences in tumour growth. Reviewer also lists other issues that require your action.

Reviewer 2 has only one but significant concern. S/he requires more decisive proof to the effect that response to the polo-like kinase inhibitor requires androgen receptor (AR) signalling and suggests further experimentation, for instance with a non AR-expressing cell line.

Reviewer 3 points to a number of issues that pertain to conclusiveness and interpretation. One issue is, again, that of the relevant model in the context of your conclusions and wonders whether the results are more relevant for neuroendocrine PCa rather than castration-resistant PCa. This Reviewer, as does Reviewer 1, also mentions the experiments illustrated in Fig. 1E and asks if they were performed in a castration setting. Clearly, there is some confusion arising from your

presentation of the data that requires some adjusting. Reviewer 3 also lists several other items that require your attention and action.

Considered all the above, while publication of the paper cannot be considered at this stage, we would be prepared to consider a substantially revised submission, with the understanding that the Reviewers' concerns must be fully addressed, with additional experimental data where appropriate and that acceptance of the manuscript will entail a second round of review.

Please note that it is EMBO Molecular Medicine policy to allow a single round of revision only and that, therefore, acceptance or rejection of the manuscript will depend on the completeness of your responses included in the next, final version of the manuscript.

As you know, EMBO Molecular Medicine has a "scooping protection" policy, whereby similar findings that are published by others during review or revision are not a criterion for rejection. However, I do ask you to get in touch with us after three months if you have not completed your revision, to update us on the status. Please also contact us as soon as possible if similar work is published elsewhere.

I look forward to seeing a revised form of your manuscript as soon as possible.

***** Reviewer's comments *****

Referee #1 (Remarks):

The manuscript by Ramos-Montoya et al. investigates the impact of Hes6, which is over-expressed in castration resistant / metastatic prostate cancer, on the androgen receptor (AR) transcriptome and therapeutic response in prostate cancer cells. Major findings are that:
1. Hes6, which is itself up-regulated by AR or c-myc, modifies ligand-independent AR chromatin binding and regulation of gene expression shifting the pattern towards an E2F1-dominated profile.
2. Hes6 promotes resistance to castration or the AR antagonist bicalutamide.
3. The Hes6-modified AR transcriptome is associated with aggressive prostate cancer and predicts biochemical recurrence.
4. A PLK1 inhibitor restores therapeutic responsiveness to Hes6 overexpressing cells.

On balance, this is a convincing and significant study. However, there are several misleading or vague statements and/or interpretations of data as well as some inconsistencies in the experiments, which need to be addressed.

Specific comments:
1. In Fig. 1B, it is not clear what comparisons are being made and which of the groups are significantly different.
2. Hes6 overexpression does not affect tumor xenograft growth when androgen is present (intact mice, Fig. 1C). Fig. 1E is conducted in intact mice but should be done in castrated mice to determine whether Hes6 knock down blocks castration-resistant growth of C4-2b tumors.
3. Does Hes6 knock down block Myc-mediated cell growth in the presence of bicalutamide?
4. Supplementary Fig. S2A does not show significant differences between LNCaP and the bicalutamide resistant derivative with respect to Hes6 mRNA levels. The statement in text is misleading and needs to reflect the lack of a significant difference between these cells.
5. The authors should explain more clearly how data in Figs. S2C and D were analyzed.
6. The images in Fig. S3A are inadequate for demonstrating sub-cellular localization of AR. The repeated claim that Hes6 drives increased nuclear localization should be removed or additional data / experiments need to be shown.
7. Does Hes6 interact with AR?
8. The effects of Hes6 on cell cycle (Fig. S8B) are very modest. It is unclear whether such effects can account for the differences observed in in vivo tumor growth. This needs to be further addressed experimentally.
9. The statistical analysis of Fig. 4G is unclear. At which time points are there significant differences?
10. What is the mechanistic relationship between Hes6 and PLK1 in prostate cancer?

Minor comments:
1. In Significance section, the comment "...the physical interaction between the AR and Hes6 with E2F1..." requires clarification as it seems to infer that a ternary complex is formed.
2. In 1st part of the Results section, the phrase "...overexpression of c-Myc rescues bicalutamide induced AR antagonism" is confusing and should be restated.
3. Why is there a discrepancy in the extent of R1881-induced up regulation of Hes6 mRNA in LNCaP cells comparing Fig. 1A (approx. 3-fold) vs. S1B (less than 2-fold)?

Referee #2 (Comments on Novelty/Model System):

The authors need to test the growth response of an AR non expressing prostate cancer line (e.g., PC-3) to the PLK-i in order to document that such response actually requires AR signaling. Without this data, no definitive conclusion are possible as to the relationship between PLK inhibition as a readout of AR/HES6 interaction.

Referee #2 (Remarks):

There are only a few suggestions for improvement. First, while bioluminescence is needed for orthotopic tumor evaluation, when tumors are growing subcutaneously in the flank, micro-caliper measurements are more accurate and should be used. This is particularly relevent with AR expressing tumors because AR signaling effects tumor angiogenesis and changes in tumor vascularity effect delivery of luciferin. Thus, bioluminescence is not simply a relection of tumor size but also tumor perfusion.Thus , real tumor volumes should be reported in Fig 1F, 1E, and Fi 4G.

Referee #3 (Comments on Novelty/Model System):

The authors used a sophisticated tools to interrogate he role of Hes6 in prostate cancer using ChIP assays, however they didn't clarify the rational of choosing Hes6. Moreover it was surprising that thy didn't investigate or discuss the potential role in neuroendocrine prostate cancer since it's known that Hes6 play a role in stem cells neurogenesis.
This manuscript could be with big interest if it was explored in the context of neuroendocrine prostate cancer.

Referee #3 (Remarks):

In this manuscript, the authors explored the role of Hes6 in prostate cancer and showed that Hes6 is up-regulated in aggressive phenotype and drives castrate resistant prostate cancer in ligand independent manner. They found that Hes6 maintains AR binding to cell cycle regulatory genes under the control of E2F1. They went further and analyzed Hes6 regulome and found that PLK1 could be a potential target for prostate cancer. Hes6 is a transcription co-factor, previously described as a regulator of stem cells during neurogenesis and as a marker for neuroendocrine prostate cancer. The manuscript describes novel findings however, some data are confusing and others requires more depth and supporting information. The conclusion of the manuscript is overstated.
In neuroendocrine prostate cancer, androgen receptor (AR) is lost and AR dependent genes are inhibited, one will argue that a neuroendocrine marker should be negatively regulated by androgen however the authors showed that androgens regulate positively Hes6 and bicalutamide decreases it. They went and showed that in myc expressing cells, Hes6 is increased with bicalutamide. The reviewer is confused and having hard time to understand the opposite data. However it's clear that myc cooperate with AR inactivation to enhance Hes6 expression.
1. LNCaP cells harbor a mutation in AR making them resistant to bicalutamide, does targeting AR with Enzalutamide regulates Hes6 expression
2. The authors should show the expression of Hes6 in myc expressing cells exposed to R1881.
3. The authors are using 50uM of bicalutamide in DUCaP, it is well reported that 10uM of bicalutamide exert an agonist effect on AR

4. PSA is a sentinel marker for androgen receptor activation, a pharmacodynamic marker for castration, and the marker for CRPC, the author should show the effect of Hes6 on circulating PSA (Fig. 1C). LNCaP xenografts grow at different rate, how the authors monitored the growth and time for castration. It's known that you can have large LNCaP tumors and PSA low. What was the parameter deciding for castration?
5. Luminescence jumped 3-fold in one week (5-6 week) in Hes6 castrated arm; how the authors explain this? Is this effect dependent on AR activation pathway or a consequence of activation of oncogenic pathway?
6. What is the Hes6 level in AR WT harboring cells as well as in AR negative cells.
7. Figure 1C right panel showed increase of luciferase 3 weeks after castration and the luminescence didn't change over time, this is not normally observed with the LNCaP model. Does the empty vector impede development of CRPC?
8. The authors should report the measurement of tumor volume.
9. In figure 1E, it is not clear if the mice were castrated or not
10. The authors should show the expression of AR as well as its localization after Hes6 gain and loss of function in the presence or absence of hormone as well as in the presence and absence of myc to further support a functional role of Hes6 in castration.
11. The authors showed that Hes6 increases proliferation only in castrated mice but not in intact mice, how the authors explain this data.
12. Does Hes6 increase cell proliferation in vitro in castrated condition in vitro or only in vivo. The authors should provide these data.
13. Does the microenvironment affect tumor growth in the presence of Hes6?
14. Since Hes6 has been involved in neurogenesis and neuroendocrine prostate cancer, does overexpression of Hes6 in LNCaP in castrated environment induces increase of neuronal/neuroendocrine markers?
15. It is interesting to note that genes that are regulated by Hes6 are part of genes regulated neuroendocrine cancers such as cell cycle network and Aurora kinases, the authors should discuss this and further confirm that their model is truly CRPC or neuroendocrine model.
16. If Hes6 regulates induce rapid initiation of cell cycle activity, why it was no difference in Hes6 overexpressing xenografts in intact conditions.
17. The rationale of choosing PLK-1 as a readout of Hes6-associated gene is not clear or strong. The authors should provide a strong rationale.
18. Does Hes6 regulates PLK1? Does targeting PLK1 regulates Hes6
19. The effect of GSK461364A is very profound which could be a consequence of toxicity, the author should report the effect of this drug on body weight.
20. The data showed that targeting PLK1 inhibits CRPC DEVELOPMENT, is targeting PLK1 affect tumor growth in CRPC.
21. The authors should show that the drug hits the target in xenografts.
22. Is GSK461364A induces apoptosis or inhibits proliferation in vivo

1st Revision - authors' response 04 February 2014

Referee #1 (Remarks):

The manuscript by Ramos-Montoya et al. investigates the impact of Hes6, which is over-expressed in castration resistant / metastatic prostate cancer, on the androgen receptor (AR) transcriptome and therapeutic response in prostate cancer cells. Major findings are that:
1. Hes6, which is itself up-regulated by AR or c-myc, modifies ligand-independent AR chromatin binding and regulation of gene expression shifting the pattern towards an E2F1-dominated profile.
2. Hes6 promotes resistance to castration or the AR antagonist bicalutamide.
3. The Hes6-modified AR transcriptome is associated with aggressive prostate cancer and predicts biochemical recurrence.
4. A PLK1 inhibitor restores therapeutic responsiveness to Hes6 overexpressing cells.

On balance, this is a convincing and significant study. However, there are several misleading or vague statements and/or interpretations of data as well as some inconsistencies in the experiments, which need to be addressed.

We thank reviewer 1 for their positive comments and have endeavoured to address the several points raised below.

Specific comments:
1. In Fig. 1B, it is not clear what comparisons are being made and which of the groups are significantly different.

The legend and figure panel have been amended to state that this is a comparison of Hes6 mRNA levels for aggressive prostate cancer (CRPC or mets) versus benign or primary disease. The legend now reads:

*"(B) Hes6 expression is increased in metastatic disease (mets) or castrate-resistant prostate cancer (CRPC) in multiple public datasets (Grasso et al, Taylor et al, Tomlins et al and Varambally et al). *p<0.05 for comparison of CRPC/Mets to other groups (Primary or Benign)."*

2. Hes6 overexpression does not affect tumor xenograft growth when androgen is present (intact mice, Fig. 1C). Fig. 1E is conducted in intact mice but should be done in castrated mice to determine whether Hes6 knock down blocks castration-resistant growth of C4-2b tumors.

This experiment has also been performed in castrated mice with the results shown here and added to Figure E2.

The end of the second results paragraph has been amended to read:
"Knock-down of Hes6 by lentiviral shRNA in C4-2b cells (FigE2E), led to significant attenuation of tumour growth in both full and castrated conditions (Fig. 1E and Fig E2F)."

F

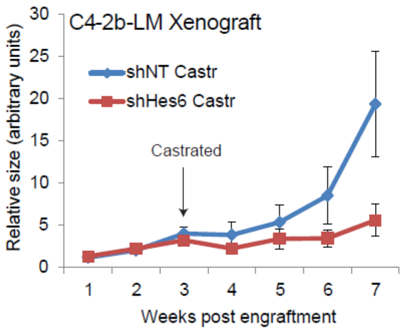

The following legend detail has been added:
"(F) Androgen insensitive C4-2b-LM xenografts showed growth attenuation in castration with constitutive lentiviral knockdown of Hes6 (shHes6) compared to Non-Targeting controls (shNT). n=5; error bars represent mean ± SEM; p<0.05 at wk 7."

3. Does Hes6 knock down block Myc-mediated cell growth in the presence of bicalutamide?

Transient knockdown of Hes6 in doxycycline-induced Myc-overexpressing LNCaP cells did reduce proliferation in bicalutamide at 30h and 48h after siRNA transfection. This has been added to Fig E1 and a reference added in the text.

The end of the first results paragraph now reads:
"[…] *confirmed that overexpression of c-Myc induces resistance to AR antagonism (Fig E1E), an effect that can be reversed by knockdown of Hes6 (Fig. E1F)."*

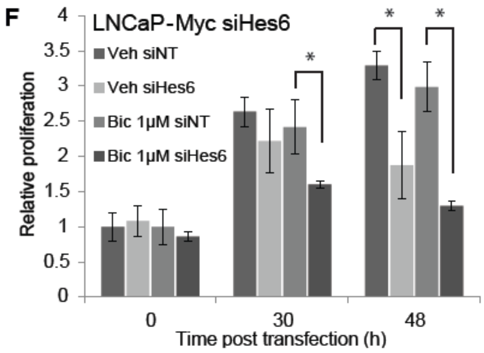

We also show here the levels of Hes6 knock-down (95 %) achieved in these cells

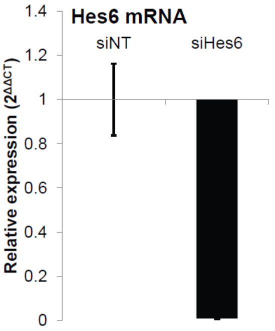

4. Supplementary Fig. S2A does not show significant differences between LNCaP and the bicalutamide resistant derivative with respect to Hes6 mRNA levels. The statement in text is misleading and needs to reflect the lack of a significant difference between these cells.

We have amended the text to reflect this. It now reads:
"Using the bicalutamide/castration-resistant derivatives LNCaP-Bic, C4-2 and C4-2b cells, as models of CRPC, we found evidence of significant increases in transcript levels of Hes6 in the C4-2 derivatives compared to LNCaP controls (Fig. E2A)"

5. The authors should explain more clearly how data in Figs. S2C and D were analyzed.

In Fig E2C and D we assessed the rate of change for each condition at the mid-point of each experiment and plotted these values on the bar chart as shown. This legend has been amended to make this clearer. In addition, we have changed the units on the y-axis of the DUCaP bar chart for consistency across both plots.

6. The images in Fig. S3A are inadequate for demonstrating sub-cellular localization of AR. The repeated claim that Hes6 drives increased nuclear localization should be removed or additional data / experiments need to be shown.

We acknowledge that we have not investigated the mechanisms of AR localisation from cytoplasm to nucleus here. Rather we have simply observed the relative density of nuclear AR with and without Hes6 in full and castrated mice. We feel that the images (high resolution shown below), but more particularly the Ariol Imagestream quantification do demonstrate this difference in nuclear density even after 12 weeks of castration.

We have amended the legend for this figure and the text to modify this claim.
"We next assessed intensity of nuclear AR and cell proliferation by immunohistochemical analysis of the LNCaP grafts."

We have also removed the word localisation in the reference to this in the section entitled:
"Hes6, AR and E2F1 co-operate in driving a cell-cycle-related tumour-enhancing network"

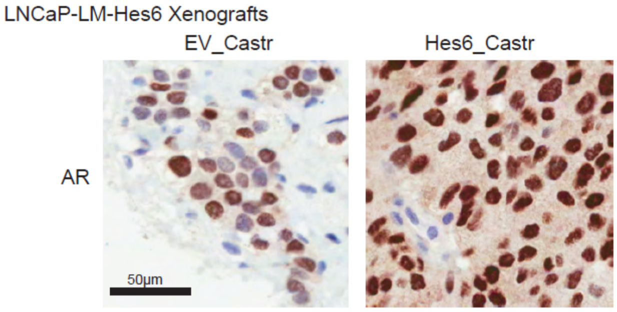

7. Does Hes6 interact with AR?

We have not found any evidence for a direct protein interaction between Hes6 and AR in prostate cancer cell lines. However, we have seen an interaction between AR and Hes6 when the two of them are exogenously expressed in COS7 cells.

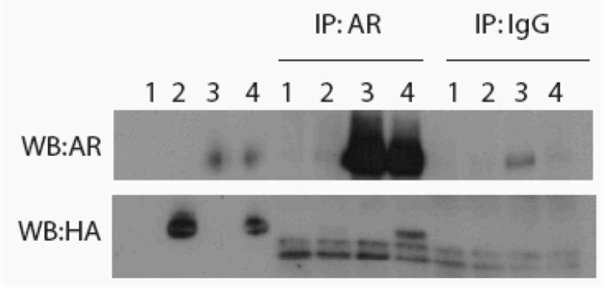

Cos7 cells were transfected and immunoprecipitation was performed using both monoclonal AR antibody and a mouse IgG antibody. The western blot shows the result: lane 1 empty vector, lane 2 Hes6, lane 3 AR and lane 4 AR and Hes6. A specific band was observed indicative of a Hes6/AR interaction *in vitro*.

8. The effects of Hes6 on cell cycle (Fig. S8B) are very modest. It is unclear whether such effects can account for the differences observed in in vivo tumor growth. This needs to be further addressed experimentally.

We do find evidence for a significant change in the fraction of cells in each phase of the cell cycle with a 13% reduction in cell fraction in G1 accounted for by a 22% increase in S phase and 300% increase G2/M phase with over-expression of Hes6 (three-fold: mean 4.4% to 12.6% of cells). Although only one representative FACS plot has been shown, we have quantified the biological replicates in the bar chart in Fig E8A and include here the actual values for information. We have further addressed this experimentally *in vitro* with the synchronisation experiment included in panels C and D and have assessed proliferation *in vivo* by Ki67 staining included in Figs E3A and E3C. We do not intend to claim that the effect on cell cycle is the sole difference with Hes6 but part of a bigger picture of persistent AR and E2F1 signalling. Furthermore, there seems to be evidence in the literature for similar changes having a significant impact on cell growth (percentage difference in S phase, single integer fold changes in G2/M fractions) being relevant for cell growth (Go et al, 2000; Yu et al, 2007) and we suspect that such a change may have an impact over the course of a 3 month *in vivo* experiment.

	Single cells,%G1	Single cells,%S	Single cells,%G2
1: LNCaPEV_1.fcs	74.1	21	3.19
2: LNCaPEV_2.fcs	78	20.9	3.32
3: LNCaPEV_3.fcs	76.7	18.3	6.89
Mean	76.26667	20.06667	4.466667
StdDev	1.985783	1.530795	2.099675
4: LNCaPHes6HA_1.fcs	64.5	25.1	10.7
5: LNCaPHes6HA_2.fcs	67.9	24.8	11.8
7: LNCaPHes6HA_3.fcs	65.3	24.1	13.4
Mean	66.6	24.45	12.6
StdDev	1.838478	0.494975	1.131371
p value	0.002529	0.007845	0.006537

9. The statistical analysis of Fig. 4G is unclear. At which time points are there significant differences?

We have explained this more clearly in the legend and expanded methods. Statistical significance was determined by comparison of the divergent confluence curves at the end of the experiment.

10. What is the mechanistic relationship between Hes6 and PLK1 in prostate cancer?

We acknowledge that this is still unknown. However, for the first time we have seen the relationship between Hes6 over-expression and PLK1 and that PLK1 is part of the E2F1 regulome which is enhanced by Hes6 overexpression in castration. We have also shown high PLK1 (protein and mRNA) and Hes6 in human prostate tumour.

Minor comments:
1. In Significance section, the comment "...the physical interaction between the AR and Hes6 with E2F1..." requires clarification as it seems to infer that a ternary complex is formed.

We have not demonstrated a ternary complex and have therefore clarified the text which now reads:
"[...] the protein interaction between the AR and E2F1 and between Hes6 and E2F1."

2. In 1st part of the Results section, the phrase "...overexpression of c-Myc rescues bicalutamide induced AR antagonism" is confusing and should be restated.

This has been rephrased to:
"overexpression of c-Myc overcomes growth inhibition by bicalutamide"

3. Why is there a discrepancy in the extent of R1881-induced up regulation of Hes6 mRNA in LNCaP cells comparing Fig. 1A (approx. 3-fold) vs. S1B (less than 2-fold)?

These were two different experiments and we suspect that the differences are due to minor technical differences (the first experiment was collected at 24hrs, the second at 12hrs – we find that Hes6 up-regulation occurs from 6hrs onwards, with maximal induction at 24hrs). The up-regulation is of the same order of magnitude and still statistically significant. A newly introduced third plot (see Reviewer 3 comment 1 below) shows an approximately 6-fold increase in Hes6 mRNA levels.

Referee #2 (Comments on Novelty/Model System):

The authors need to test the growth response of an AR non expressing prostate cancer line (e.g., PC-3) to the PLK-i in order to document that such response actually requires AR signaling. Without this data, no definitive conclusion are possible as to the relationship between PLK inhibition as a readout of AR/HES6 interaction.

We thank the reviewer for this suggestion and have carried out the experiment recommended.

We found that GSK461364A does induce some growth inhibition of PC3 cells at 7.5nM as expected given the M phase centromere role of PLK1. However, interestingly, on introduction of the AR to this AR non-expressing line we found evidence for an increase in efficacy which actually supports the possibility of an AR-related role for PLK1 in this mechanism of castration resistance.

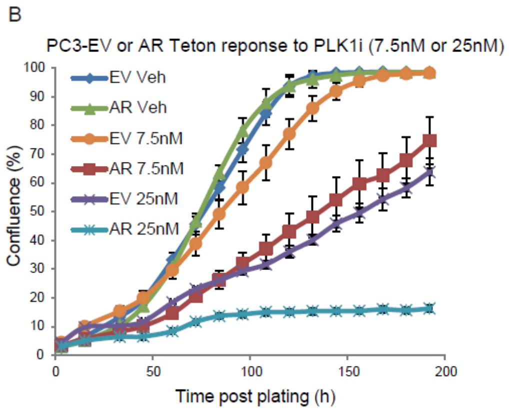

These data are now included in Fig E12B and text has been added to the final results paragraph of the manuscript.
"PLK1 inhibition also reduced growth of castrate resistant AR-positive cells (C4-2b) and AR-negative cells (PC3) (Fig E12B-C) with greater effects on PC3 cells on isogenic introduction of the AR."

In our paper we do not claim that the main effects of PLK1 are dependent on the AR. Inhibition of PLK1 will likely cause generalised growth reduction in dividing cells. Rather we propose that when conventional AR antagonism is no longer effective, but the AR is still functionally important, the inhibition of PLK1 can disrupt the cell cycle activity to reduce cell growth and that this could provide a useful therapeutic approach.

Referee #2 (Remarks):

There are only a few suggestions for improvement.
First, while bioluminescence is needed for orthotopic tumor evaluation, when tumors are growing subcutaneously in the flank, micro-caliper measurements are more accurate and should be used. This is particularly relevent with AR expressing tumors because AR signaling effects tumor angiogenesis and changes in tumor vascularity effect delivery of luciferin. Thus, bioluminescence is not simply a relection of tumor size but also tumor perfusion.Thus , real tumor volumes should be reported in Fig 1F, 1E, and Fi 4G.

We appreciate that there are different ways to measure tumour burden in the xenograft setting. We feel that bioluminescent imaging is a legitimate approach for subcutaneous grafts and, when combined with final tumour weights (and images) as shown in Fig 1C, provides an adequate reflection of tumour growth. We also calibrate each of our cell lines to establish a linear relationship between cell number and BLI output (see below). We acknowledge the importance of tumour vascularity in delivery of luciferin and incorporate in our analysis protocol a time-lapse multi-image approach from 8 to 20 minutes post injection in order to accommodate a change in timing of maximal bioluminescent output. We have discussed the matter with our responsible officers in the mouse facility and they have advised us that it would not be ethically correct to repeat these experiments to obtain the calliper data. However, we will adopt this advise for future experiments, and calliper measurements will always be collected in parallel to the bioluminescence imaging. We have performed CD31 immunohistochemistry on the grafts in Figure 1 and found an increase in tumour vascularity with Hes6 in castration. We have not included these images in the paper as we do not feel that they add anything substantial (we would expect growing grafts to have better vessel infiltration), however we would be happy to include these data if requested. Indeed, if the output of our bioluminescence analysis was caused by a better vascularization of the Hes6 castrated tumours,

it would still be an encouraging readout of the effects of Hes6 overexpression on the resistance of LNCaP xenografts to castration.

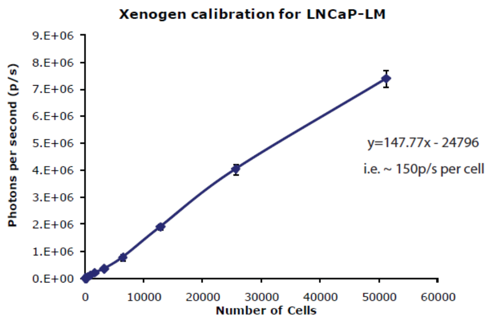

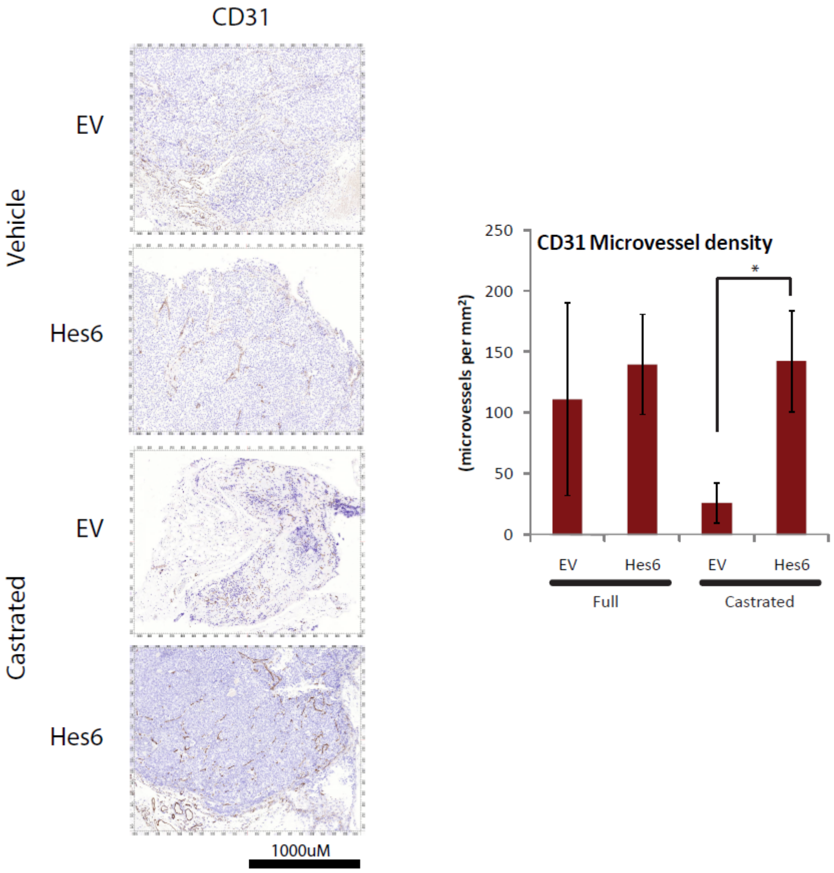

Finally we would also like to refer the reviewer to our recent publication (Massie et al, 2011) where we use the same approach in monitoring xenograft growth.

Referee #3 (Comments on Novelty/Model System):

The authors used a sophisticated tools to interrogate he role of Hes6 in prostate cancer using ChIP assays, however they didn't clarify the rational of choosing Hes6. Moreover it was surprising that thy didn't investigate or discuss the potential role in neuroendocrine prostate cancer since it's known that Hes6 play a role in stem cells neurogenesis. This manuscript could be with big interest if it was explored in the context of neuroendocrine prostate cancer.

We thank the reviewer for their constructive comments and questions about the broader context of this work.

We agree that it is important to make clear the rationale for studying Hes6 in the first instance. We have presented data from cell lines (Fig E2A) and clinical datasets (Fig 1B) demonstrating the increased levels of Hes6 in aggressive disease. In addition we would draw the attention of the reviewer to previous findings of our lab (Vias et al, 2008) where Hes6 was found to be upregulated in the p53pRB null transgenic model of prostate cancer, which has castrate-resistant properties. This is referenced in the introduction. We agree that neuroendocrine prostate cancer is important, especially given its increasing incidence in the context of increasingly effective AR therapy (Li et al, 2013). Interestingly, we thought initially that we would find a neuroendocrine phenotype in our model of castration resistance given the emphasis of the small amount of published Hes6 literature and our own previous findings (discussed in the penultimate paragraph of the introduction). However, we found consistently that there was no clear evidence of change in phenotypic markers (this data is presented in Fig E5) and therefore sought an alternative explanation for the functional effects of Hes6 overexpression. We acknowledge that this was not made clear in the text and have therefore added some additional explanation in the results section entitled "Hes6, AR and E2F1 co-operate in driving cell-cycle-related tumours-enhancing network".

The text now reads:
"Surprisingly, we did not find any evidence for a neuroendocrine phenotype (Fig E5) but rather a predominance for cell cycle pathways. A combined cell cycle network from the input gene list revealed several dominant clusters including E2Fs, CDKs, Cyclins, and Aurora kinases (AURK) (Fig. E6B)."

We also suspect there may be a broader incidence of NE characteristics (as opposed to those tightly defined by as being SYP^{+} $CHGA^{+}$ AR^{-} tumours) as demonstrated, for example by Beltran et al (Beltran et al, 2011) with AURKA and Myc. We have now referenced this paper with a comment in our discussion.

"Although we did not find evidence for a classical neuroendocrine phenotype, recent studies have suggested a broader relevance for neuroendocrine differentiation with powerful transcription factors such as Myc and cell-cycle regulators such as AURKA being implicated in development of therapy resistant tumours bearing neuroendocrine characteristics (Beltran, 2011)"

Referee #3 (Remarks):

In this manuscript, the authors explored the role of Hes6 in prostate cancer and showed that Hes6 is up-regulated in aggressive phenotype and drives castrate resistant prostate cancer in ligand independent manner. They found that Hes6 maintains AR binding to cell cycle regulatory genes under the control of E2F1. They went further and analyzed Hes6 regulome and found that PLK1 could be a potential target for prostate cancer. Hes6 is a transcription co-factor, previously described as a regulator of stem cells during neurogenesis and as a marker for neuroendocrine prostate cancer. The manuscript describes novel findings however, some data are confusing and others requires more depth and supporting information. The conclusion of the manuscript is overstated.

We have addressed these areas of confusion where outlined below and moderated the conclusions of the manuscript as a response to the reviewer's comments raised here.

In neuroendocrine prostate cancer, androgen receptor (AR) is lost and AR dependent genes are inhibited, one will argue that a neuroendocrine marker should be negatively regulated by androgen however the authors showed that androgens regulate positively Hes6 and bicalutamide decreases it. They went and showed that in myc expressing cells, Hes6 is increased with bicalutamide. The reviewer is confused and having hard time to understand the opposite data. However it's clear that myc cooperate with AR inactivation to enhance Hes6 expression.

Please see our comments about the neuroendocrine phenotype above. Importantly, we are not claiming that Hes6 is a neuroendocrine marker, nor do we think that it has been stated to be so in the published literature. We suspect that some genes previously shown to be involved in neuroendocrine

differentiation are actually more involve as generic cell-cycle drivers (e.g. Hes6), whereas others are more indicative of differentiation (e.g. SYP or CHGA). We've confirmed that the AR is present and chromatin-bound in our model and found Hes6 to be androgen regulated. We acknowledge the confusion raised in Fig 1A and have sought to address this by carrying out the experiment recommended by the reviewer (comment 2 below) and have modified the panel accordingly. Interestingly, we have not found any direct evidence for cooperation between Myc and the AR, although both do upregulate Hes6 expression as shown (Fig 1A).

1. LNCaP cells harbor a mutation in AR making them resistant to bicalutamide, does targeting AR with Enzalutamide regulates Hes6 expression

In our hands, LNCaP cells respond to doses of bicalutamide greater than 1 µM, and become resistant after 3 weeks of growing in the presence of this concentration.
We have performed the recommended experiment and have found that Hes6 expression is also reduced by AR inhibition with MDV3100.

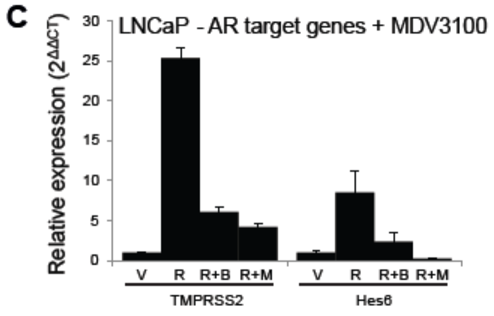

These data are now included in Fig E1C with appropriate addition to the legend as shown below:
"(C) Hes6 and TMPRSS2 (representative AR target) mRNA levels with enzalutamide 10µM (MDV3100, M) or bicalutamide 10µM (B); n=3, error bars represent mean ± SEM. All p<0.05 (AR inhibitor compared to R1881)."

2. The authors should show the expression of Hes6 in myc expressing cells exposed to R1881.

We have performed this experiment as recommended and have included the results in Fig E1D. In addition we have simplified the first panel in Fig 1A to show Myc up-regulation of Hes6 in full serum conditions (first graph bellow).

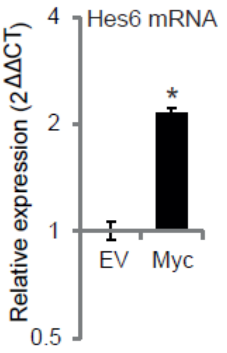

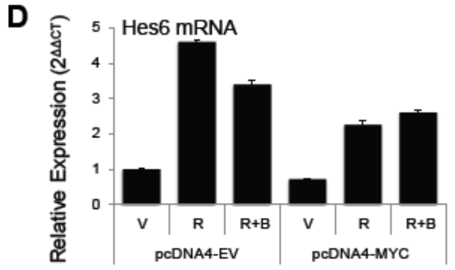

The response to R1881 was necessarily assessed on a charcoal stripped background and we have included bicalutamide treatment here. The upregulation of Hes6 mRNA is seen here with R1881 in EV and, to a lesser extent, with Myc. On addition of bicalutamide to inhibit the AR, Hes6 levels are reduced in EV as expected but, interestingly, to a lesser degree with Myc, suggesting that Myc may maintain Hes6 levels in the absence of AR activity. Intriguingly, with a charcoal stripped background Myc does not increase Hes6 level in the absence of either hormone or inhibitor. We hope this helps address the confusion raised above. Given that this is not an essential element of our paper we have removed this detail from the main figure and placed it in supplementary data. The reviewer will notice that it was not our intention to address the question of AR and Myc interaction in our original manuscript and nor have we sought to discuss this any further in the revised version.

We feel that this is a question for a future study. Our intention here was simply to show that these two important oncogenes (AR and Myc) up-regulate Hes6 levels.

3. The authors are using 50uM of bicalutamide in DUCaP, it is well reported that 10uM of bicalutamide exert an agonist effect on AR

In our hands DUCaP cells are less responsive to bicalutamide when compared to LNCaPs and this was the dose required to see an effect on cell growth. We note evidence for a wide range of bicalutamide doses with progressive inhibition in prostate cancer cell lines up to 100µM (Masiello et al, 2002).

4. PSA is a sentinel marker for androgen receptor activation, a pharmacodynamic marker for castration, and the marker for CRPC, the author should show the effect of Hes6 on circulating PSA (Fig. 1C). LNCaP xenografts grow at different rate, how the authors monitored the growth and time for castration. It's known that you can have large LNCaPtumors and PSA low. What was the parameter deciding for castration?

We castrated the tumours once they reached a pre-defined size of approximately 100 mm^3 (Andersen et al, 2010). We confirmed efficacy of castration by performing IHC on host prostates and confirming increased levels of cytoplasmic AR (Fig E3A). Blood was not collected from this experiment, therefore PSA cannot be analysed and we are advised that it would not be ethical to repeat this experiment for this additional measurement. However we note the reviewers comment and this would be of interest in future experiments.

5. Luminescence jumped 3-fold in one week (5-6 week) in Hes6 castrated arm; how the authors explain this? Is this effect dependent on AR activation pathway or a consequence of activation of oncogenic pathway?

We think the small initial response to castration in the Hes6 cells occurs while the cells adapt to the modified environment during which the alternate survival pathways are initiated. We believe this is a consequence of maintained AR chromatin binding (Figure 2). We believe this is AR dependent as the Hes6-overexpressing cells do not survive AR knockdown (Fig 2G).

6. What is the Hes6 level in AR WT harboring cells as well as in AR negative cells.

We include below relative Hes6 expression levels across a panel of endocrine regulated cancer cell lines: AR negative prostate cancer (PC3 and DU145), AR positive prostate cancer (22Rv1, VCaP, DuCaP, LNCaP and C4-2b), breast cancer (MCF-7) and ovarian cancer (SKOv3).

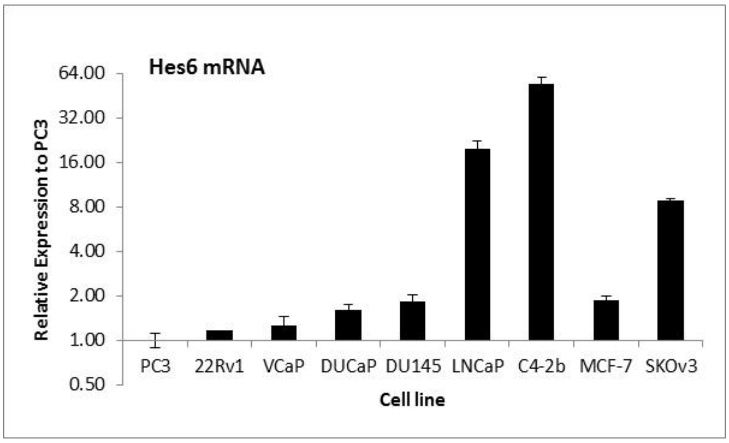

7. Figure 1C right panel showed increase of luciferase 3 weeks after castration and the luminescence didn't change over time, this is not normally observed with the LNCaP model. Does the empty vector impede development of CRPC?

We believe the LNCaP model to be an androgen-dependent and castration sensitive line and, in our hands, LNCaP grafts always respond initially to castration and do not become resistant until

approximately 12 weeks. We feel this is consistent with data generally available (Dahiya et al, 1995; Thalmann et al, 1994).

8. The authors should report the measurement of tumor volume.

We appreciate that there are different ways to measure tumour burden in the xenograft setting. We feel that bioluminescent imaging is a legitimate approach for subcutaneous grafts, and when combined with final tumour weights (and images) as shown in Fig 1C provides an adequate reflection of tumour growth. We have discussed the matter with our responsible officers in the mouse facility and they have advised us that it would not be ethically correct to repeat these experiments to obtain the calliper data. However, we will adopt this advise for future experiments, and calliper measurements will always be collected in parallel to the bioluminescence imaging.

9. In figure 1E, it is not clear if the mice were castrated or not

This experiment has also been performed in castrated mice, with the results shown here and added to Figure E2.

The end of the second results paragraph has been amended to read:
"Knock-down of Hes6 by lentiviral shRNA in C4-2b cells (FigE2E), led to significant attenuation of tumour growth in both full and castrated conditions (Fig. 1E & Fig E2F)."

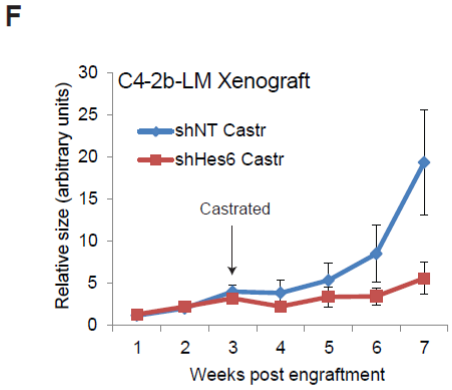

The following legend detail has been added:
"(F) Androgen insensitive C4-2b-LM xenografts showed growth attenuation in castration with constitutive lentiviral knockdown of Hes6 (shHes6) compared to Non-Targeting controls (shNT). n=5; error bars represent mean ± SEM; p<0.05 at wk 7."

10. The authors should show the expression of AR as well as its localization after Hes6 gain and loss of function in the presence or absence of hormone as well as in the presence and absence of myc to further support a functional role of Hes6 in castration.

We acknowledge that we have not investigated the mechanisms of AR localisation from cytoplasm to nucleus here. Rather we have simply observed the relative density of nuclear AR with and without Hes6 in full and castrated mice. We feel that the images (high resolution shown below), but more particularly the Ariol Imagestream quantification, do demonstrate this difference in nuclear density even after 12 weeks of castration.
We have amended the legend for this figure and the text to modify this claim.
"We next assessed intensity of nuclear AR and cell proliferation by immunohistochemical analysis of the LNCaP grafts."

We have also removed the word localisation in the reference to this in the section entitled:
"Hes6, AR and E2F1 co-operate in driving a cell-cycle-related tumour-enhancing network"

© EMBO

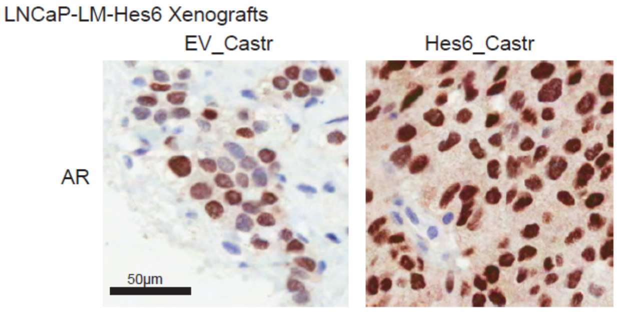

Further to this, we have performed the experiment requested to investigate expression of AR in these various conditions. We had already reported in the paper that Hes6 overexpression does not change AR expression (see Figure E5A-B). Using a doxycycline-inducible Myc overexpressing cell line (LNCaP) we have assessed AR expression in either full serum conditions (with and without 10µM bicalutamide) in the presence of Hes6 siRNA knockdown (Panel A) or in charcoal stripped conditions in the presence or absence of 1nM R1881 hormone (Panel B).

In this model, the siRNA knockdown of Hes6 had a dramatic effect on AR expression, and this effect was not compensated with the bicalutamide treatment or enhanced with the R1881 treatment. Myc overexpression decreased the levels of AR expression and reduced to the bare minimum the induction of AR expression after treatment with bicalutamide. R1881 treatment decreased AR expression irrespective of Myc levels.

A.

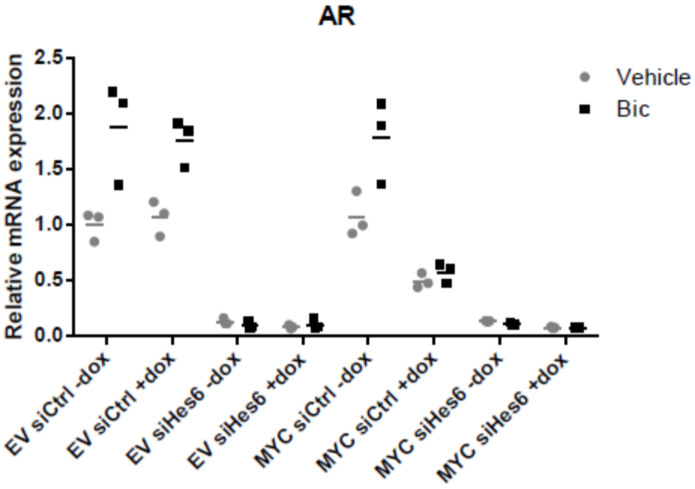

B.

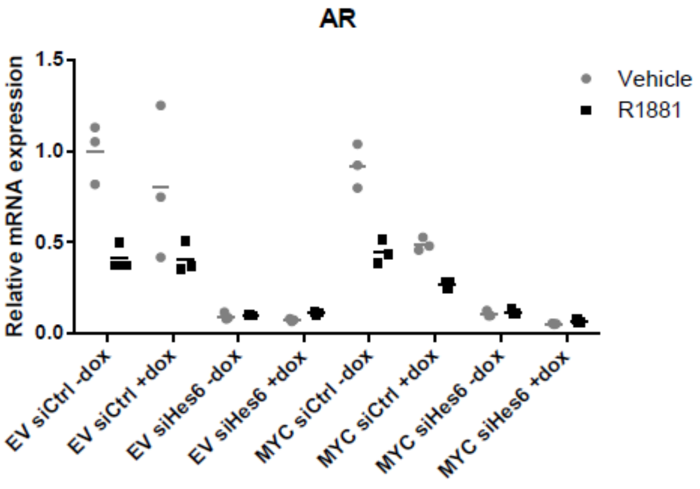

11. The authors showed that Hes6 increases proliferation only in castrated mice but not in intact mice, how the authors explain this data.

We believe that, although Hes6 transcription is increased by the AR in full conditions, it is held in reserve for when conventional AR activity is inhibited and therefore is not crucial for cell-growth during normal androgen conditions. Nonetheless we did find a small difference in Ki67 staining in full conditions (Fig E3), although this did not lead to any recordable difference in growth.

12. Does Hes6 increase cell proliferation in vitro in castrated condition in vitro or only in vivo. The authors should provide these data.

Hes6 maintains cell proliferation *in vitro* in the presence of bicalutamide (Fig E2C).

13. Does the microenvironment affect tumor growth in the presence of Hes6?

We do not know the answer to this as it has not been investigated in this study.

14. Since Hes6 has been involved in neurogenesis and neuroendocrine prostate cancer, does overexpression of Hes6 in LNCaP in castrated environment induces increase of neuronal/neuroendocrine markers?

Please, refer to the comments addressed to this reviewer at the beginning of the letter.

15. It is interesting to note that genes that are regulated by Hes6 are part of genes regulated neuroendocrine cancers such as cell cycle network and Aurora kinases, the authors should discuss this and further confirm that their model is truly CRPC or neuroendocrine model.

Please, refer to the comments addressed to this reviewer at the beginning of the letter.

16. If Hes6 regulates induce rapid initiation of cell cycle activity, why it was no difference in Hes6 overexpressing xenografts in intact conditions.

We refer the reviewer to the response to comment 11 above regarding proliferation, Ki67 activity and cell growth. We agree that although there is an increase in FACS cell cycle progression *in vitro* this does not lead to any change in physiological tumour growth.

17. The rationale of choosing PLK-1 as a readout of Hes6-associated gene is not clear or strong. The authors should provide a strong rationale.

PLK1 was selected amongst a number of candidates as a member of the 222 list of genes found to be associated with increased levels of Hes6 in xenografts and in human tissue and we chose to work with PLK1 specifically due to the availability of small molecule inhibitors.

18. Does Hes6 regulates PLK1? Does targeting PLK1 regulates Hes6

We report the upregulation of PLK1 on overexpression of Hes6 (Fig 3A) and we also find high levels of PLK1 in tumours stratified by high levels of Hes6 (Fig E10A).

We have performed an additional experiment to assess whether targeting PLK1 regulates Hes6 and find no significant effect on Hes6 mRNA expression with three PLK1 siRNAs (see panel A below for PLK1 knockdown and panel B for Hes6 expression) or GSK461364A (panel C).

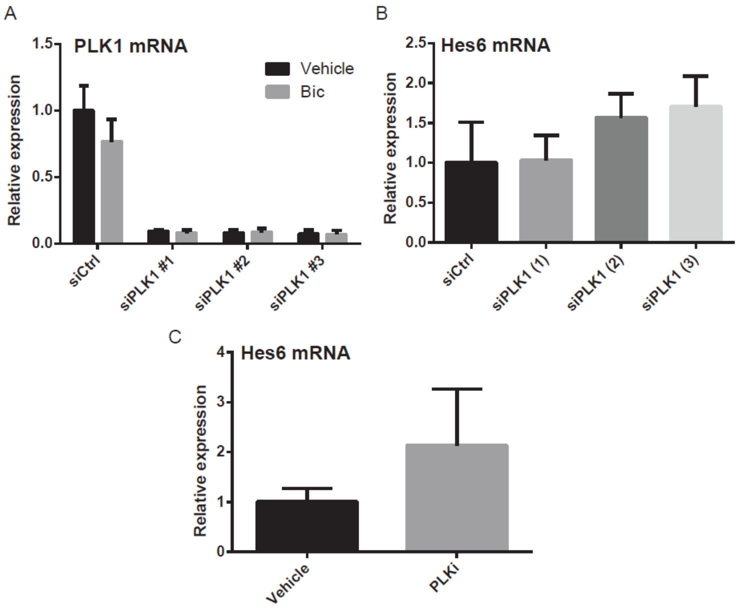

19. The effect of GSK461364A is very profound which could be a consequence of toxicity, the author should report the effect of this drug on body weight.

The final weights for the two groups of castrated mice as a percentage of starting weight were 96% (± 1.74) and 92% (±3.79) for the vehicle and drug group respectively indicating a small reduction in body weight for the drug group but both groups are well within our Home Office compliant permissible weight reduction for mice undergoing xenograft or drug treatment. Furthermore there were no other health issues (skin changes, appetite reduction, GI upset, abnormal behaviour etc) suggestive of toxicity.

© EMBO

20.The data showed that targeting PLK1 inhibits CRPC DEVELOPMENT, is targeting PLK1 affect tumor growth in CRPC

We have addressed this by assessing the effect of PLK1 inhibition with GSK461364A and three siRNAs in 'established' castrate resistant C4-2b cells. This data is now presented in Fig E12C and shown below.

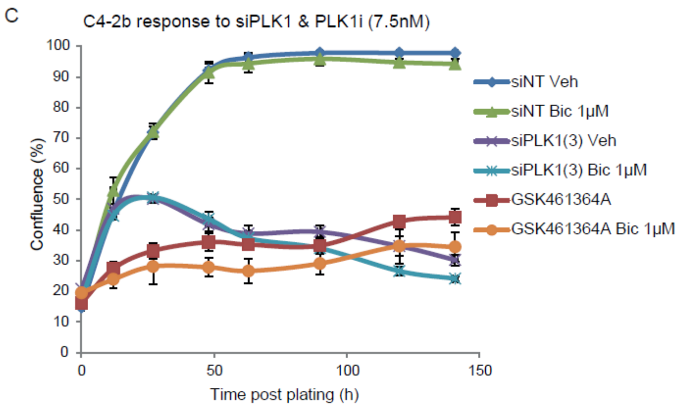

An additional sentence has been added to the text of the final results paragraph to now read: *"PLK1 inhibition also reduced growth of castrate resistant AR-positive cells (C4-2b) and AR-negative cells (PC3)(Fig E12B-C)"*

21. The authors should show that the drug hits the target in xenografts.

We refer the reviewer to the work of our collaborators at GSK who have thoroughly assessed the pharmacokinetic properties and bioavailability of this compound in the xenograft setting (Gilmartin et al, 2009).

22. Is GSK461364A induces apoptosis or inhibits proliferation in vivo

We have investigated this by immunohistochemistry for Ki67 and cleaved caspase 3 (CC3) on the xenografts treated the PLK1i. The data is shown here. We find a dramatic change in Ki67 staining as expected between the large castration resistant grafts and those inhibited by PLK1i, suggesting a reduction in proliferating cells. There is no evidence here for apoptosis. Although there is some background staining in these images, there is virtually no nuclear staining of CC3 in either xenograft (two positive nuclei in vehicle, zero in PLK1i; positive control included by way of comparison).

© EMBO

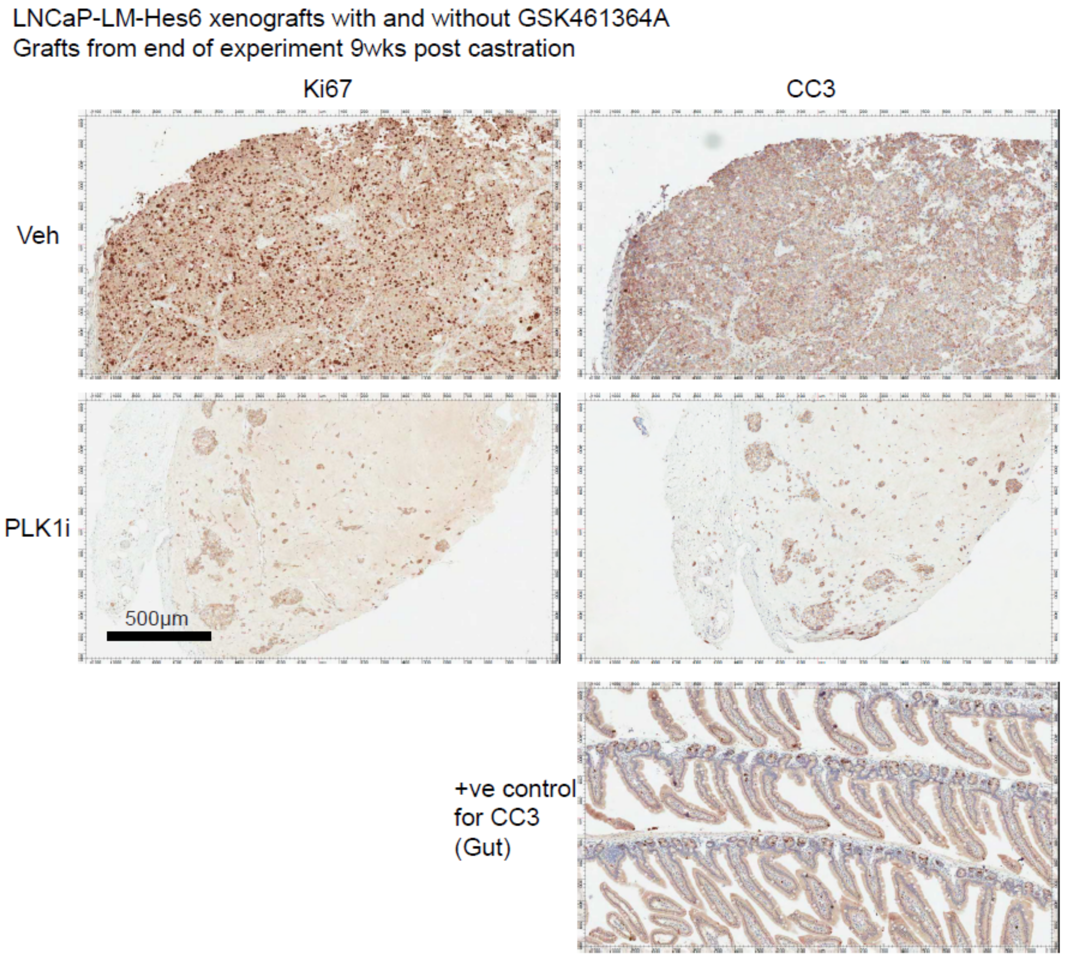
LNCaP-LM-Hes6 xenografts with and without GSK461364A
Grafts from end of experiment 9wks post castration
Ki67
CC3
Veh
PLK1i
500µm
+ve control
for CC3
(Gut)

References

Andersen RJ, Mawji NR, Wang J, Wang G, Haile S, Myung JK, Watt K, Tam T, Yang YC, Banuelos CA et al (2010) Regression of castrate-recurrent prostate cancer by a small-molecule inhibitor of the amino-terminus domain of the androgen receptor. Cancer Cell 17**:** 535-546

Beltran H, Rickman DS, Park K, Chae SS, Sboner A, MacDonald TY, Wang Y, Sheikh KL, Terry S, Tagawa ST et al (2011) Molecular characterization of neuroendocrine prostate cancer and identification of new drug targets. Cancer discovery 1**:** 487-495

Dahiya R, Zhang DY, Ho RJ, Haughney PC, Hayward SW, Cunha GR, Narayan P (1995) Regression of LNCaP human prostate tumor xenografts in athymic nude mice by 13-cis-retinoic acid and androgen ablation. Biochem Mol Biol Int 35**:** 487-498

Gilmartin AG, Bleam MR, Richter MC, Erskine SG, Kruger RG, Madden L, Hassler DF, Smith GK, Gontarek RR, Courtney MP et al (2009) Distinct concentration-dependent effects of the polo-like kinase 1-specific inhibitor GSK461364A, including differential effect on apoptosis. Cancer Res 69**:** 6969-6977

Go C, He W, Zhong L, Li P, Huang J, Brinkley BR, Wang XJ (2000) Aberrant cell cycle progression contributes to the early-stage accelerated carcinogenesis in transgenic epidermis expressing the dominant negative TGFbetaRII. Oncogene 19**:** 3623-3631

Li Z, Chen CJ, Wang JK, Hsia E, Li W, Squires J, Sun Y, Huang J (2013) Neuroendocrine differentiation of prostate cancer. Asian J Androl 15**:** 328-332

Masiello D, Cheng S, Bubley GJ, Lu ML, Balk SP (2002) Bicalutamide functions as an androgen receptor antagonist by assembly of a transcriptionally inactive receptor. J Biol Chem 277**:** 26321-26326

Massie CE, Lynch A, Ramos-Montoya A, Boren J, Stark R, Fazli L, Warren A, Scott H, Madhu B, Sharma N et al (2011) The androgen receptor fuels prostate cancer by regulating central metabolism and biosynthesis. EMBO J 30**:** 2719-2733

Thalmann GN, Anezinis PE, Chang SM, Zhau HE, Kim EE, Hopwood VL, Pathak S, von Eschenbach AC, Chung LW (1994) Androgen-independent cancer progression and bone metastasis in the LNCaP model of human prostate cancer. Cancer Res 54**:** 2577-2581

Vias M, Massie CE, East P, Scott H, Warren A, Zhou Z, Nikitin AY, Neal DE, Mills IG (2008) Pro-neural transcription factors as cancer markers. BMC medical genomics 1**:** 17

Yu J, Guo QL, You QD, Zhao L, Gu HY, Yang Y, Zhang HW, Tan Z, Wang X (2007) Gambogic acid-induced G2/M phase cell-cycle arrest via disturbing CDK7-mediated phosphorylation of CDC2/p34 in human gastric carcinoma BGC-823 cells. Carcinogenesis 28**:** 632-638

2nd Editorial Decision 21 February 2014

Thank you for the submission of your revised manuscript to EMBO Molecular Medicine. We have now received the enclosed reports from the referees that were asked to re-assess it. As you will see the reviewers are now globally supportive and I am pleased to inform you that we will be able to accept your manuscript pending the following final amendments/requests:

1) As per our Author Guidelines, the description of all reported data that includes statistical testing must state the name of the statistical test used to generate error bars and P values, the number (n) of independent experiments underlying each data point (not replicate measures of one sample), and the actual P value for each test (not merely 'significant' or 'P < 0.05').

2) We would need a short list (up to 5) of bullet points that summarize the key NEW findings. The bullet points should be designed to be complementary to the abstract and will be used online in our new web platform.

3) We are now encouraging the publication of source data, particularly for electrophoretic gels and blots, with the aim of making primary data more accessible and transparent to the reader. Would you

be willing to provide a PDF file per figure that contains the original, uncropped and unprocessed scans of all or at least the key gels used in the manuscript? The PDF files should be labeled with the appropriate figure/panel number, and should have molecular weight markers; further annotation may be useful but is not essential. The PDF files will be published online with the article as supplementary "Source Data" files. If you have any questions regarding this just contact me.

Please submit your revised manuscript within two weeks. I look forward to seeing a revised form of your manuscript as soon as possible.

***** Reviewer's comments *****

Referee #1 (Remarks):

The authors have responded well to my comments and concerns.

Referee #2 (Remarks):

The authors have revised the manuscript appropriately.

Referee #3 (Remarks):

The authors did a great job responding to the reviewers comments.

2nd Revision - authors' response 06 March 2014

We are delighted with the reviewers positive response and that you are now able to accept our manuscript (EMM-2013-03581) for publication in EMBO Molecular Medicine pending the final amendments requested.

- We have detailed the statistical information requested for each panel of each figure.
- We are indeed willing to provide the original blots included in the paper and have uploaded these as “Source Data” pdf files as requested. We have included varying exposures of the membranes for clarity.
- Please see below 5 bullet points outlining the key new findings:
    1. Hes6 promotes castration resistance in prostate cancer cells.
    2. Hes6 maintains AR chromatin binding at a subset of sites in the absence of hormone stimulation.
    3. Hes6-associated genes predict poor clinical outcome after radical prostatectomy.
    4. Hes6-responsive gene PLK1 is highly expressed in a new hormone relapse TMA.
    5. Inhibition of PLK1 enhances sensitivity to anti-androgens.

Please do let us know if there’s anything else we need to add. We should mention that we have high resolution versions of the pdf files that currently say “Press Quality” in the title (Figures 2 and 4). Several Expanded View figures have been similarly compressed for upload and can be provided in higher resolution if desired.
