# Supplementary material for: HES6 drives a critical AR transcriptional programme to induce castration-resistant prostate cancer through activation of an E2F1-mediated cell cycle network
Source: EMBO Mol Med. 2014 Apr 14;6(5):651–61. doi: 10.1002/emmm.201303581 (PMC4023887; doi:10.1002/emmm.201303581)
Supplement: Supplementary file 24 [file emmm0006-0651-sd24.pdf]

Figure 1C

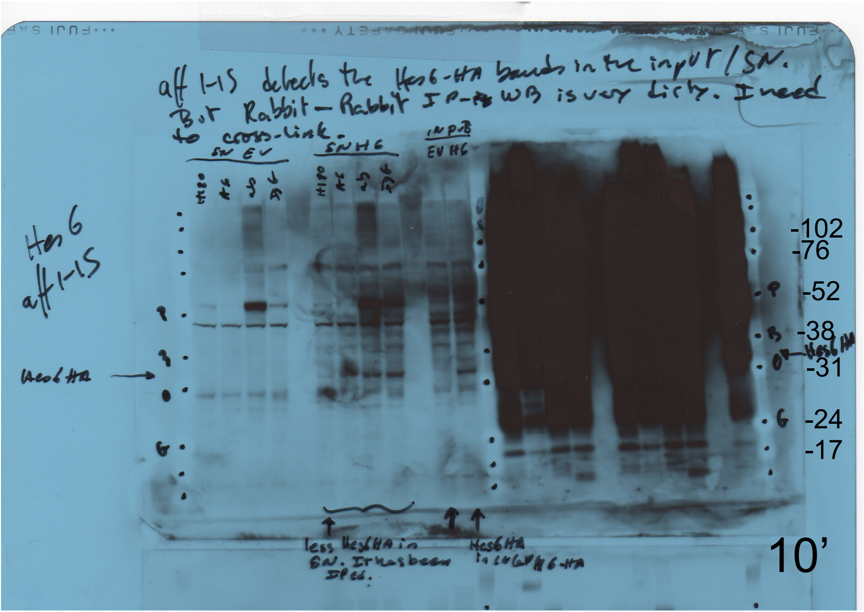

aff 1-15 detects the Hes6-HA bands in the input/SN.
But Rabbit-Rabbit IP-WB is very dirty. I need to cross-link.
SN EV
SN H6
INPUT
EV H6
Hes6
aff 1-15
Hes6 HA
-102
-76
-52
-38
-31
-24
-17
Hes6 HA
less Hes6HA in SN. It has been IPed.
Hes6HA in INPUT H6-HA
10'
